# Malonyl-Caffeoylquinic Acids and Malonyl-Flavonoid-Glucosides from Three Edible Apiaceae Plants *Anthriscus Cerefolium*, *Anthriscus Sylvestris*, and *Chaerophyllum Bulbosum*

**DOI:** 10.1021/acsomega.5c03770

**Published:** 2025-09-22

**Authors:** Adila Nazli, Mária Gáborová, Tim Ausbüttel, Bence Stipsicz, Gergő Tóth, Szilvia Bősze, Szabolcs Béni, Imre Boldizsár

**Affiliations:** † Department of Pharmacognosy, 37637 Semmelweis University, Budapest 1085, Hungary; ‡ Department of Natural Drugs, Faculty of Pharmacy, Masaryk University, Brno 61200, Czechia; § Department of Plant Anatomy, Institute of Biology, Eötvös Loránd University, Pázmány Péter sétány 1/C, Budapest 1117, Hungary; ∥ Institute of Biology, Doctoral School of Biology, 54616 ELTE Eötvös Loránd University, Pázmány Péter sétány 1/C, Budapest 1117, Hungary; ⊥ HUN-REN-ELTE Research Group of Peptide Chemistry, Hungarian Research Network, ELTE Eötvös Loránd University, Pázmány Péter sétány 1/A, Budapest 1117, Hungary; # Department of Pharmaceutical Chemistry, Semmelweis University, Hőgyes Endre u. 9, Budapest 1092, Hungary; ∇ Center for Pharmacology and Drug Research & Development, Semmelweis University, Budapest 1085, Hungary; ○ Department of Genetics, Cell- and Immunobiology Cell- and Immunobiology, Semmelweis University, Nagyvárad tér 4, Budapest 1089, Hungary; ◆ Integrative Health and Environmental Analysis Research Laboratory, Department of Analytical Chemistry, Institute of Chemistry, Eötvös Loránd University, Budapest 1117, Hungary

## Abstract

Novel compounds, including four isomeric monomalonyl-dicaffeoylquinic acids (**4**–**7**), one dimalonyl-dicaffeoylquinic acid (**9**), and one flavonoid-dimalonyl-glucoside (**8**), along with three known flavonoid-monomalonyl-glucosides (**1**–**3**), were discovered in closely related edible Apiaceae plants: *Anthriscus cerefolium*, *Anthriscus sylvestris*, and *Chaerophyllum bulbosum*. Their structures were elucidated through comprehensive HPLC-UV-HR-MS/MS and NMR analyses, and isomeric malonyl-dicaffeoylquinic acids (**4**–**7**) were differentiated based on HPLC-MS/MS fragmentation characteristics. The study confirmed organ- and vegetation phase-specific accumulation, identifying optimal plant tissues for targeted isolation using a one-step preparative HPLC method. Malonyl-dicaffeoylquinic acids **4** and **9** exhibited significant cytotoxicity to nontumorous Vero E6 cells *in vitro* (IC_50_ < 10 μM). At the same time, the isolated compounds displayed structure-specific DPPH radical scavenging activity, underscoring their dual biological relevance.

## Graphical abstract

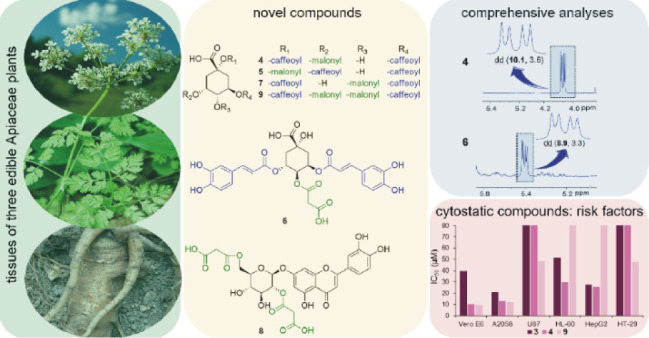

## Introduction

*Anthriscus cerefolium* (L.) Hoffm., *Anthriscus sylvestris* (L.) Hoffm., and *Chaerophyllum bulbosum* (L.) are closely related herbaceous edible plants of the Apiaceae family.^1–3^ They are commonly found in temperate regions of Europe and Asia, but *A. sylvestris* and *A. cerefolium* have also naturalized in North America.^4–6^ In addition to their common use in salads, *A. cerefolium* (French parsley) is used as a seasoning, the stems of *A. sylvestris* (cow parsley) are pickled, and the thickened taproots (tubers) of *C. bulbosum* (tuberous-rooted chervil) are harvested as a root vegetable.^7–9^ *A. cerefolium* also has significance in traditional medicine, exhibiting anti-inflammatory, hypotensive, and diuretic effects.^10,11^ Due to these valuable properties, *A. cerefolium* and *C. bulbosum* are also cultivated.^10,12^

Caffeoylquinic acids (CQAs), including malonyl-1,4-*O*-dicaffeoylquinic acid, malonyl-1,5-*O*-dicaffeoylquinic acid, and malonyl-4,5-*O*-dicaffeoylquinic acid, were tentatively identified in the aerial parts of *A. cerefolium* using high-performance liquid chromatography (HPLC) combined with high-resolution tandem mass spectrometry (HR-MS/MS).^13^ These compounds share a quinic acid core (1,3,4,5-tetrahydroxycyclohexanecarboxylic acid) esterified with one malonic acid and two caffeic acid moieties. Esterification of the hydroxyl groups at positions 1, 3, 4, and 5 of quinic acid with caffeic or malonic acid enables the formation of up to 12 regioisomeric malonyl-dicaffeoylquinic acids (MDiCQAs). Furthermore, due to the chiral nature of the carbon atoms bearing hydroxyl groups, quinic acid can form epimers, which lead to additional stereoisomeric MDiCQAs.^14^ This structural complexity poses a significant challenge for their accurate identification. Common flavonoids, cinnamic acid derivatives, and caffeoylquinic acid (chlorogenic acid) have been identified in *A. sylvestris* and *C. bulbosum*, along with demonstrating antioxidant activity in their extracts.^15–17^ *A. sylvestris* has also been found to contain lignans with *in vitro* cytostatic activity, raising safety concerns regarding its use as a culinary plant. In our earlier study on cytostatic lignans from this species, HPLC-HR-MS analysis optimized for apolar lignans revealed additional highly polar compounds. The LC-MS data suggest that these compounds could be MDiCQAs.^18^ Based on literature data on MDiCQAs in *A. cerefolium* and our preliminary findings on these compounds in *A. sylvestris*, we hypothesized that the closely related *C. bulbosum* may also contain them.^13,18^ Identifying metabolites in various plant tissues is crucial for culinary and medicinal applications. However, data on the polar metabolites of these three plants remain limited. Given the close phylogenetic relationship among these three Apiaceae species, which suggests the potential to produce similar metabolites, our research aimed to 1) identify the polar metabolites, including MDiCQAs, in various organs of wild-grown *A. cerefolium*, *A. sylvestris*, and *C. bulbosum*, collected at the beginning of the vegetation cycle and during the flowering stage; 2) identify optimal tissues with relatively high levels of selected compounds, facilitating their isolation through a one-step preparative HPLC method; 3) unambiguously determine the structures of the isolated compounds using nuclear magnetic resonance (NMR) spectroscopy, with particular attention to the regioisomers and stereoisomers of MDiCQAs; 4) analyze the MS/MS fragment ion profiles of compounds to identify diagnostic ions suitable for distinguishing isomeric MDiCQAs; and 5) determine the *in vitro* antioxidant and cytostatic activities of the isolated compounds, to assess their potential for further drug development.

## Materials and Methods

### General Experimental Procedures

The specific absorbance and λ_max_ values, along with optical rotations, were determined in MeOH at 25 °C using a Jasco V-550 UV/vis spectrophotometer and a Jasco-P-200 polarimeter (JASCO International Co., Ltd., Tokyo, Japan), respectively.

### Analytical HPLC with UV and High-Resolution Orbitrap Mass Spectrometry; Preparative HPLC with UV Detection

Reversed-phase (RP) HPLC methods were used for analysis and isolation, utilizing analytical and preparative Dionex Ultimate 3000 HPLC systems. Details on the analysis, quantitation, and isolation of the compounds are provided in the Supporting Information.

### NMR Analysis

NMR spectra of isolated compounds were recorded with a Bruker Avance III HD 500 (500/125 MHz) spectrometer at 295 K while the band selective-HMBC spectrum of compound **6** was recorded at 328 K. A detailed description of the analysis can be found in the Supporting Information.

### Materials and Reagents

Details of the materials and reagents used to analyze and isolate plant metabolites, as well as to test their antioxidant and cytostatic activities, are provided in the Supporting Information.

### Plant Material

Plant samples were collected from various locations in Hungary (details on plant collection and sample preparation are provided in the Supporting Information).

### Preparation of Plant Extracts for Analysis and Isolation

Plant tissue extracts prepared with methanol were used for analyses and isolations, as detailed in the Supporting Information.

### Antioxidant Activity Tests

A DPPH radical scavenging assay was conducted following a previously published protocol, as detailed in the Supporting Information.^19^

### Determination of the In Vitro Cytostatic Effects of Compounds

The cytostatic effects of the compounds were evaluated *in vitro* on various cancer cells and noncancerous Vero E6 cells using the Alamar Blue viability assay, as detailed in the Supporting Information.^20^

### Structural Characterization of Compounds 4–9

#### 1,5-Dicaffeoyl-3-malonylquinic Acid (4)

Light yellow amorphous solid; [α]^25^_D_ = −110.2 (*c* 1.0, MeOH); UV (MeOH) λ_max_ (log ε) 335 (4.49); for ^1^H NMR and ^13^C NMR data, see Table 1; HRESIMS (negative) *m*/*z* 601.1198 [M–H]^−^ (calcd for 601.1199, C_28_H_25_O_15_); for all HR-Orbitrap-MS data, see Supplementary Table S1.

**Table 1:** ^1^H NMR and ^13^C Data of the Malonyl-Dicaffeoylquinic Acids (**4**–**7**, **9**) Recorded in DMSO-*d*_6_ at 500/125 MHz (δ in ppm, *J* in Hz)

Compounds										
	**4**		**5**		**6**		**7**		**9**	
Position	δ_H_	δ_C_	δ_H_	δ_C_	δ_H_	δ_C_	δ_H_	δ_C_	δ_H_	δ_C_
1	-	78.7	-	78.8^b^	-	72.3	-	78.9	-	78.2
2	2.41, m (ov)	31.7	2.42, m (ov)	31.6	2.21, m 2.00, m	35.1	2.39, m (ov) 2.31, m	33.7^c^	2.53, m (ov)	31.4
3	5.32, q (4.1)	71.7	5.34, q (3.8)	70.7	5.32, br s	67.3	4.27, q (3.4)	64.8^c^	5.47, q (3.8)	68.5
4	3.87, dd (8.8, 3.4)	68.7	3.87, dd (8.3, 2.5)	68.7	5.15, dd (7.3, 3.3)	70.3	4.93, dd (9.1, 3.4)	74.4^c^	5.25, dd (9.6, 3.8)	71.2
5	5.19, td (9.1, 4.1)	69.8	5.22, td (8.6, 3.9)	69.8	5.39, dt (7.2, 3.7)	67.8	5.46, td (9.3, 4.3)	66.3^c^	5.41, td (9.8, 4.3)	66.5
6	2.44, m (ov) 1.95, dd (13.1, 9.9)	35.6	2.38, m (ov) 1.95, br t (11.1)	35.6	2.28, dd (13.2, 3.2) 1.95, dd (13.5, 7.6)	35.1	2.40, m (ov) 2.02, dd (13.0, 10.5)	35.9^c^	2.52, m (ov) 2.12, dd (13.3, 10.8)	35.8
COOH	-	172.0	-	171.6	-	175.2	-	n.d.	-	171.5
	**1-caffeoyl**		**3-caffeoyl**		**3-caffeoyl**		**1-caffeoyl**		**1-caffeoyl**	
1’	-	165.2	-	166.1	-	165.3	-	165.2	-	165.2
2’	6.28, d (15.9)	113.8	6.29, d (15.9)	114.3	6.15, d (15.8)	113.5	6.22, d (15.8)	114.2	6.32, d (15.8)	113.6
3′	7.49, d (15.9)	146.0	7.47, d (15.9)	145.3/145.2	7.45, d (15.8)	145.64	7.47, d (15.8)	145.62/145.58	7.51, d (15.8)	146.2
4’	-	125.4	-	125.6/125.5	-	125.47/125.53	-	125.4	-	125.43/125.36
5′	7.07, d (2.0)	115.1	7.06, d (2.0)/7.05, d (2.0)	115.0/114.9	7.06, d (1.9)/7.04, d (1.9)	114.9	7.05, m	115.0/114.9	7.09, d (1.9)	115.2
6’	-	145.6	-	145.55/145.58	-	145.55	-	145.62/145.58	-	145.6
7’	-	148.6	-	148.4/148.3	-	148.4/148.5	-	148.50/148.48	-	148.7/148.6
8’	6.77, d (8.2)	115.8	6.77, d (8.1)/6.76, d (8.1)	115.8	6.77, d (8.1)/ 6.76, d (8.1)	115.74/115.77	6.78, d (8.0)/6.76, d (8.0)	115.8	6.78, d (8.1)/6.76, d (8.1)	115.81/115.76
9’	7.03, dd (8.2, 2.0)	121.4	7.02, d (2.0)/7.00, d (2.0)	121.4/121.3	7.00, dd (8.1, 1.9)	121.5	7.00, m (ov)	121.2	7.05, m (ov)	121.5
	**5-caffeoyl**		**5-caffeoyl**		**5-caffeoyl**		**5-caffeoyl**		**5-caffeoyl**	
1″	-	166.0	-	165.8	-	165.9	-	165.8	-	165.7
2″	6.23, d (15.9)	113.9	6.22, d (15.9)	114.0	6.20, d (15.8)	114.0	6.18, d (15.8)	113.6	6.20, d (15.8)	113.3
3″	7.50, d (15.9)	145.4	7.49, d (15.9)	145.3/145.2	7.49, d (15.8)	145.4	7.46, d (15.8)	145.62/145.58	7.47, d (15.8)	145.9
4″	-	125.5	-	125.6/125.5	-	125.47/125.53	-	125.4	-	125.43/125.36
5″	7.06, d (2.0)	114.9	7.06, d (2.0)/7.05, d (2.0)	115.0/114.9	7.06, d (1.9)/ 7.04, d (1.9)	114.9	7.05, m	115.0/114.9	7.04, d (1.9)	115.0
6″	-	145.6	-	145.55/145.58	-	145.55	-	145.62/145.58	-	145.6
7″	-	148.5	-	148.4/148.3	-	148.4/148.5	-	148.50/148.48	-	148.7/148.6
8″	6.78, dd (8.2)	115.8	6.77, d (8.1)/6.76, d (8.1)	115.8	6.77, d (8.1)/ 6.76, d (8.1)	115.74/115.77	6.78, d (8.0)/6.76, d (8.0)	115.8	6.78, d (8.1)/6.76, d (8.1)	115.81/115.76
9″	7.01, dd (8.2, 2.0)	121.4	7.02, d (2.09)/7.00, d (2.0)	121.4/121.3	7.00, dd (8.1, 1.9)	121.3	7.00, m (ov)	121.2	7.00, dd (8.1,1.9)	121.6
	**3-malonyl**		**1-malonyl**		**4-malonyl**		**4-malonyl**		**3-malonyl**	
CH_2_	3.29, d (15.9) 3.19, m (ov)^a^	41.8	3.35, d (15.9)	41.9	3.36, s	41.6	3.31, d (15.7) 3.25, d (15.7)	42.6	3.28, m (ov) 3.15, m (ov)	41.6
RCOOR’	-	166.6	-	166.2	-	166.2	-	167.1	-	166.4
RCOOH	-	167.8	-	167.3	-	167.7	-	168.2	-	167.6
									**4-malonyl**	
CH_2_	-	-	-	-	-	-	-	-	3.28, s	41.5
RCOOR″	-	-	-	-	-	-	-	-	-	166.3
RCOOH	-	-	-	-	-	-	-	-	-	167.7

a The signal overlaps with the signal of residual methanol.

b Signal deduced from the HMBC spectrum.

c Chemical shifts deduced from HSQC and HMBC spectra; n.d., not detected.

#### 3,5-Dicaffeoyl-1-malonylquinic Acid (5)

Light yellow amorphous solid; [α]^25^_D_ = −147.5 (*c* 1.0, MeOH); UV (MeOH) λ_max_ (log ε) 335 (4.49); for ^1^H NMR and ^13^C NMR data, see Table 1; HRESIMS (negative) *m*/*z* 601.1205 [M–H]^−^ (calcd for 601.1199, C_28_H_25_O_15_); for all HR-Orbitrap-MS data, see Supplementary Table S1.

#### 3,5-Dicaffeoyl-4-malonyl-epi-quinic Acid (6)

Light yellow amorphous solid; [α]^25^_D_ = −141.5 (*c* 1.0, MeOH); UV (MeOH) λ_max_ (log ε) 335 (4.49); for ^1^H NMR and ^13^C NMR data, see Table 1; HRESIMS (negative) *m*/*z* 601.1207 [M–H]^−^ (calcd for 601.1199, C_28_H_25_O_15_); for all HR-Orbitrap-MS data, see Supplementary Table S1.

#### 1,5-Dicaffeoyl-4-malonylquinic Acid (7)

Light yellow amorphous solid; [α]^25^_D_ = −114.3 (*c* 1.0, MeOH); UV (MeOH) λ_max_ (log ε) 335 (4.51); for ^1^H NMR and ^13^C NMR data, see Table 1; HRESIMS (negative) *m*/*z* 601.1201 [M–H]^−^ (calcd for 601.1199, C_28_H_25_O_15_); for all HR-Orbitrap-MS data, see Supplementary Table S1.

#### Luteolin-7*-*O*-(*2″,6″-di*-*O*-*malonyl*)-*β*-*D*-*glucoside (8)

Yellow amorphous solid; [α]^25^_D_ = −83.4 (*c* 1.0, MeOH); UV (MeOH) λ_max_ (log ε) 348 (4.25); for ^1^H NMR and ^13^C NMR data, see Table 2; HRESIMS (negative) *m*/*z* 619.0943 [M–H]^−^ (calcd for 619.0941, C_27_H_23_O_17_); for all HR-Orbitrap-MS data, see Supplementary Table S2.

**Table 2:** ^1^H and ^13^C NMR Data of the Flavonoid-Malonyl-Glucosides (**1**–**3**, **8**) Recorded in DMSO-*d*6 at 500/125 MHz (δ in ppm, *J* in Hz)^a^

Compounds								
	1		2		3		8	
Position	δ_H_	δ_C_	δ_H_	δ_C_	δ_H_	δ_C_	δ_H_	δ_C_
2	-	156.7	-	164.8	-	156.7	-	164.8
3	-	133.2	6.75, s	103.1	-	133.1	6.76, s	103.1
4	-	177.3	-	181.9	-	177.4	-	181.9
5	-	161.2	-	161.0	-	161.2	-	161.1
6	6.20, d (2.0)	98.7	6.42, d (1.9)	99.8	6.21, d (2.0)	98.8	6.38, d (2.0)	99.8
7	-	164.2	-	162.8	-	164.3	-	162.1
8	6.40, d (2.0)	93.6	6.77, d (2.0)	94.5	6.43, d (2.0)	93.7	6.72, d (2.0)	94.9
8a	-	156.4	-	157.1	-	156.4	-	156.9
4a	-	103.9	-	105.5	-	103.9	-	105.8
1’	-	121.0	-	121.3	-	120.7	-	121.3
2’	7.51, d (2.1)	116.2	7.53, br s	113.5	7.98, d (8.9)	130.8	7.51, brs	113.5
3′	-	144.9	-	146.2	6.88, d (8.9)	115.1	-	146.1
4’	-	148.6	-	150.0	-	160.1	-	150.1
5′	6.83, d (8.4)	115.1	6.87, d (8.4)	115.9	6.88, d (8.9)	115.1	6.87, d (8.4)	115.9
6’	7.49, dd (8.4, 2.2)	121.5	7.41, dd (8.4, 2.1)	118.9	7.98, d (8.9)	130.8	7.42, dd (8.4, 2.2)	119.0
1″	5.37, d (7.4)	101.1	5.07, d (7.3)	100.0	5.34, d (7.5)	101.3	5.38, d (8.1)	97.3
2″	3.24, m (ov)	73.9	3.29, m (ov)	73.1	3.20, m (ov)	74.0	4.84, dd (9.3, 8.4)	73.8
3″	3.24, m (ov)	76.2	3.32, m (ov)	76.1	3.22, m (ov)	76.1	3.58, t (9.2)	73.3
4″	3.18, m	69.5	3.17, m	69.7	3.14, m (ov)	69.5	3.31, m (ov)	69.7
5″	3.31, m (ov)	73.94	3.73, m	74.2	3.31, m (ov)	73.9	3.87, m	74.1
6″	4.19, dd (11.8, 1.8) 3.99, dd (11.9, 5.7)	63.5	4.46, d (11.2) 4.04, dd (11.9, 7.5)	64.0	4.16, dd (11.8, 1.7) 4.00, dd (11.9, 5.7)	63.4	4.46, d (11.0) 4.12, dd (11.9, 7.1)	63.8
							**2’’-malonyl**	
CH_2_	-	-	-	-	-	-	3.38, m (ov)	41.8
RCOOR’	-	-	-	-	-	-	-	166.2
RCOOH	-	-	-	-	-	-	-	167.8
**6″-malonyl**								
CH_2_	3.07, s	41.5	3.31, m (ov)	42.6	3.07, s	41.6	3.35, m (ov)	42.3
RCOOR″	-	166.8	-	167.5	-	166.8	-	167.3
RCOOH	-	167.9	-	168.0	-	167.9	-	168.0

a Note: Ov, signals in the overlapped regions of the spectra and the multiplicities could not be recognized.

#### 1,5-Dicaffeoyl-3,4-dimalonylquinic Acid (9)

Light yellow amorphous solid; [α]^25^_D_ = −135.3 (*c* 1.0, MeOH); UV (MeOH) λ_max_ (log ε) 335 (4.48); for ^1^H NMR and ^13^C NMR data, see Table 1; HRESIMS (negative) *m*/*z* 687.1205 [M–H]^−^ (calcd for 687.1203, C_31_H_27_O_18_); for all HR-Orbitrap-MS data, see Supplementary Table S1.

## Results and Discussion

### Identification of Compounds in the Extracts of *A. cerefolium*, *A. sylvestris*, and *C. bulbosum* by HPLC-UV-Orbitrap-HR-MS

Leaf and flower samples of *A. cerefolium*, *A. sylvestris*, and *C. bulbosum* were collected at the beginning of the vegetation cycle in early spring when the first leaves appeared and during the flowering stage in June. The HPLC-UV separations of extracts from *A. cerefolium*, *A. sylvestris*, and *C. bulbosum* tissues reveal nine prominent peaks corresponding to the main metabolites in the extracts (Figures 1, 2 and S1).

**Figure 1:**
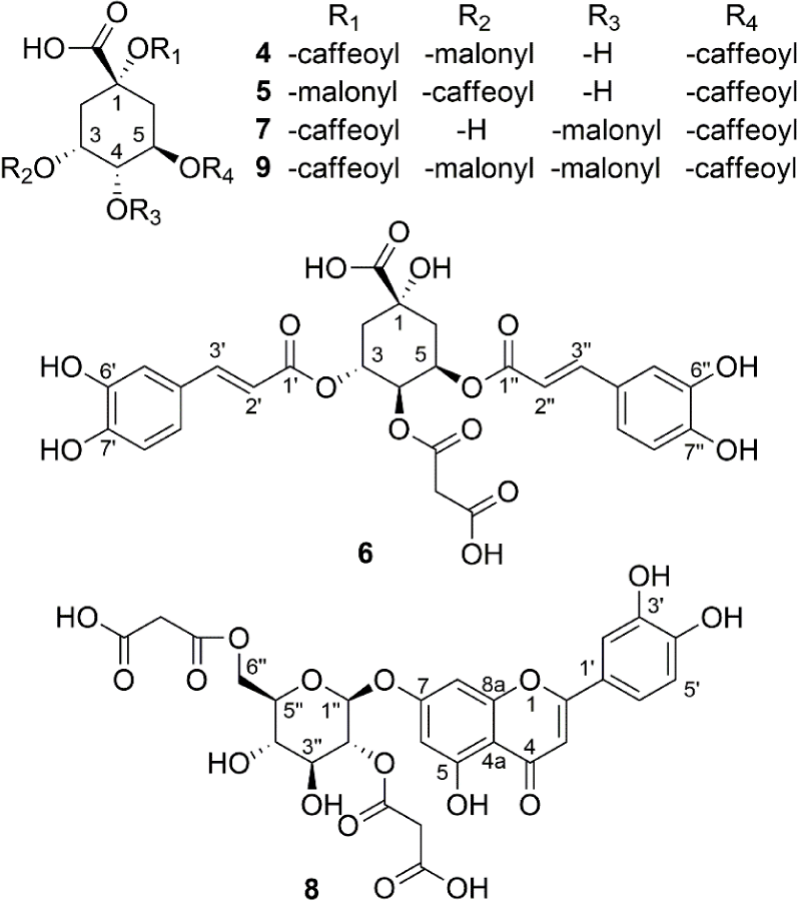
Chemical structures of newly identified compounds **4**–**9**.

**Figure 2:**
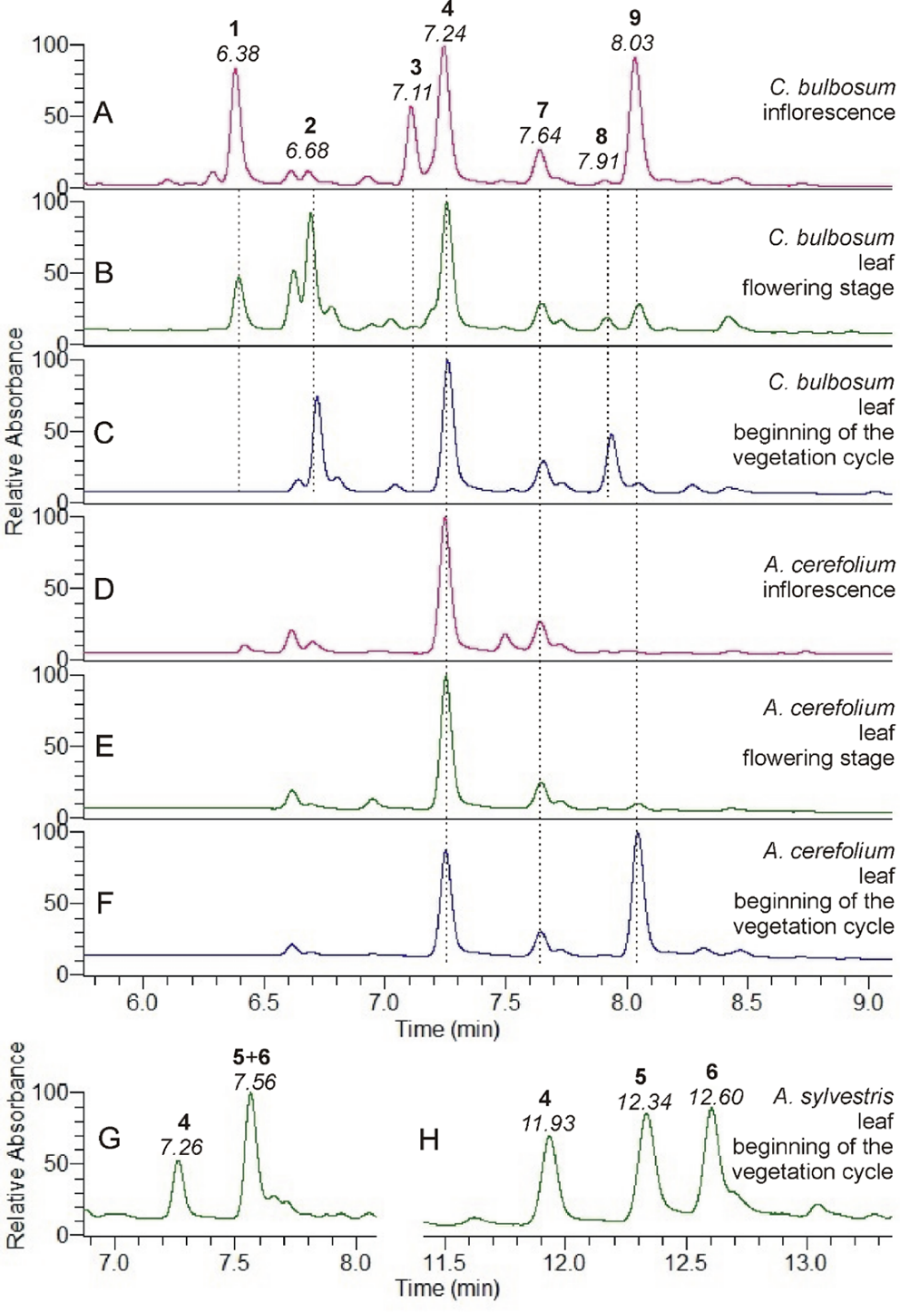
HPLC-UV (λ = 230–600 nm, total scan) analysis of the *Chaerophyllum bulbosum* (A, B, C) and *Anthriscus cerefolium* (D, E, F) extracts using gradient program 1 and the *Anthriscus sylvestris* (G, H) extract using both gradient program 1 (G) and gradient program 2 (H). The *Chaerophyllum bulbosum* and *Anthriscus cerefolium* extracts were prepared from their inflorescences (A, D) and leaves (B, C, E, F), which were collected at the flowering stage (B, E) and the beginning of the vegetation cycle (C, F). The *Anthriscus sylvestris* extract was prepared from the leaves collected at the beginning of the vegetation cycle. Peak numbers (in bold) correspond to the numbering of the identified compounds, with retention times indicated in italics.

Compounds with comparable retention times in the chromatograms of the plant extracts exhibit nearly identical HR-MS ion profiles, suggesting structural identity. Compound **4** is present in all samples, while compound **7** is detected in *A. cerefolium* and *C. bulbosum* samples. Both compounds exhibit similar UV spectra, characterized by a broad absorption band with a maximum of 280–330 nm (with a shoulder at 290 nm), characteristic of caffeic acid derivatives. Furthermore, they share the same molecular formula of C_28_H_26_O_15_ (Table S1, Figures S2 and S3). These data support their identification as MDiCQA isomers.^21,22^ The HPLC-UV chromatogram of the extract prepared from *A. sylvestris* contains an additional peak (at 7.56 min), which can also be identified as an MDiCQA isomer. The tandem mass spectra of this compound, analyzed from its appearance to the end in the chromatogram, showed varying intensity ratios of the product ions. Since this indicated that several compounds were coeluting, the HPLC gradient program was modified for their separation. Compounds **5** and **6** can be separated by applying the improved method, allowing for their HPLC-UV-HR-MS identification as two further MDiCQA isomers (Table S1, Figures S4 and S5).

Identifying compound **9** in *A. cerefolium* and *C. bulbosum* samples was particularly challenging, revealing an unexpected chemical characteristic of MDiCQAs. Like the known MDiCQA isomers, compound **9** exhibited a UV maximum in the 280–330 nm range, suggesting structural similarity to MDiCQAs. The HR-MS spectra of compound **9**, obtained under negative ionization, exhibited ions at *m*/*z* 683.09, *m*/*z* 685.10, and *m*/*z* 687.12 (Table S1 and Figure S6). The ion at *m*/*z* 687.12 corresponded to the molecular formula of C_31_H_27_O_18_, consistent with a deprotonated isomer of dimalonyl-dicaffeoylquinic acid (DiMDiCQA). However, additional ions at *m*/*z* 683.09 and *m*/*z* 685.10 indicated the presence of a doubly oxidized and a singly oxidized deprotonated DiMDiCQA, respectively. The HR-MS analysis of compounds **4–7** also revealed oxidized species. In addition to the predominant deprotonated ion at *m*/*z* 601.12, additional ions at *m*/*z* 597.10 (doubly oxidized) and *m*/*z* 599.09 (singly oxidized) were detected, with relative intensities of 5–10% and 30–90%, respectively (Table S1, Figures S2–S5). We assumed that the pH of the HPLC eluents influenced the formation of oxidized derivatives. To investigate this, HPLC-UV-HR-MS analyses were conducted using three different eluent conditions: (1) 0.1% v/v formic acid in water (the original eluent A), (2) water without formic acid, and (3) 0.3% v/v formic acid in water, which were used as eluent A (all other analytical parameters remained unchanged). When water alone was used as eluent A, the predominant ion in the HR-MS spectrum of compound **4** was *m*/*z* 597.09 (doubly oxidized), followed by *m*/*z* 599.10 (singly oxidized ∼30% relative intensity) and *m*/*z* 601.12 (deprotonated, ∼10%). Similarly, for compound **9**, only traces of the deprotonated molecule (*m*/*z* 687.12) and its singly oxidized form (*m*/*z* 685.10) were observed, while the doubly oxidized species (*m*/*z* 683.09) dominated (Figures S7 and S8). In contrast, increasing the formic acid concentration to 0.3% v/v significantly reduced the formation of oxidized ions compared to both the 0.1% v/v formic acid and water-only conditions (Figures S2–S6). The presence of oxidized ions at 2 and 4 Da lower than the intact species was also confirmed in positive ionization mode, indicating that oxidation occurs independently of ionization polarity (Figures S2–S6).

Previous HPLC-MS studies on catechol-containing molecules have demonstrated that oxidation in the ion source involves the loss of two hydrogen atoms from the ortho-hydroxy groups of the catechol moiety, resulting in ions 2 Da lighter than the nonoxidized species.^23^ Given the two catechol units in MDiCQAs, we propose that one or both undergo oxidation, generating singly (*m*/*z* −2) and doubly (*m*/*z* −4) oxidized ions.

Increasing the formic acid concentration to 0.3% v/v in HPLC-UV-HR-MS analysis of MDiCQAs improves spectral clarity by minimizing oxidized ion formation, allowing for more accurate characterization of these compounds.

To the best of our knowledge, this is the first report to describe the impact of eluent pH on ion-source oxidation of catechol-containing compounds.

In addition to the MDiCQAs (**4**–**7**, **9**), extracts of *C. bulbosum* contained four compounds (**1**, **2**, **3**, **8**) exhibiting a broad UV absorption band between 340 and 360 nm, characteristic of flavonoids. The molecular formulas determined from the HR-MS data indicate that compound **1** is the malonyl-glucoside of quercetin, while compounds **2** and **3** are malonyl-glucosides of either kaempferol or luteolin (**2**, **3**) (Table S2). Comparing the molecular formula of compound **8** (C_27_H_24_O_17_) with that of compounds **2** and **3** (C_24_H_22_O_14_) suggests that **8** contains an additional malonyl unit relative to **2** or **3**, indicating that **8** is the dimalonyl-glucoside of either kaempferol or luteolin (Table S2). In the HR-MS spectrum of compound **8**, obtained under negative ionization, the most intense ion at *m*/*z* 619.09 corresponded to the deprotonated molecule. An unexpected ion was also observed at *m*/*z* 617.08 (Figure S9 and Table S2). Similar to MDiCQAs, the 2 Da mass difference between these ions can be attributed to the oxidation of a catechol unit in the ion source. Since luteolin contains a catechol group, its presence in compound **8** can be assumed.

### Mass Fragmentation Study of Compounds 1–9 by HPLC-HR-MS/MS

The mass fragmentation spectra of MDiCQAs **4**–**7**, obtained in negative ionization mode using various collision-induced dissociation (CID) energies from the deprotonated molecules (*m*/*z* 601), show identical fragment ions (Table S3, Figures S10–S13). Ions at *m*/*z* 515, *m*/*z* 439, and *m*/*z* 353 correspond to deprotonated dicaffeoylquinic acid, malonyl-caffeoylquinic acid, and caffeoylquinic acid fragments. These fragments are generated by the loss of a malonyl group and a caffeoyl group and their sequential elimination from compounds **4**–**7**. The fragments generated by the loss of a malonyl group and a caffeoyl group can also be detected in the mass fragmentation spectra of deprotonated DiMDiCQA **9** (*m*/*z* 687) at *m*/*z* 601 and *m*/*z* 525 (Table S4 and Figure S14). The mass fragmentation spectra of all MDiCQAs (**4**–**7** and **9**) exhibit identical ions at *m*/*z* 557, *m*/*z* 439, *m*/*z* 395, *m*/*z* 377, and *m*/*z* 233. In compounds **4**–**7**, these ions are generated by eliminating CO_2_, a caffeoyl group, a caffeoyl group + CO_2_, a caffeoyl group + CO_2_ + H_2_O, and two caffeoyl groups + CO_2_, respectively. In compound **9**, the fragmentation pattern follows the same trend but occurs only after the initial loss of a malonyl group. The deprotonated quinic acid (*m*/*z* 191) and caffeic acid (*m*/*z* 179) are also observed in the mass fragmentation spectra of all MDiCQAs, along with their further decomposition products (Tables S3, S4, Figures S10–S14). The mass fragmentation spectra of flavonoid-malonyl-glucosides (FMGls, **1**, **2**, **3**, and **8**) were generated by negative and positive ionization modes using various CID energies from the deprotonated and protonated molecules (Table S5). The fragment ions at *m*/*z* 463, *m*/*z* 447, and *m*/*z* 533 are generated through negative ionization from the deprotonated molecules of compounds **1** (*m*/*z* 549), **2** and **3** (both at *m*/*z* 533), and **8** (*m*/*z* 619), respectively. These fragment ions could be formed by eliminating one malonyl group from each deprotonated flavonoid. Considering the differences between the *m*/*z* values of the demalonylated fragment ions and the corresponding flavonoid aglycones generated as the final products of fragmentation processes, the presence of a glucosyl unit in **1**, **2**, **3**, and a malonyl-glucosyl unit in **8** can be confirmed. The aglycones of these flavonoids were detected in both ionization modes, positive and negative, as protonated and deprotonated molecules. This allowed for the calculation of molecular formulas: C_15_H_10_O_7_ for compound **1**, which corresponds to quercetin, and C_15_H_10_O_6_ for compounds **2**, **3**, and **8**, which correspond to either kaempferol or luteolin.

The results of the mass fragmentation study of compounds **1**–**9** confirmed their structural units, such as quinic acid, caffeic acid, and malonic acid in compounds **4**–**7** and **9**, as well as flavonoid aglycones, glucose, and malonic acid in compounds **1**, **2**, **3**, and **8**. The exact structures of compounds **1**–**9** were determined by NMR analyses.

### Identification of Compounds Using NMR

Structural characterization was based on 1D (^1^H, ^13^C) and 2D (^1^H–^1^H COSY, ^1^H–^1^H ROESY, ^1^H–^13^C HSQC, and ^1^H–^13^C HMBC) experiments (Tables 1, 2, Figures S15–87). The ^13^C NMR spectrum of MDiCQA **4** displayed 28 distinct carbon resonances. Assisted by the ^1^H and HSQC spectra, these were classified as three methylene groups, 13 methine carbons, and 12 nonprotonated carbon atoms, including five carbonyl carbons, six sp^2^ carbons, and one oxygenated tertiary carbon. The ^1^H NMR exhibited signals for 19 protons, suggesting that the remaining seven protons are part of hydroxyl and carboxyl groups. Six aromatic proton signals at δ_H_ 7.07 (1H, d, *J* = 2.0 Hz, H-5′), 7.06 (1H, d, *J* = 2.0 Hz, H-5″), 7.03 (1H, dd, *J* = 8.2, 2.0 Hz, H-9’), 7.01 (1H, dd, *J* = 8.2, 2.0 Hz, H-9″), 6.78 (1H, dd, *J* = 8.2 Hz, H-8″), 6.77 (1H, d, *J* = 8.2 Hz, H-8’) displayed as two ABX systems, indicative of two 1,2,4-trisubstituted benzene units.

Additionally, four doublets at δ_H_ 7.50 (1H, d, *J* = 15.9 Hz, H-3″), 7.49 (1H, d, *J* = 15.9 Hz, H-3′), 6.28 (1H, d, *J* = 15.9 Hz, H-2’), 6.23 (1H, d, *J* = 15.9 Hz, H-2″) revealed two vinylene groups with *trans*-oriented protons (Table 1, Figures S38–S45). These structural units, supported by the HMBC correlations H-3′/C-1’, H-3′/C-4’, H-3′/C-5′, H-3′/C-9’ and H-3′/C-1″, H-3″/C-4″, H-3″/C-5″, H-3″/C-9″, were identified as two caffeoyl moieties (Figure S15). A pair of sp^3^ methylene protons at δ_H_ 3.29 (1H, d, *J* = 15.9 Hz) and 3.19 (1H, m (ov)), along with their HMBC correlations with two carbonyl carbons at δ_C_ 166.6 (RCOOR’) and 167.8 (RCOOH), indicated the presence of a malonyl moiety. The remaining seven protons, including three oxymethine protons at δ_H_ 5.32 (1H, q, *J* = 4.1 Hz, H-3), 5.19 (1H, td, *J* = 9.1, 4.1 Hz, H-5), 3.87 (1H, dd, *J* = 8.8, 3.4 Hz, H-4) along with four sp^3^ methylene protons at δ_H_ 2.44 (1H, m (ov), H-6), 2.41 (2H, m (ov), H-2), 1.95 (1H, dd, *J* = 13.1, 9.9 Hz, H-6), were assigned to an esterified 1,3,4,5-tetrahydroxycyclohexanecarboxylic acid based on COSY cross-peaks, defining an adjacent proton sequence of −CH_2_–CH–CH–CH–CH_2_–, and the HMBC correlations, H-3/C-1, H-5/C-1 and H-6/COOH. The relative configuration of 1,3,4,5-tetrahydroxycyclohexanecarboxylic acid moiety was determined based on an analysis of signal multiplicities and spin–spin coupling constants. The large *trans*-diaxial coupling constant ^3^ *J*_4/5_ = 8.8 Hz indicated H-4 to occupy an axial position. This observation aligns well with previous reports on dicaffeoylquinic acids, which predominantly adopt a chairlike conformation with the conformational equilibrium in DMSO-*d*_6_ favoring the conformer where both H-4 and H-5 are in axial positions.^24–26^ Consequently, the apparent quartet at δ_H_ 5.32 and the apparent triplet of doublets at δ_H_ 5.19 were further attributed to H-3 (equatorial) and H-5 (axial), respectively. Based on these findings, the 1,3,4,5-tetrahydroxycyclohexanecarboxylic acid moiety was identified as a quinic acid unit. To confirm these assignments, ^1^H NMR and COSY spectra were recorded in D_2_O (Figure S46). The markedly increased *trans*-diaxial coupling constant ^3^ *J*_4/5_ = 10.1 Hz indicated a shift in the conformational equilibrium toward the conformer in which both H-4 and H-5 adopt axial positions (Figure 3), as previously reported.^26,27^

**Figure 3:**
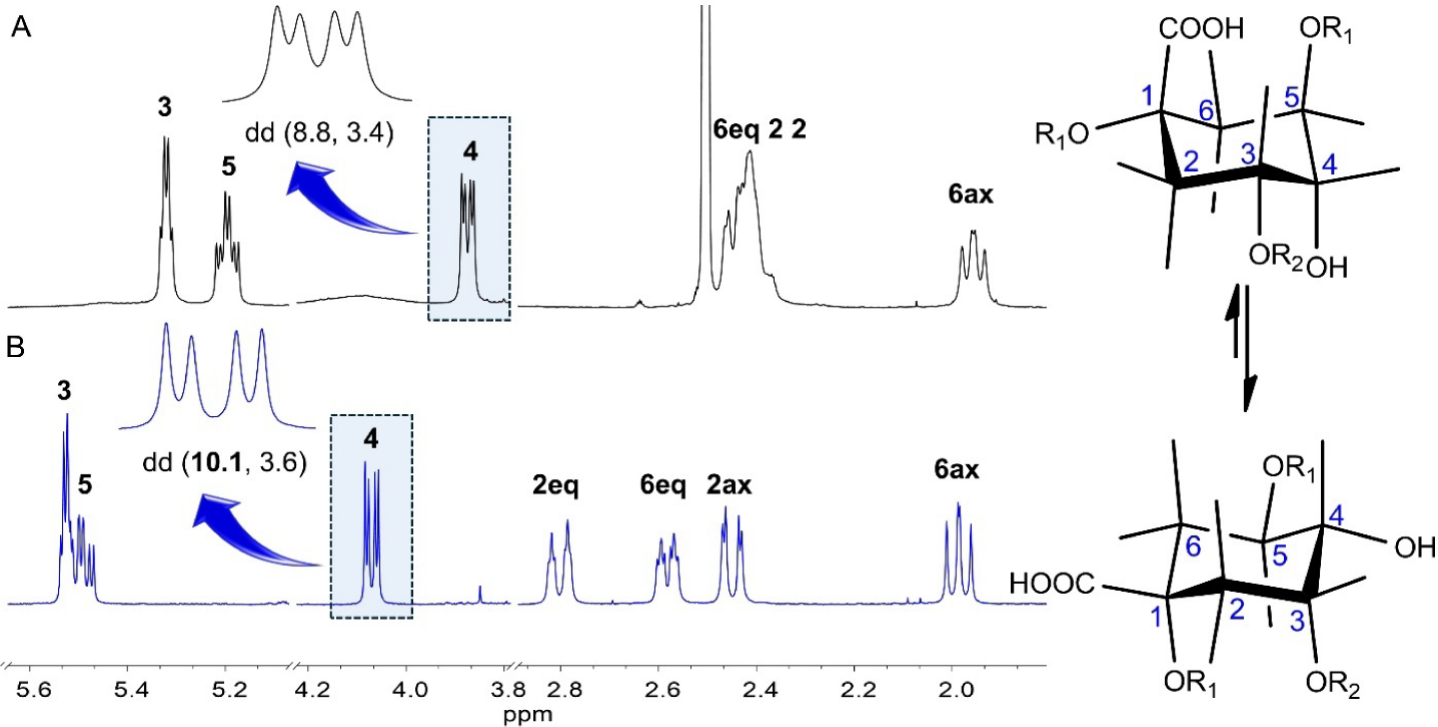
Spin–spin coupling constant analysis of diagnostic proton (H-4) for compound **4** in DMSO-*d*_6_ (A) and D_2_O (B), demonstrating the conformational preferences of quinic acid moiety in both solvents (R_1_ = caffeoyl, R_2_ = malonyl).

Additional differences in the signals of the quinic acid moiety, compared to those observed in DMSO-*d*_6_, included the partial overlap of H-3 and H-5 resonances due to their merging. The resonances of H_2_-2 separated, whereas those of H_2_-6 merged, leading to a new chemical shift order: 2eq-6eq-2ax-6ax (compared to 6eq-2­(ov)-6ax in DMSO-*d*_6_). Moreover, the methylene protons H_2_-2 and H_2_-6 appeared as separated resonances, and a W-type coupling, indicative of a highly coplanar arrangement of H-2eq/C-2/C-1/C-6/H-6eq, was observed. The attachment of caffeoyl and malonyl units at quinic acid moiety was deduced from the HMBC correlations and deshielded resonances of two oxymethine protons. A downfield shifted H-3 showed an HMBC correlation with the carbonyl carbon at δ_C_ 166.6 (RCOOR’), confirming the presence of a malonyl unit at position 3. Similarly, H-5 exhibited a downfield shift and an HMBC correlation with a carbonyl carbon at δ_C_ 166.0 and implied the attachment of a caffeoyl unit at position 5. The remaining caffeoyl unit was placed at position 1 due to the lack of correlation with a carbonyl carbon at δ_C_ 165.2. Thus, MDiCQA **4** was identified as 1,5-dicaffeoyl-3-malonylquinic acid (Figure 1).

The ^1^H NMR analysis of compound **7** revealed the presence of two caffeoyl, one malonyl and one quinic acid moiety. Minor chemical shift variations in the of H-3, H-4, and H-5 suggested that compound **7** is a positional isomer of MDiCQA **4**. In contrast to compound **4**, the upfield shift of H-3, the downfield shift of H-4 and the HMBC correlations of both malonyl methylene protons and H-4 with a carbonyl carbon at δ_C_ 167.1 established the malonyl unit at position 4 (Table 1, and Figures S64–S71). Thus, MDiCQA **7** was identified as 1,5-dicaffeoyl-4-malonylquinic acid (Figure 1).

The ^1^H NMR spectrum of compound **5** displaying resonances corresponding to two caffeoyl, one malonyl, and one quinic acid unit, clearly indicated that it is a positional isomer of MDiCQAs **4** and **7** (Table 1, and Figures S47–S54). The HMBC correlations of H-3 with carbonyl carbons at δ_C_ 166.1 and H-5 with that at δ_C_ 165.8, respectively, placed the caffeoyl moieties at positions 3 and 5 of the quinic acid core. The downfield shift of H-3 and H-5 also supported this assignment. Additionally, the absence of HMBC connectivity between quinic acid protons and the carbonyl carbon at δ_C_ 166.2 confirmed the malonyl group to be located at position 1 of the quinic acid unit. The MDiCQA **5** was therefore identified as 3,5-dicaffeoyl-1-malonylquinic acid (Figure 1).

Compared to MDiCQAs **4**, **7**, and **5**, the presence of additional signals from three carbon atoms and two methylene protons in the NMR spectra of compound **9** confirms the presence of two malonyl groups in its structure (Table 1 and Figures S80–S87). The positions of the two malonyl and two caffeoyl moieties in DiMDiCQA **9** were determined based on HMBC correlations, leading to its identification as 1,5-dicaffeoyl-3,4-dimalonylquinic acid (Figure 1).

The seven resonances corresponding to the 1,3,4,5-tetrahydroxycyclohexanecarboxylic acid core of MDiCQA **6** exhibited differences in multiplicities and spin–spin coupling constants compared to MDiCQA **4**, suggesting a different relative configuration of this structural unit (Table 1, Figures S55–S62). The decreased ^3^ *J* value (7.3 Hz) indicated the presence of an *epi*-quinic moiety^28–30^ in compound **6**. It implied that the molecule exists as an equilibrium of various conformers, predominantly chairlike conformers, in DMSO-*d*_6_.^26^ Considering the spin–spin coupling constant values calculated for both conformers of 3,5-dicaffeoyl-*epi*-quinic acid,^26^ it was inferred that two predominant chair conformers of compound **6** exist in approximately a 50:50 ratio in DMSO-*d*_6_. This equilibrium rationalizes the observed doublet of triplets at δ_H_ 5.39 (1H, dt, *J* = 7.2, 3.7 Hz, H-5) and the apparent broad singlet at δ_H_ 5.32 (1H, br s, H-3) assigned to H-5 and H-3, respectively. Further analysis of the *epi*-quinic acid unit was performed in D_2_O (Figure S63). A conformational shift toward the conformer with H-3 and H-4 in axial positions was evident from the increase in the ^3^ *J*_3/4_ value (8.9 Hz) and altered multiplicities of the H-3 and H-5 signals (Figure 4). Specifically, H-3 appeared as a multiplet at δ_H_ 5.66 (1H, m (ov)), while H-5 was observed as a quartet at δ_H_ 5.69 (1H, q, *J* = 3.7 Hz). A comparison of the ^1^H NMR spectra in D_2_O and DMSO-*d*_6_ further revealed that the H_2_-2 and H_2_-6 resonances maintained their ordering (H-6eq-2­(ov)-H-6ax), though H_2_-2 resonances merged into overlapping signals in D_2_O. The HMBC cross-peaks facilitated the assignment of acyl groups to the *epi*-quinic acid moiety. The HMBC correlations between H-3 and the carbonyl carbon at δ_C_ 165.3 and H-5 and the carbonyl carbon at δ_C_ 165.9 unambiguously confirmed the placement of the caffeoyl moieties at positions 3 and 5. The malonyl unit was assigned to C-4 based on HMBC correlations between H-4, malonyl methylene protons, and the carbonyl carbon at δ_C_ 166.2. Thus, MDiCQA **6** was identified as 3,5-dicaffeoyl-4-malonyl-*epi*-quinic acid (Figure 1).

**Figure 4:**
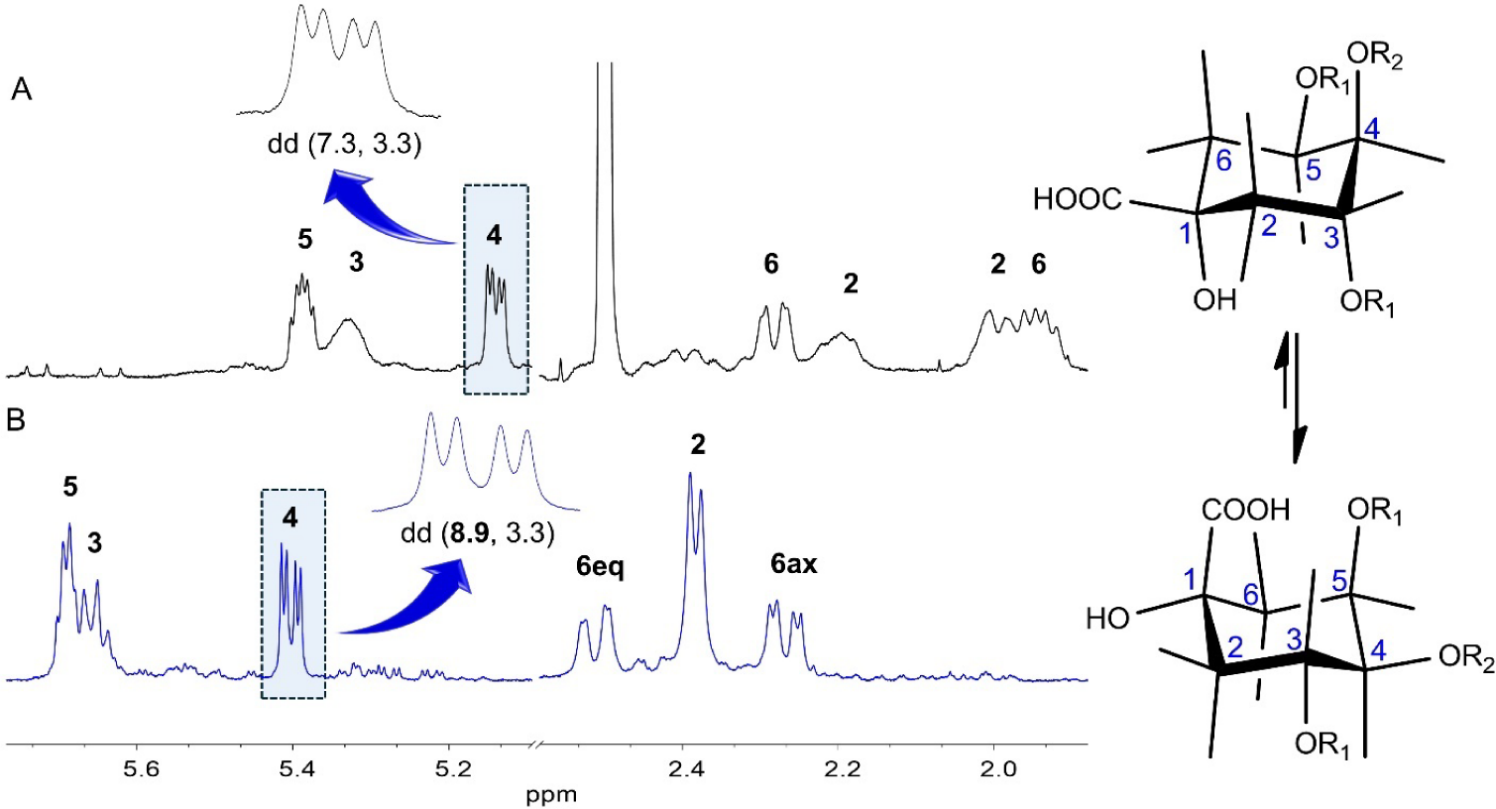
Spin–spin coupling constant analysis of diagnostic proton (H-4) for compound **6** in DMSO-*d*_6_ (A) and D_2_O (B), demonstrating the conformational preferences of *epi*-quinic acid moiety in both solvents (R_1_ = caffeoyl, R_2_ = malonyl).

A comprehensive NMR analysis of the isolated compounds in both DMSO-*d*_6_ and D_2_O, along with a comparison to the literature, revealed that the H-4 signal serves as a key indicator of the relative configuration of the 1,3,4,5-tetrahydroxycyclohexanecarboxylic acid moiety and may be considered diagnostic. A large *trans*-diaxial coupling constant ^3^ *J*_4/5_ ≈ 9 Hz) in DMSO-*d*_6_ indicated the presence of a quinic acid moiety, whereas a smaller *J* value (*J* < 8 Hz) for H-4 revealed an *epi*-quinic acid moiety.

Despite detailed NMR analyses enabling confident assignment of relative configurations and substitution patterns in the isolated MDiCQAs, a critical limitation remains: the absolute configuration of these compounds has not yet been determined unambiguously. To our knowledge, no study has reported absolute configurations for these molecules based on single-crystal X-ray diffraction. Although circular dichroism (CD) spectroscopy has been used in earlier works to infer absolute stereochemistry, such interpretations are limited.^28,31^ In substituted quinic acid derivatives like those presented here, variations in substitution patterns and resulting exciton interactions can significantly affect CD spectral profiles, preventing reliable differentiation of stereoisomers solely based on chiroptical data. Moreover, a previous study has acknowledged that stereochemical conclusions should be interpreted with caution in the absence of crystallographic data or comparison with stereochemically pure reference compounds. While solvent-dependent coupling constants and DFT-based molecular modeling provide valuable structural insights, they are not sufficient to establish absolute configuration with full certainty.^26^

Therefore, the structural assignments presented in this study reflect a rigorous relative stereochemical interpretation but remain tentative in terms of absolute configuration. X-ray crystallography remains essential for resolving these uncertainties, and its application would enable the development of reliable correlations between stereochemistry and chiroptical properties in this class of compounds.

The FMGls **1**, **2**, and **3** were identified as quercetin-3-*O*-(6″-*O*-malonyl)-β-d-glucoside,^32,33^ luteolin-7-*O*-(6″-*O*-malonyl)-β-d-glucoside,^34^ and kaempferol-3-*O*-(6″-*O*-malonyl)-β-d-glucoside,^35,36^ based on the comparison of their NMR spectroscopic data with those found in the literature (Table 2 and Figures S16–S37).

The ^1^H and ^13^C NMR spectroscopic data indicated that FMGls **2** and **8** are closely related, both containing a luteolin-7-*O*-β-d-glucoside moiety. Unlike FMGls **2**, FMGls **8** is esterified with two malonyl units (δ_H_ 3.38; δ_C_ 167.8, 166.2, 41.8 and δ_H_ 3.35; δ_C_ 168.0, 167.3, 42.3) (Table 2 and Figures S72–S79). The HMBC cross-peaks of malonyl methylene protons at δ_H_ 3.38 and H-2″ with a carbonyl carbon at δ_C_ 166.2, and malonyl methylene protons at δ_H_ 3.35 and H-6″ with a carbonyl carbon at δ_C_ 167.3 disclosed a position of malonyl units at positions 2’“and 6”’ of β-d-glucose unit. The FMGls **8** was identified as luteolin-7-*O*-(2’“,6”’-di-*O*-malonyl)-β-d-glucoside (Figure 1). Note: The anomeric proton of the glucosyl unit in compound **8** exhibited a coupling constant of 8.1 Hz, consistent with a β-glucosidic configuration (Table 2). The absolute configuration of the β-glucose moiety was not determined due to the limited amount of isolated material; however, it is strongly presumed to be the d-enantiomer, as plant-derived flavonoid glucosides invariably contain d-glucose.

### Discrimination of the Isomers of Malonyl-Dicaffeoylquinic Acids (4–7) Using HPLC-HR-MS/MS

The relative intensity of key fragment ions in the mass fragmentation spectra of compounds **4**–**7**, obtained in negative ionization mode with various CID energies from the deprotonated molecules (*m*/*z* 601), showed compound-specific differences (Table S3 and Figures S10–13). In the fragment ion spectra of compounds **4** and **7**, obtained with a CID energy of 25 eV, the fragment ion at *m*/*z* 233 exhibited the highest abundance, accounting for approximately 70% of the total intensity. Using the same CID energy (25 eV) for compounds **5** and **6**, the ion at *m*/*z* 395 was detected as the most intense in the fragment ion spectrum of **5**, representing nearly 35% of the total ion count, while the ion at *m*/*z* 233 was present only in trace amounts. However, in the fragment ion spectrum of compound **6**, the ions at *m*/*z* 395 and *m*/*z* 233 appeared with comparably high intensities, accounting for nearly 30% and 35% of the total intensity, respectively. Based on these findings, among the four MDiCQAs, only compounds **4** and **7** could not be distinguished using the two fragment ions, *m*/*z* 233 and *m*/*z* 395. The isomers **4** and **7** can also be distinguished based on their mass fragmentation spectra obtained at 45 eV (CID), which show notable differences in the intensities of the ion at *m*/*z* 173. In the MS/MS spectrum of compound **7**, the ion at *m*/*z* 173 exhibited the highest abundance, followed by the ion at *m*/*z* 233. They accounted for approximately 40% and 30% of the total ion intensity, respectively, corresponding to an abundance ratio of 1.3 between them (40%/30% ≈ 1.3). In contrast, in the MS/MS spectrum of compound **4**, the ion at *m*/*z* 173 exhibited significantly lower intensity than the ion at *m*/*z* 233, accounting for 8% and 30% of the total ion count, respectively. This results in an abundance ratio of only 0.3 between these ions for compound **4** (8%/30% ≈ 0.3). Considering the relative intensities of the key fragment ions *m*/*z* 173, *m*/*z* 233, and *m*/*z* 395, all four MDiCQAs can be identified by HLC-HR-MS/MS.

### Malonyl-Dicaffeoylquinic Acid (MDiCQA) and Flavonoid-Malonyl-Glucoside (FMGl) Composition in Different Tissues of *A. cerefolium*, *A. sylvestris*, and *C. bulbosum*

The compositions of MDiCQAs (**4**–**7**, **9**) and FMGls (**1**, **2**, **3**, **8**) were analyzed in the inflorescence, leaf, and root tissues of *A. cerefolium*, *A. sylvestris*, and *C. bulbosum* during the flowering stage. Additionally, the leaf and root tissues were also investigated at the beginning of the vegetation cycle in early spring (Tables S6–S8). Among the MDiCQAs, compounds **4**, **7**, and **9** were detected in all samples of *A. cerefolium* and *C. bulbosum*. Compound **4** was also present in all tissues of *A. sylvestris*, accompanied by compounds **5** and **6**. The inflorescences of *A. cerefolium* and *C. bulbosum* were identified as the richest sources of compounds **4** and **7**, with the highest concentrations recorded in *A. cerefolium* sample AC-I-FS-6 (28.6 mg/g and 4.6 mg/g, from location 6) and *C. bulbosum* sample CB-I-FS-5 (19.7 mg/g and 4.5 mg/g, from location 5). The inflorescence samples of *C. bulbosum* contained the highest levels of compound **9**, reaching a maximum value of 29.7 mg/g in sample CB-I-FS-5, which also had high amounts of compounds **4** and **7** (data are averages obtained from three and two parallel measurements of CB-I-FS-5 and AC-I-FS-6 samples, respectively). The results highlight the significance of *C. bulbosum* inflorescences in accumulating compounds **4**, **7**, and **9** and *A. cerefolium* inflorescences in accumulating compounds **4** and **7**. However, the highest amounts of compound **9** in *A. cerefolium* samples (4.8 mg/g), as well as compounds **5** and **6** in *A. sylvestris* samples (1.5 mg/g and 1.2 mg/g), were found in leaf samples collected during early spring (data are averages obtained from several samples, as listed in Tables S6–S8), emphasizing the significance of the vegetation phase in influencing compound accumulation.

The FMGls **1**, **2**, **3**, and **8** were identified in the tissues of *C. bulbosum*. The malonyl-glucosides of quercetin (**1**) and kaempferol (**3**) exhibited inflorescence-specific accumulation, with their average concentrations in inflorescence samples (11.5 mg/g and 4.3 mg/g) being nearly an order of magnitude higher than in leaf samples (1.2 mg/g and 0.07 mg/g) (average data were obtained from six inflorescence and seven leaf samples, as listed in Tables S7. In contrast, the malonyl-glucoside and dimalonyl-glucoside of luteolin (compounds **2** and **8**) reached their highest concentrations (7.3 mg/g and 3.3 mg/g) in leaf samples collected in early spring rather than in inflorescence samples (values represent averages obtained from five BVC samples, as listed in Tables S7.

Considering the organ- and vegetation phase-specific differences in the composition of MDiCQAs and FMGls, optimal tissues with relatively high levels of selected compounds were identified, allowing for their isolation through a one-step preparative HPLC method. We could isolate 1) from *C. bulbosum*, the MDiCQAs **4**, **7**, and **9** and the FMGls **1** and **3** from inflorescences, as well as the FMGls **2** and **4** from leaves collected at the beginning of the vegetation cycle; 2) from *A. cerefolium*, the MDiCQAs **4** and **7** from inflorescences and the MDiCQA **9** from leaves collected at the beginning of the vegetation cycle; 3) from *A. sylvestris*, the MDiCQAs **4**, **5**, and **6** from leaves collected at the beginning of the vegetation cycle.

### In Vitro Antioxidant and Cytostatic Effects of Malonyl-Dicaffeoylquinic Acids and Flavonoid-Malonyl-Glucosides

The antioxidant potential of isolated compounds was determined through a DPPH radical scavenging assay. The IC_50_ values of MDiCQAs **4**–**7** ranged from 12.0 ± 0.8 μM to 12.9 ± 0.3 μM, while that of DiMDiCQA **9** was 13.6 ± 0.4 μM, indicating a similar antioxidant capacity among these compounds (Figure 5). Comparing these values with the IC_50_ values of the standard compounds used—ascorbic acid (19.1 ± 1.5 μM), chlorogenic acid (20.3 ± 0.2 μM), and Trolox (18.4 ± 0.1 μM)—it can be concluded that our compounds exhibit comparable or even stronger DPPH radical scavenging activity. The DPPH radical scavenging activity of MDiCQAs is attributed to their catechol groups, which function as effective hydrogen donors due to the *ortho*-dihydroxy structure.^37^

**Figure 5:**
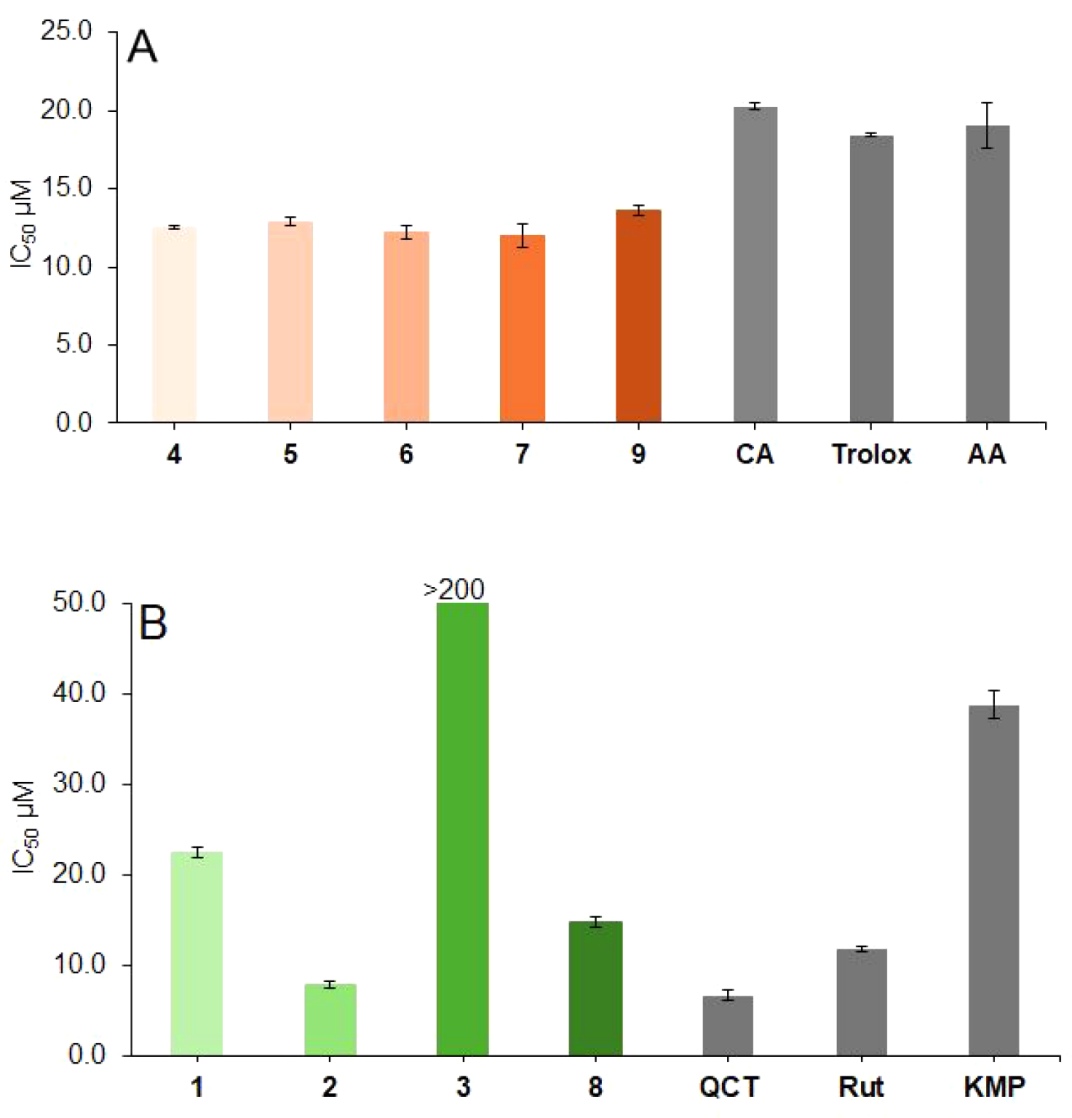
DPPH radical scavenging activity of panel A malonyl-caffeoylquinic acids **(4–7, 9)** and (B) flavonoid-malonyl-glucosides **(1–3, 8)** compared with standard antioxidants (ascorbic acid, AA; chlorogenic acid, CA; Trolox; kaempferol, KMP; rutin, Rut; and quercetin, QCT).

Three FMGls **1**, **2**, and **8** exhibited significant DPPH radical scavenging activity, as evidenced by their relatively low IC_50_ values of 22.5 ± 0.6 μM, 7.8 ± 0.4, and 14.8 ± 0.6 μM, respectively. In contrast, FMGl **3** showed much lower activity, with a considerably higher IC_50_ value of 233.9 ± 0.9 μM. Our findings for compounds **1** and **3** align with previously reported results in the literature.^32,38^ Additionally, we confirmed for the first time the significant DPPH radical scavenging activity of the previously known compound **2**, as well as that of our newly identified flavonoid, luteolin-7-*O*-(2’“,6”’-di-*O*-malonyl)-β-d-glucoside (**8**). Comparing the structures of the highly effective compounds **1**, **2**, and **8** with that of the less effective compound **3**, we observe that the B ring of the flavonoid backbone in all efficient compounds features a catechol moiety. In contrast, compound **3** has only a single hydroxyl group instead of a catechol. This finding is consistent with previous literature on the structure-antioxidant activity relationship of flavonoids, which identifies the catechol structure in the B ring as a key determinant of strong DPPH radical scavenging activity.^39^

The cytostatic effects of MDiCQAs and FMGls were evaluated *in vitro* using the Alamar Blue viability assay on five human cancer cell lines: colorectal carcinoma (HT-29), glioma (U87), hepatocellular carcinoma (HepG2), promyelocytic leukemia (HL-60), and melanoma (A2058). They were also tested on nontumorous Vero E6 cells, kidney epithelial cells from an African green monkey, to evaluate the selectivity of the compounds. Two MDiCQAs (**4** and **9**) and the FMGl **3** exhibited a moderate cytostatic effect, whereas the other isolated compounds showed no activity against the tested cells (Table 3). The two effective MDiCQAs (**4** and **9**) share a common structural feature: their central quinic acid core is substituted with caffeoyl moieties at the C-1 and C-5 positions and a malonyl group at the C-3 position. In contrast, the ineffective compound **7** has caffeoyl moieties at the same positions, but its malonyl group is attached to the C-4 position instead of C-3. Similarly, in the other ineffective MDiCQAs (**5** and **6**), the malonyl unit is also located at the C-4 position, while their caffeoyl groups are positioned at C-3 and C-5. These findings suggest that the position of the malonyl group at C-3 and caffeoyl moieties at C-1 and C-5 is crucial for the cytostatic effects of MDiCQAs. The results also indicate a high degree of isomer-specificity in the cytostatic activity of MDiCQAs. Unfortunately, all compounds (**4**, **9**, and **3**) that exhibited cytostatic activity against tumor cells also affected nontumorous Vero E6 cells. In particular, the inhibition of Vero E6 cells by the MDiCQAs (**4** and **9**) can be considered as a strong effect, and that of the FMGl (**3**) as a weaker one, with IC_50_ values below 10 μM and around 40 μM, respectively. Previously, 1,3-dicaffeoyl-5-malonyl-δ-quinide, a compound closely related to the MDiCQAs analyzed in our study, demonstrated nonselective in vitro cytotoxicity against both human leukemia cells (MOLM-13) and noncancerous rat kidney epithelial cells (NRK).^40^

**Table 3:** Cytostatic Effect of the Isolated Compounds^a^ **3**, **4**, and **9** on Vero E6 Cells and Human Colorectal Carcinoma (HT-29), Glioma (U87), Hepatoblastoma (HepG2), Leukemia (HL-60), and Melanoma (A2058)

	IC_50_ (μM)^b^			
cell line	3	4	9	Daunomycin
Vero E6	39.8 ± 2.0	9.9 ± 1.4	9.5 ± 3.1	1.7 ± 0.5
A2058	21.0 ± 3.0	13.5 ± 1.5	11.9 ± 2.2	0.2 ± 0.01
U87	86.3 ± 9.4	>100	48.5 ± 4.6	0.6 ± 0.05
HL-60	51.4 ± 6.7	29.7 ± 4.6	>100	0.16 ± 0.03
HepG2	27.5 ± 3.4	25.6 ± 2.3	>100	0.54 ± 0.15
HT-29	>100	>100	47.9 ± 2.3	0.9 ± 0.1

a The IC_50_ values of compounds **1, 2,** and **5–8** exceeded 100 μM for all tested cell lines; therefore, they are not included in Table 3. Note: These compounds were not tested on HL-60 cells.

b Results are presented as means ± SD, calculated from four parallel tests performed two times independently.

In summary, the phytochemical study of *A. cerefolium*, *A. sylvestris*, and *C. bulbosum*, closely related edible plants with some significance in natural medicine, led to the identification of five new MDiCQAs and one new FMGl. Three FMGls, previously identified in other plants, were also detected. The HPLC-MS/MS spectra of the four isomeric MDiCQAs revealed diagnostic differences in key fragment ion intensities, enabling their unambiguous identification directly in plant tissues without the use of authentic standards, which represents a significant analytical advantage. Considering the possibility of in-source oxidation of MDiCQAs and luteolin-7-*O*-(2″,6″-di-*O*-malonyl)-β-D-glucoside, their MS spectra displaying ions with masses 2 Da and 4 Da lower than expected will not hinder their future identification. The organ- and vegetation-phase-specific accumulation of MDiCQAs and FMGls was confirmed, allowing the identification of optimal tissues with relatively high levels of the selected compounds. This knowledge facilitated their efficient isolation through a one-step preparative HPLC method, thus streamlining access to these metabolites for further studies. The isolated compounds exhibited significant DPPH radical scavenging activity, except for kaempferol-3-*O*-(6″-*O*-malonyl)-β-D-glucoside. This flavonoid (**3**), along with two MDiCQAs bearing caffeoyl moieties at the C-1 and C-5 positions and a malonyl group at C-3 of the central quinic acid core (**4** and **9**), demonstrated non-cancer cell-specific *in vitro* cytostatic activity. However, since MDiCQAs **5**, **6**, and **7**, as well as FMGls **1**, **2**, and **8** demonstrate significant radical scavenging activity without *in vitro* toxicity to healthy cells, further testing for their medicinal potential is warranted.

## Supplementary Material



## Data Availability

The raw NMR spectra for compounds **4–9** are freely available on Zenodo with DOI: 10.5281/zenodo.14937953. Note: right now, we have uploaded the spectra on Zenodo and will publish the spectra upon acceptance of our manuscript.

## Notes

Additional details regarding materials and methods, HPLC-UV calibration curves, HR-MS analysis data, HR-MS, and HR-MS/MS spectra of compounds, composition of plant samples, 2D NMR correlations of compounds, 1D and 2D NMR spectra of compounds (PDF)

## Abbreviations Used

CQAs: caffeoylquinic acids
HPLC: high-performance liquid chromatography
HR-MS/MS: high-resolution tandem mass spectrometry
MDiCQAs: malonyl-dicaffeoylquinic acids
ESI: electrospray ionization
BVC: beginning of the vegetation cycle
FS: flowering stage
DiMDiCQA: dimalonyl-dicaffeoylquinic acid
CID: collision-induced dissociation
FMGls: flavonoid-malonyl-glucosides
