# Supplementary material for: Malonyl-Caffeoylquinic Acids and Malonyl-Flavonoid-Glucosides from Three Edible Apiaceae Plants Anthriscus Cerefolium, Anthriscus Sylvestris, and Chaerophyllum Bulbosum
Source: ACS Omega. 2025 Sep 22;10(38):43656–68. doi: 10.1021/acsomega.5c03770 (PMC12489727; doi:10.1021/acsomega.5c03770)
Supplement: Supplementary file 1 [file ao5c03770_si_001.pdf]

SUPPORTING INFORMATION

Malonyl-Caffeoylquinic Acids and Malonyl-Flavonoid-Glucosides from Three Edible Apiaceae Plants *Anthriscus cerefolium*, *Anthriscus sylvestris*, and *Chaerophyllum bulbosum*

Adila Nazli[1], Mária Gáborová[2], Tim Ausbüttel[1, 3], Bence Stipsicz[4, 5], Gergő Tóth[6, 7], Szilvia Bősze[5, 8], Szabolcs Béni[9, *], Imre Boldizsár[1, 3, 7, *]

[1] Department of Pharmacognosy, Semmelweis University, 1085 Budapest, Hungary.

[2] Department of Natural Drugs, Faculty of Pharmacy, Masaryk University, 61200 Brno, Czechia.

[3] Department of Plant Anatomy, Institute of Biology, Eötvös Loránd University, Pázmány Péter sétány 1/C, 1117 Budapest, Hungary.

[4] Institute of Biology, Doctoral School of Biology, ELTE Eötvös Loránd University, Pázmány Péter sétány 1/C, 1117 Budapest, Hungary.

[5] HUN-REN-ELTE Research Group of Peptide Chemistry, Hungarian Research Network, ELTE Eötvös Loránd University, Pázmány Péter sétány 1/A, 1117 Budapest, Hungary.

[6] Department of Pharmaceutical Chemistry, Semmelweis University, Hőgyes Endre u. 9, 1092 Budapest, Hungary.

[7] Center for Pharmacology and Drug Research & Development, Semmelweis University, 1085 Budapest, Hungary.

[8] Department of Genetics, Cell- and Immunobiology, Semmelweis University, Nagyvárad tér 4, 1089 Budapest, Hungary.

[9] Integrative Health and Environmental Analysis Research Laboratory, Department of Analytical Chemistry, Institute of Chemistry, Eötvös Loránd University, 1117 Budapest, Hungary.

[*] Corresponding authors. E-mail addresses: boldizsar.imre@pharma.semmelweis-univ.hu (I. B)

szabolcs.beni@ttk.elte.hu (S. B.)

MATERIALS AND METHODS

General Experimental Procedures.

Analytical HPLC with UV and High-Resolution Orbitrap Mass Spectrometry. A Dionex Ultimate 3000 HPLC system (3000RS diode array detector (DAD), TCC-3000RS column thermostat, HPG-3400RS pump, SRD-3400 solvent rack degasser, WPS-3000TRS autosampler) connected to an Orbitrap Q Exactive Focus Mass Spectrometer equipped with electrospray ionization (ESI) (Thermo Fisher Scientific, Waltham, MA, USA) was used. Column: Gemini NX-C18 column (150 × 3 mm; 3 µm) (Phenomenex, Torrance, CA, USA). Eluents 1: eluent A, 0.1% v/v formic acid, eluent B, acetonitrile:0.1% v/v formic acid (80:20, v/v); 2: eluent A, H_2O (without formic acid), eluent B, acetonitrile:0.1% v/v formic acid (80:20, v/v); 3: eluent A, 0.3% v/v formic acid, eluent B, acetonitrile:0.1% v/v formic acid (80:20, v/v). Gradient program 1: 0.0 min, 20% B; 15.0 min, 90% B (linear gradient); 18.0 min, 90% B (isocratic). Gradient program 2: 0.0 min, 15% B; 15.0 min, 50% B (linear gradient); 16.0 min, 90% B (linear gradient); 19.0 min, 90% B (isocratic). Flow rate: 0.4 mL/min; column temperature: 25 °C; injected volume: 1.0–5.0 µL. Ionization: ESI in positive and negative ionization mode (switching mode). Fragmentations: data-independent acquisition (DIA) with isolation widths of 100–300 *m/z*, 295–500 *m/z*, 495–800 *m/z*, and 795–1100 *m/z*, and collision energies of 15, 30, and 45 eV; parallel reaction monitoring PRM method with isolation width of 0.4 *m/z*, and collision energies ranging from 10 eV to 45 eV. Resolution: full MS, 70,000; MS/MS, 35,000. Full MS scans range: 100–1500 *m/z*. UV scan range: 230–600 nm. Each HPLC-UV chromatogram represents the sum of signal intensities detected within this range (other operation parameters were consistent with those described in our recent article (Tóth et al., 2023).

Quantification of compounds: An external standard method was employed using an HPLC-DAD technique. The compounds used as calibration standards were isolated in this study. Isolated

compounds **1**–**4**, **8**, and **9**, each with a purity exceeding 95% as determined by HPLC-UV, were used as calibration standards (Supporting Information Figure S1a). Linear regression analyses were performed for the isolated compounds (**1**–**4**, **8**, **9**) in the following concentration ranges: 0.2670–100.0 μg/mL (**1**), 0.4120–100.0 μg/mL (**2**, **3**, **8**), and 0.240–300.0 μg/mL (**4**, **9**). All compounds exhibited r^2 values greater than 0.999 (Supporting Information Figure S1b). The quantities of compounds **5**, **6**, and **7**, with purities of 93.7%, 90.5%, and 83.0%, respectively, were calculated using the calibration curve of compound **4** (Supporting Information Figure S1a).

Isolation of Compounds by Preparative HPLC. A Dionex UltiMat 3000 HPLC System (VWD-3400RS photometer, HPG-3200BX pump, WPS-3000TSL autosampler, VF-F11-A-01 Fraction Collector F) (Thermo Fisher Scientific, Waltham, MA, USA) was used. Detection: 348 and 330 nm. Column: Gemini NX-C18 column (250 × 10 mm; 5 μm) (Phenomenex, Torrance, CA, USA). Eluents: eluent A, 0.1% v/v formic acid, eluent B, acetonitrile. Gradient program: 0.0 min, 15% B; 4.0 min, 15.0% B (isocratic); 32 min, 25% B (linear gradient); 42.0 min, 90% B (linear gradient).

NMR Analysis. NMR spectra of isolated compounds were recorded with a Bruker Avance III HD 500 (500/150 MHz) spectrometer fitted with Prodigy cryo-probe head at 295 K while the band selective-HMBC spectrum of compound **6** was recorded at 328 K. Compounds were dissolved in DMSO-d_6 and D_2O. Spectra acquisition was accomplished through Bruker TopSpin 3.5 software utilizing standard pulse sequences provided in their software library. Spectra were processed and analyzed by the MestreNova NMR processor. ^{1}H and ^{13}C chemical shifts (δ) are provided in ppm relative to TMS (tetramethylsilane), while coupling constants (*J*) are calculated in Hz. Resonance assignments were inferred by 1D ^{1}H NMR, ^{13}C NMR and 2D ^{1}H–^{1}H COSY, ^{1}H–^{1}H ROESY, ^{1}H–^{13}C HSQC, and ^{1}H–^{13}C HMBC experiments.

Materials and Reagents. The materials and reagents applied in analyzing and isolating plant metabolites were all of the analytical reagent grades of the highest purity available. Acetonitrile,

distilled water, formic acid, and methanol were obtained from Reanal (Hungary), while D_2O and DMSO-d_6 were sourced from VWR Chemicals (Belgium). DPPH (2,2-diphenyl-1-picrylhydrazyl), chlorogenic acid, quercetin, rutin, kaempferol, and Trolox were purchased from Sigma-Aldrich (Steinheim, Germany). DMEM (Dulbecco's Modified Eagle's Medium), PBS (phosphate-buffered saline), L-glutamine, and trypan blue were supplied by Lonza (Basel, Switzerland). Trypsin, nonessential amino acids, and penicillin-streptomycin were obtained from Gibco (Thermo Fisher Scientific, Waltham, MA), while FBS (fetal bovine serum) was sourced from EuroClone (Pero, Italy).

Plant Material. Plant samples were collected from different Hungarian locations in the Buda Hills at the beginning of the vegetation cycle (January and February) and in the flowering stage (April and May) between 2022 and 2024. At each sampling time, at least ten plants were collected. Organs were manually separated and pooled to prepare the root, leaf, and inflorescence samples. Samples were lyophilized on the day of collection. The voucher specimens of the dried samples are deposited in the Department of Plant Anatomy, Eötvös Loránd University, Budapest, Hungary.

Preparation of Plant Extracts for Analysis and Isolation. Lyophilized and pulverized plant tissues (10.0 mg) were extracted with 3.0 mL of methanol at 60 °C for 30 minutes in 5 mL screw-capped vials. The extracts were centrifuged at 5,000 rpm using a standard laboratory centrifuge before HPLC-UV-HR-MS analysis. For compound isolation, pulverized plant tissues (2 g) were extracted thrice with 15 mL of methanol at 60 °C in screw-capped vials for 30 minutes each. The centrifuged supernatants were combined and dried using a standard laboratory rotary vacuum evaporator. The dried extracts were dissolved in 5 mL of methanol and centrifuged at 5,000 rpm. After centrifugation, the supernatant was filtered using Minisart RC 15 syringe filters (0.2 µm pore diameter, Sartorius AG, Goettingen, Germany) before preparative HPLC separation.

Antioxidant Activity Tests. A DPPH radical scavenging assay was used according to a previously published protocol.[13] Serial dilutions of compounds (prepared in methanol) were incubated with DPPH solution for 6 min at room temperature. The reduction in absorbance was measured with a HITACHI U-2000 spectrophotometer (Hitachi Ltd., Tokyo, Japan) at 515 nm. Half maximal inhibitory concentration values (IC_{50}, μM) were calculated, and analysis was performed in triplicate.

Determination of the *In Vitro* Cytostatic Effects of Compounds. *In vitro,* the cytostatic effect of the compounds was determined on various human tumor- and non-human primate Vero E6 cells using Alamar Blue end-point viability assay. Cells were cultured in DMEM medium supplemented with 10% FBS, 2 mM L-glutamine, 50 IU/mL penicillin/50 μg/mL streptomycin antibiotic cocktail, 1 mM sodium pyruvate and 1% non-essential amino acid mixture at 37 °C in a humidified atmosphere with 5% CO_2. At the confluent state, cells were plated into 96-well tissue culture plates with the initial cell number of 5.0×10^3 cells/well. Cells were treated with the compounds after 24 hours in a serum-free medium containing 1.0% (v/v) DMSO at 0.08-100 μM concentration. Control cells were treated with serum-free medium or DMSO (c=1.0%, v/v) containing serum-free medium. After overnight incubation, cells were washed twice with a serum-free medium; then, they were cultured for another 72 hours in the complete culturing medium at 37 °C. Following that, 22.5 μL Alamar Blue (resazurin sodium salt, Merck) solution (0.15 mg/mL in PBS) was added to each well and after 4 hrs of incubation, the fluorescence was measured at λEx = 530/30 and λEM = 610/10 nm using a Synergy H4 multi-mode microplate reader (BioTek). All measurements were performed in quadruplets, and the mean IC_{50} values and ± SEM were represented.

(**a**)

(**b**)

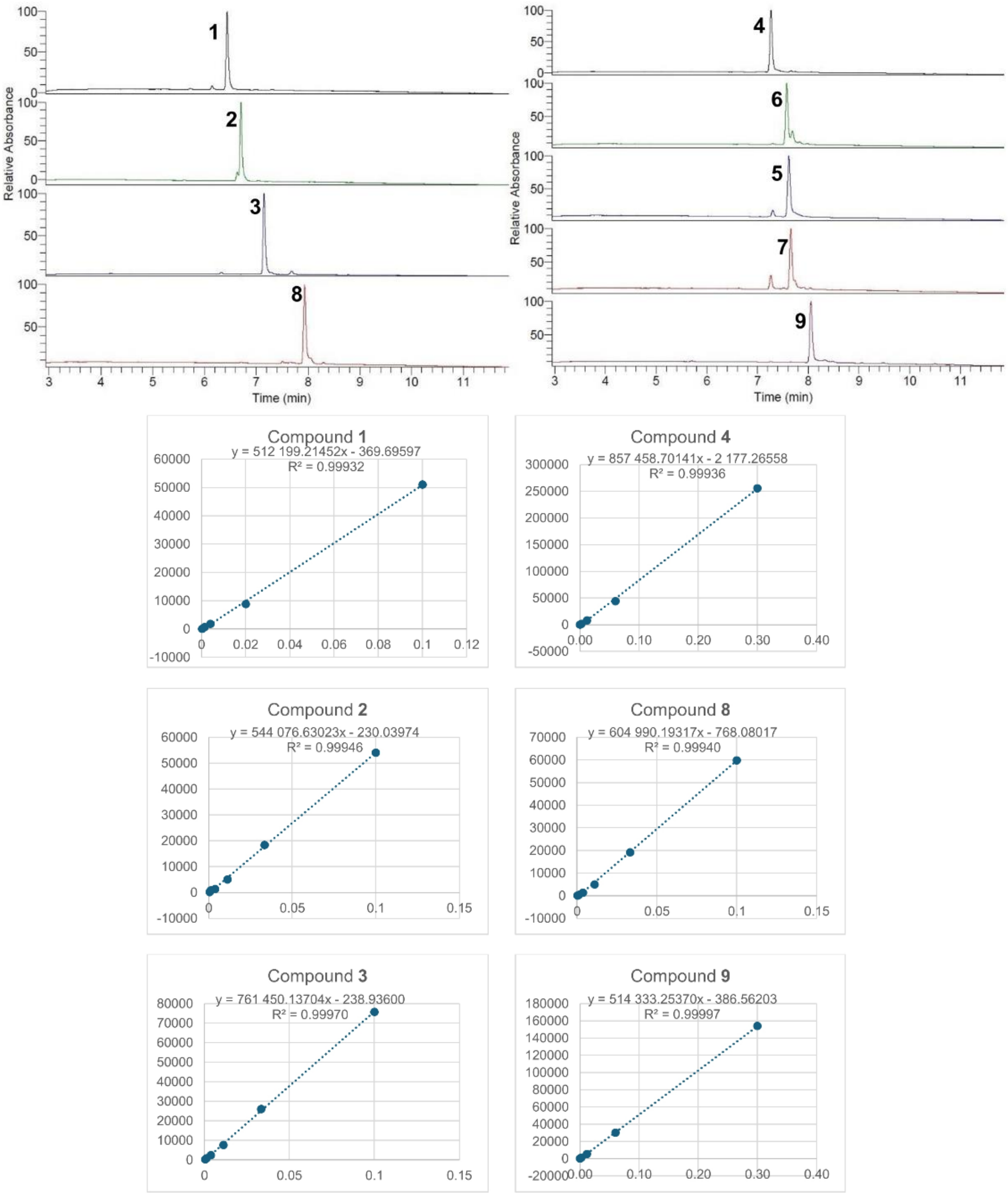

Figure S1. (**a**) HPLC-UV (λ = 230–600 nm, total scan) analysis of the isolated flavonoid-malonyl-glucosides (**1**, **2**, **3**, **8**) and malonyl-dicaffeoylquinic acids (**4**–**7**, **9**) using gradient program 1. Peak numbers (in bold) correspond to the numbering of the identified compounds: quercetin-3-*O*-(6"-*O*-malonyl)-β-D-glucoside (**1**), luteolin-7-*O*-(6"-*O*-malonyl)-β-D-glucoside (**2**), kaempferol-3-*O*-(6"-*O*-malonyl)-β-D-glucoside (**3**), 1,5-dicaffeoyl-3-malonylquinic acid (**4**), 3,5-dicaffeoyl-1-malonylquinic acid (**5**), 3,5-dicaffeoyl-4-malonyl-*epi*-quinic acid (**6**), 1,5-dicaffeoyl-4-malonylquinic acid (**7**), luteolin-7-*O*-(2",6"-di-*O*-malonyl)-β-D-glucoside (**8**), and 1,5-dicaffeoyl-3,4-dimalonylquinic acid (**9**). (**b**) HPLC-UV calibration curves of quercetin-3-*O*-(6"-*O*-malonyl)-β-D-glucoside (**1**), luteolin-7-*O*-(6"-*O*-malonyl)-β-D-glucoside (**2**), kaempferol-3-*O*-(6"-*O*-malonyl)-β-D-glucoside (**3**), 1,5-dicaffeoyl-3-malonylquinic acid (**4**), luteolin-7-*O*-(2",6"-di-*O*-malonyl)-β-D-glucoside (**8**), and 1,5-dicaffeoyl-3,4-dimalonylquinic acid (**9**); x: concentration, mg/mL; y: peak area. The HPLC-UV chromatograms were recorded at a wavelength range of 230–600 nm, detecting the total signal intensity within this range.

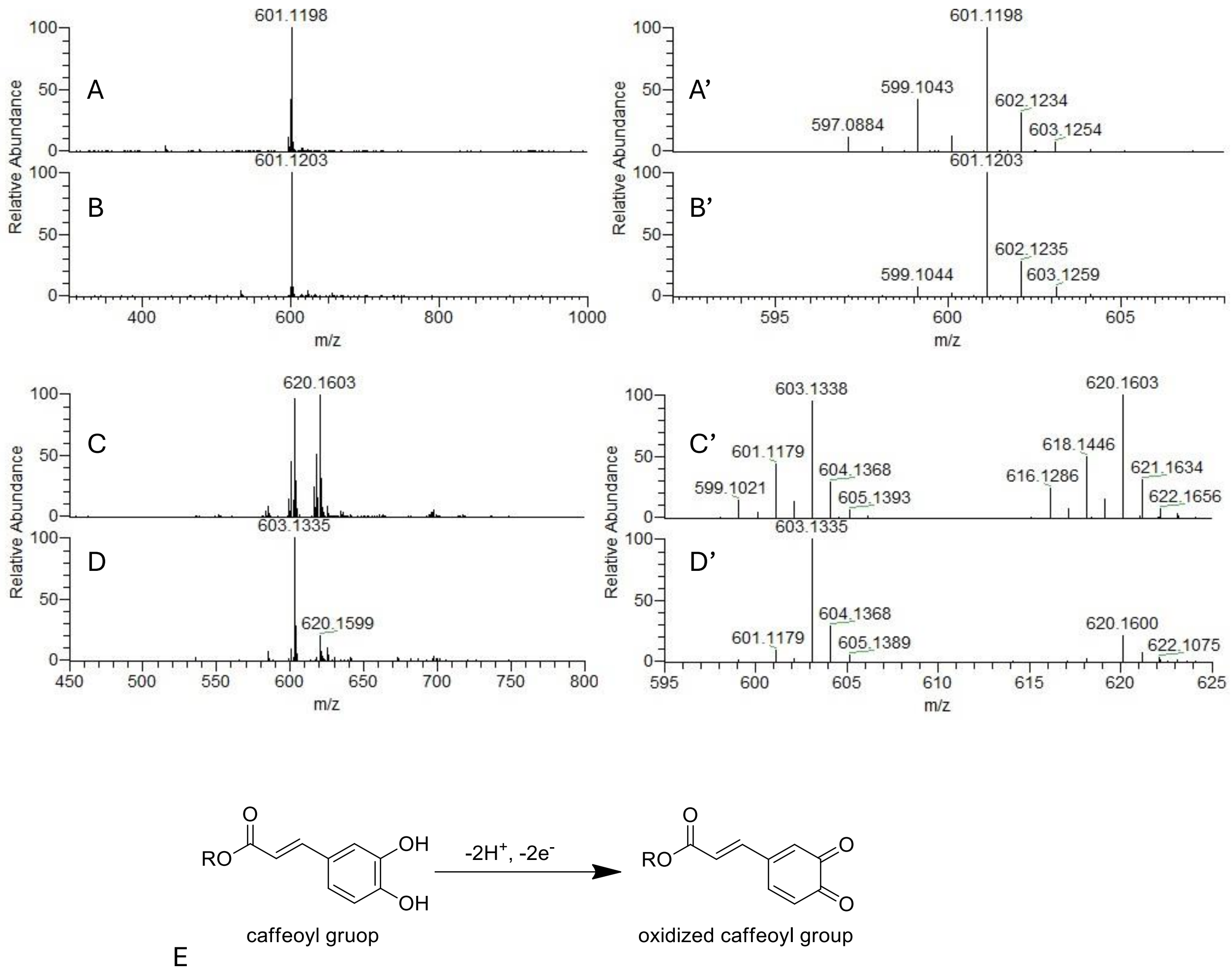

Figure S2. The mass spectra of compound **4**, acquired in negative (A, B) and positive (C, D) ionization modes, obtained from the HPLC separations using eluents with 0.1% formic acid (A, C) and 0.3% formic acid (B, D), along with the proposed oxidation pathway of the caffeoyl group (E). The corresponding spectra are also presented in a narrower mass range (A’–D’).

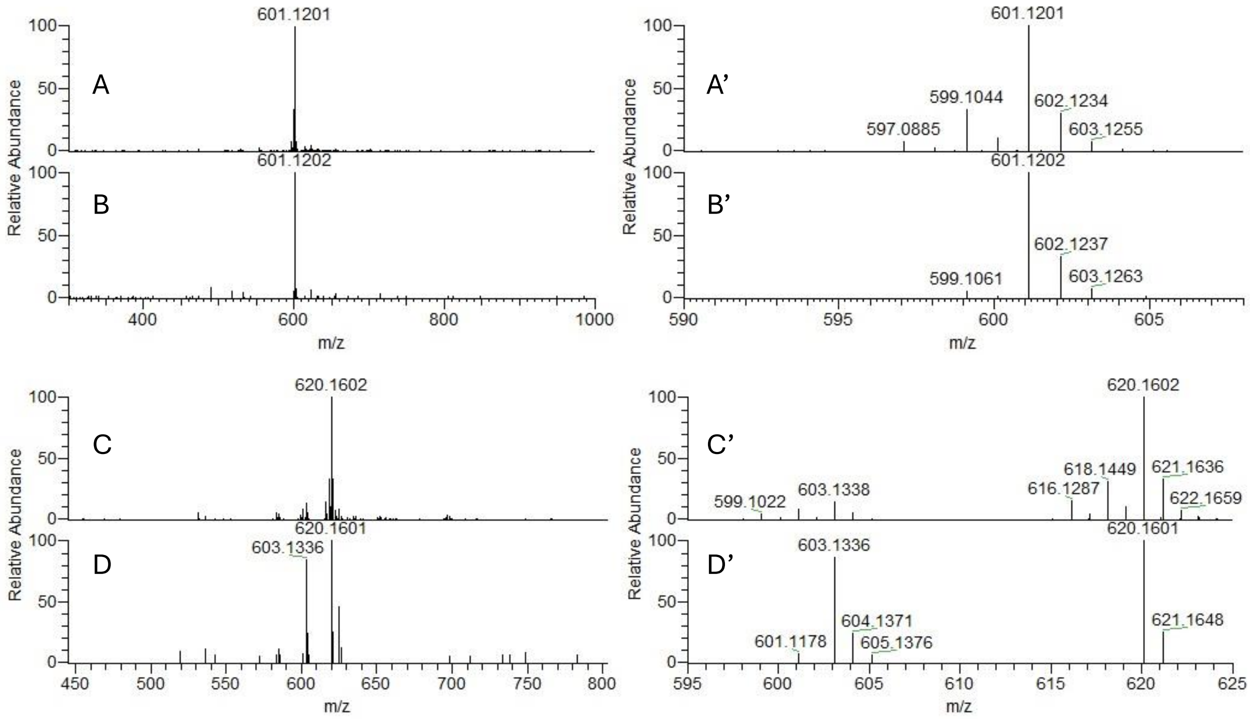

Figure S3. The mass spectra of compound **7**, acquired in negative (A, B) and positive (C, D) ionization modes, obtained from the HPLC separations using eluents with 0.1% formic acid (A, C) and 0.3% formic acid (B, D). The corresponding spectra are also presented in a narrower mass range (A'–D').

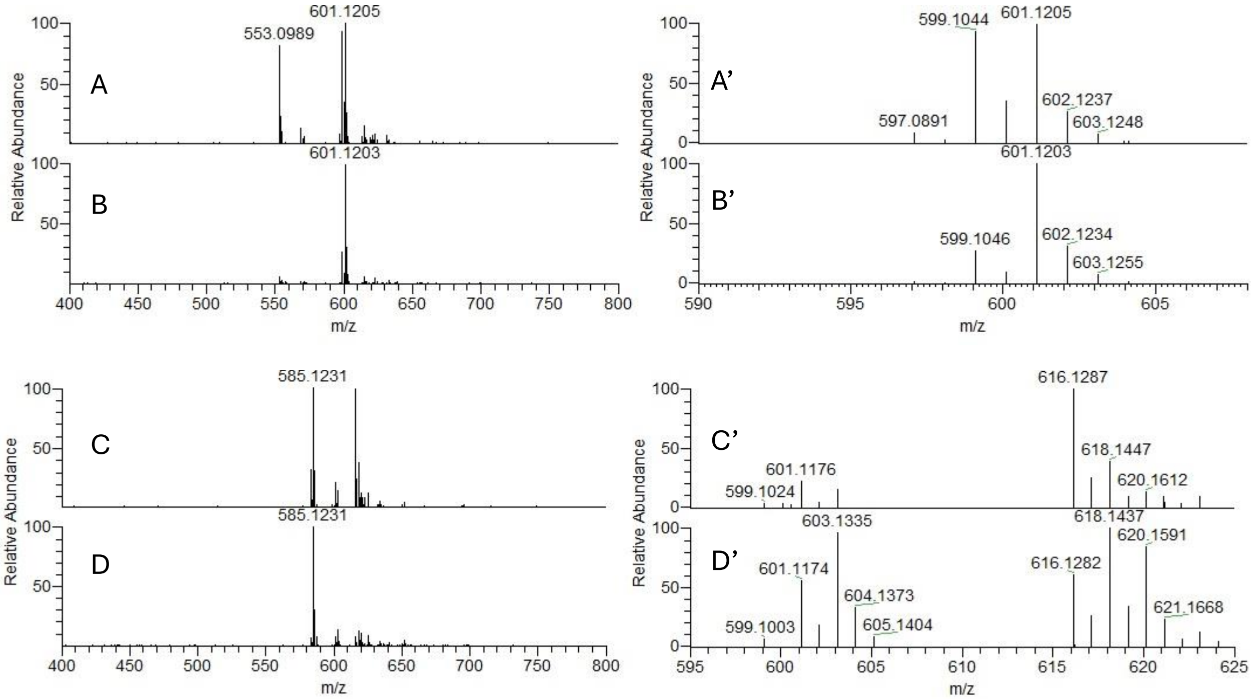

Figure S4. The mass spectra of compound **5**, acquired in negative (A, B) and positive (C, D) ionization modes, obtained from the HPLC separations using eluents with 0.1% formic acid (A, C) and 0.3% formic acid (B, D). The corresponding spectra are also presented in a narrower mass range (A’–D’).

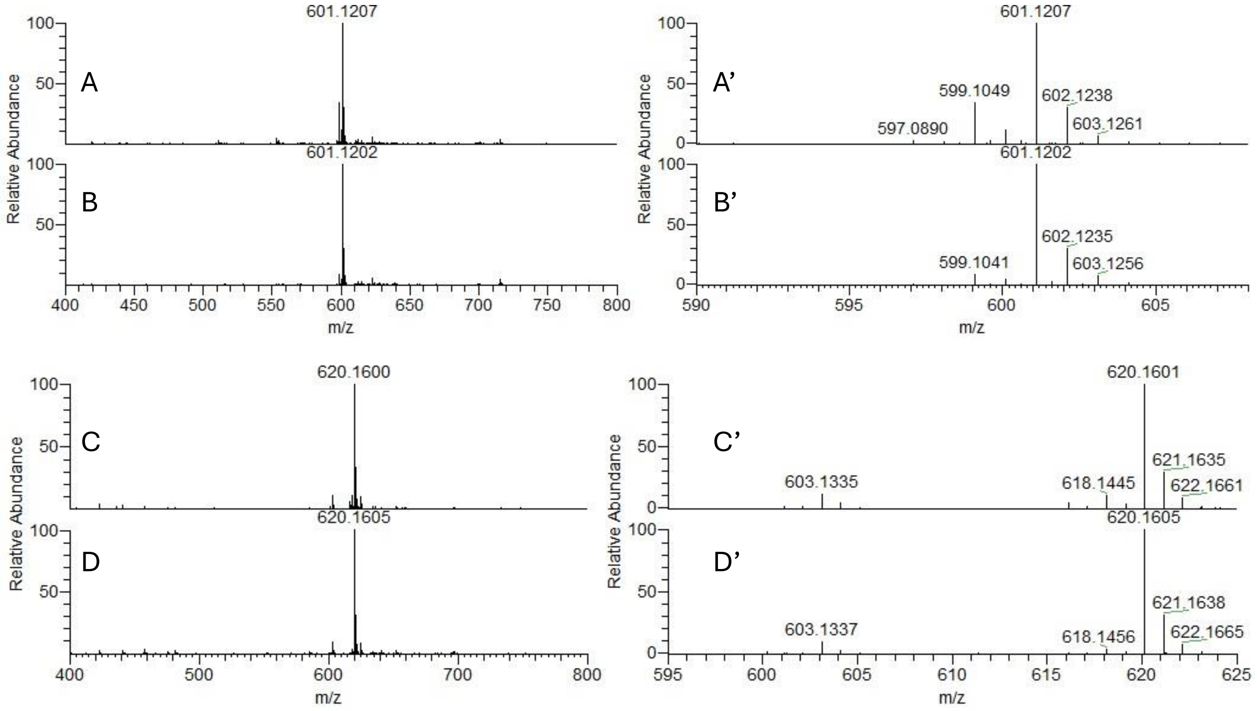

Figure S5. The mass spectra of compound **6**, acquired in negative (A, B) and positive (C, D) ionization modes, obtained from the HPLC separations using eluents with 0.1% formic acid (A, C) and 0.3% formic acid (B, D). The corresponding spectra are also presented in a narrower mass range (A’–D’).

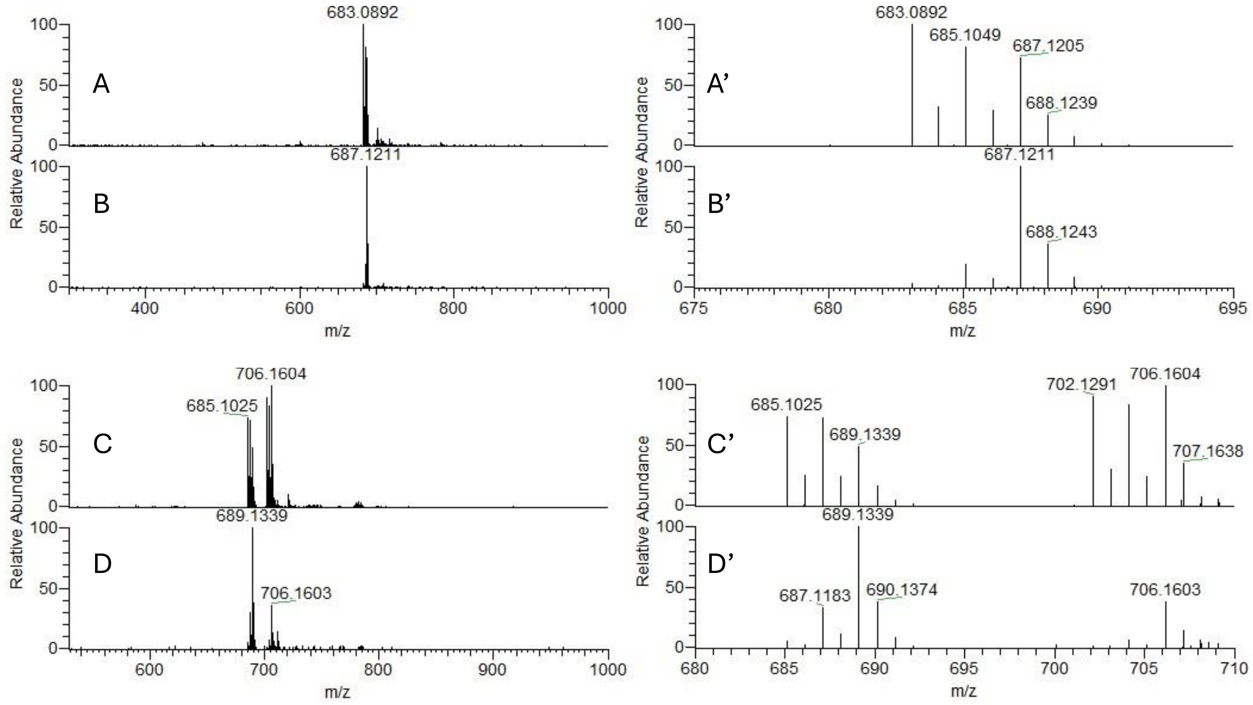

Figure S6. The mass spectra of compound **9**, acquired in negative (A, B) and positive (C, D) ionization modes, obtained from the HPLC separations using eluents with 0.1% formic acid (A, C) and 0.3% formic acid (B, D). The corresponding spectra are also presented in a narrower mass range (A’–D’).

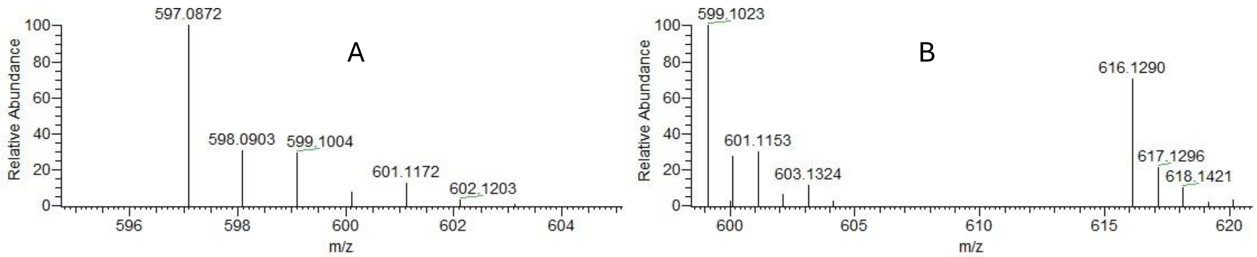

Figure S7. The mass spectra of compound **4**, acquired in negative (A) and positive (B) ionization modes, obtained from the HPLC separation using eluent without formic acid.

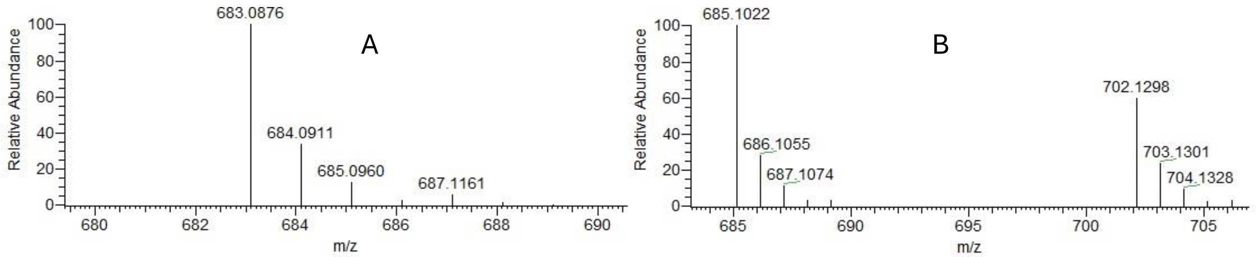

Figure S8. The mass spectra of compound **9**, acquired in negative (A) and positive (B) ionization modes, obtained from the HPLC separation using eluent without formic acid.

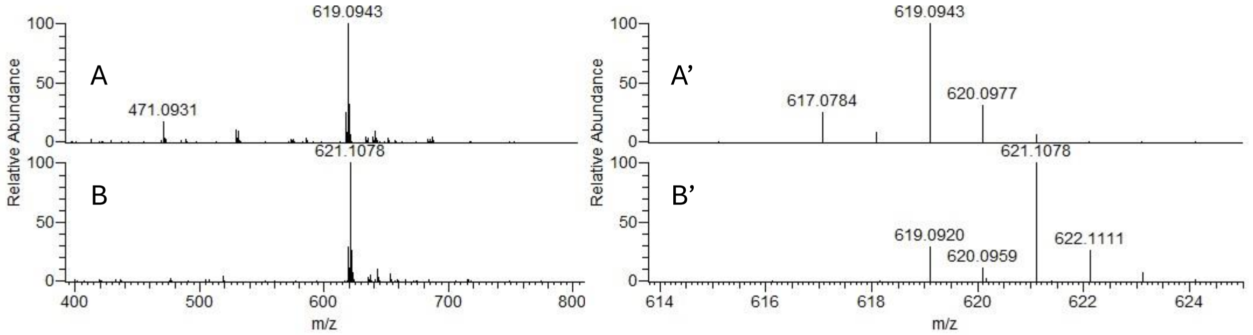

Figure S9. The mass spectra of compound **8**, acquired in negative (A) and positive (B) ionization modes, obtained from the HPLC separations using eluents with 0.1% formic acid. The corresponding spectra are also presented in a narrower mass range (A’, B’).

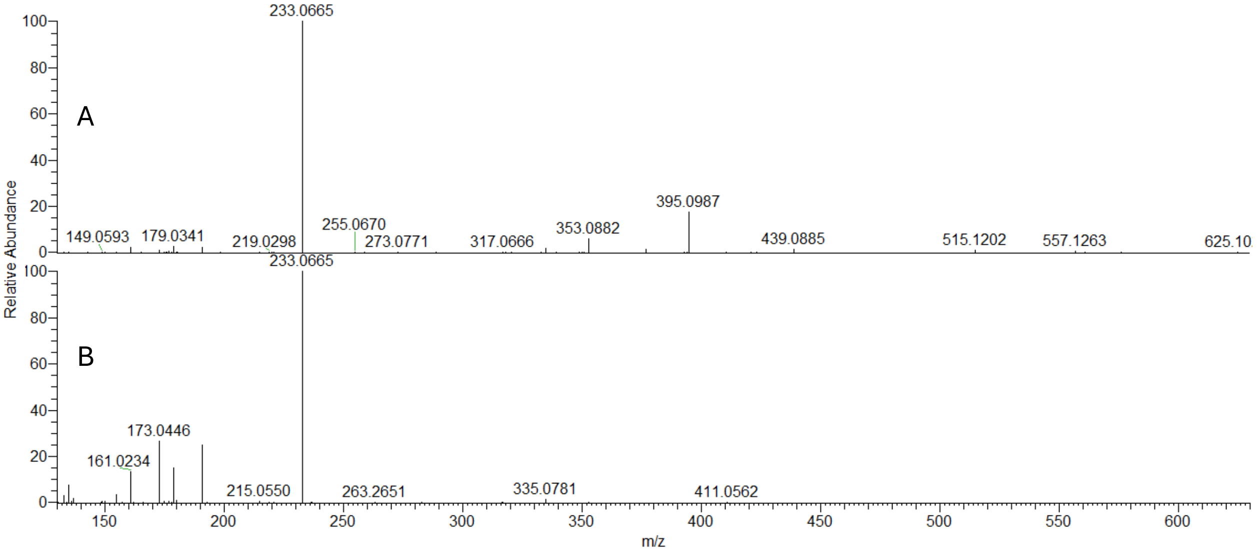

(**a**)

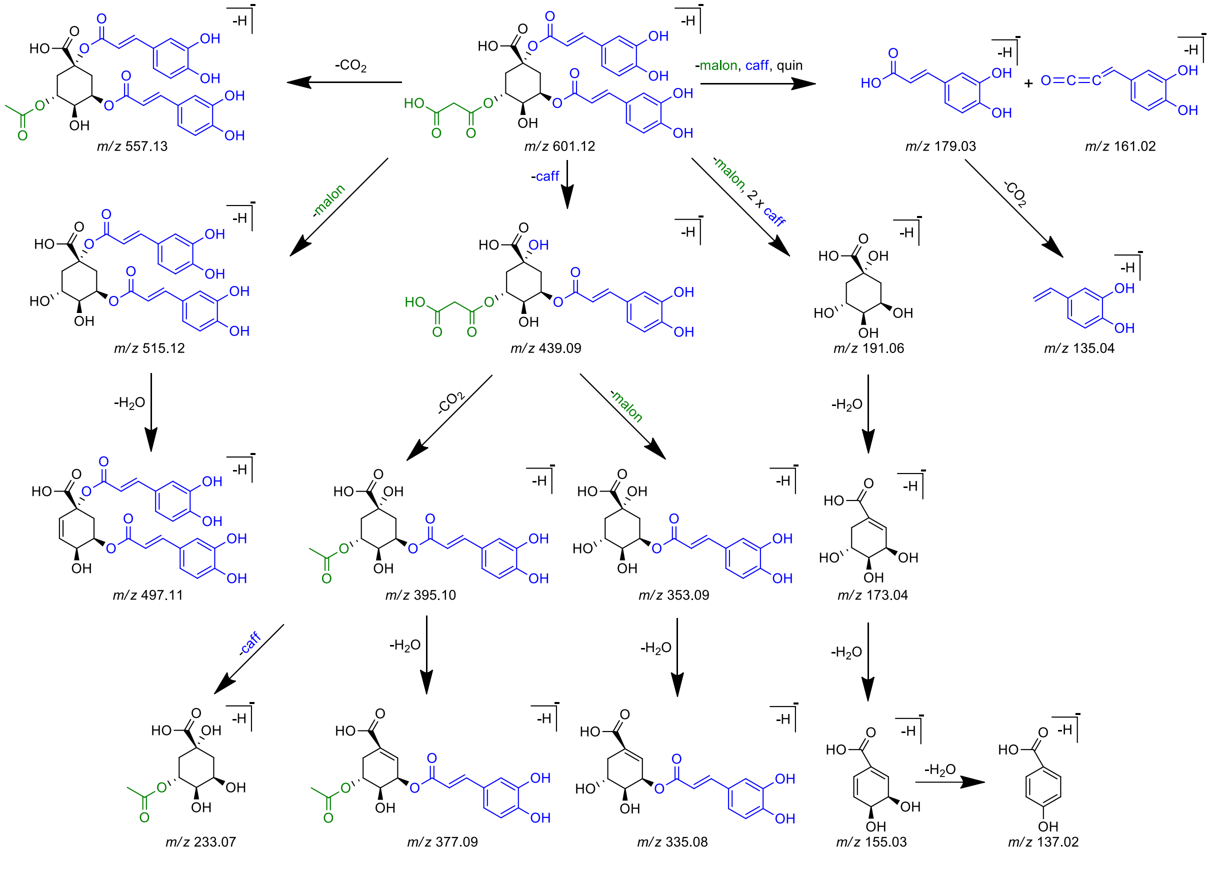

(**b**)

Figure S10. (**a**) The tandem mass spectra of the deprotonated compound **4**, obtained at collision induced dissociation (CID) energies of 25 eV (A) and 45 eV (B). (**b**) Proposed structures of fragment ions observed in the tandem mass spectra of deprotonated compound **4**.

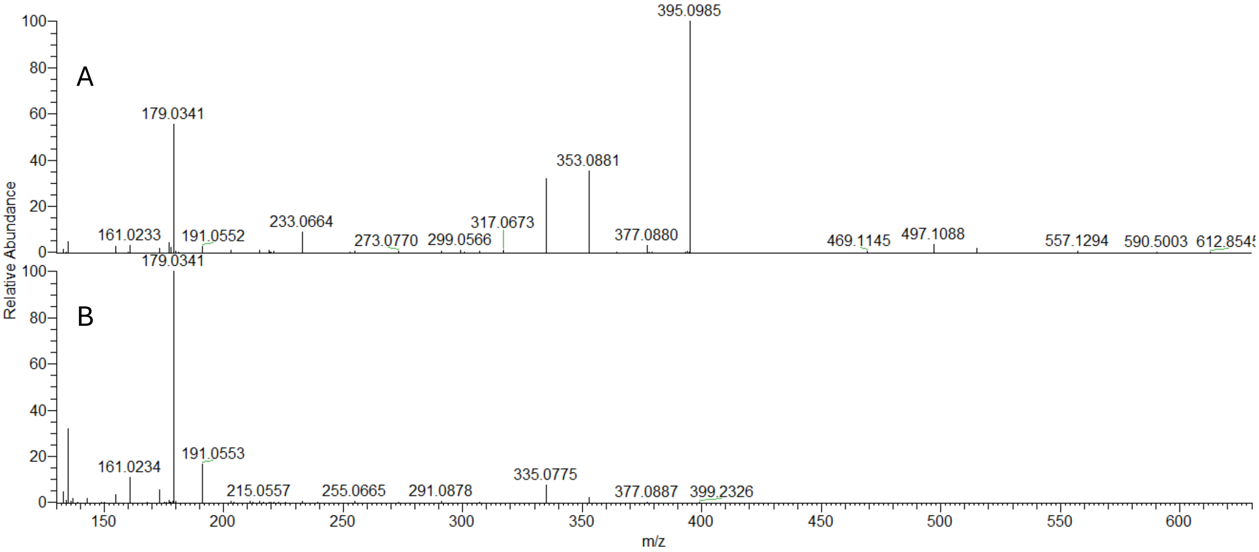

Figure S11. The tandem mass spectra of the deprotonated compound **5**, obtained at collision induced dissociation (CID) energies of 25 eV (A) and 45 eV (B).

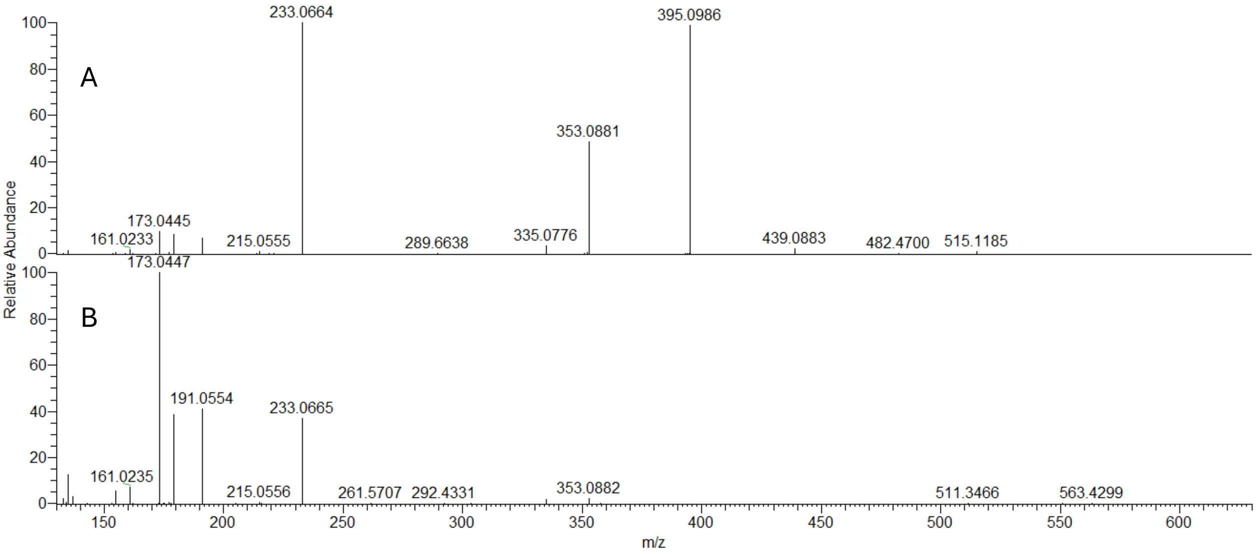

Figure S12. The tandem mass spectra of the deprotonated compound **6**, obtained at collision induced dissociation (CID) energies of 25 eV (A) and 45 eV (B).

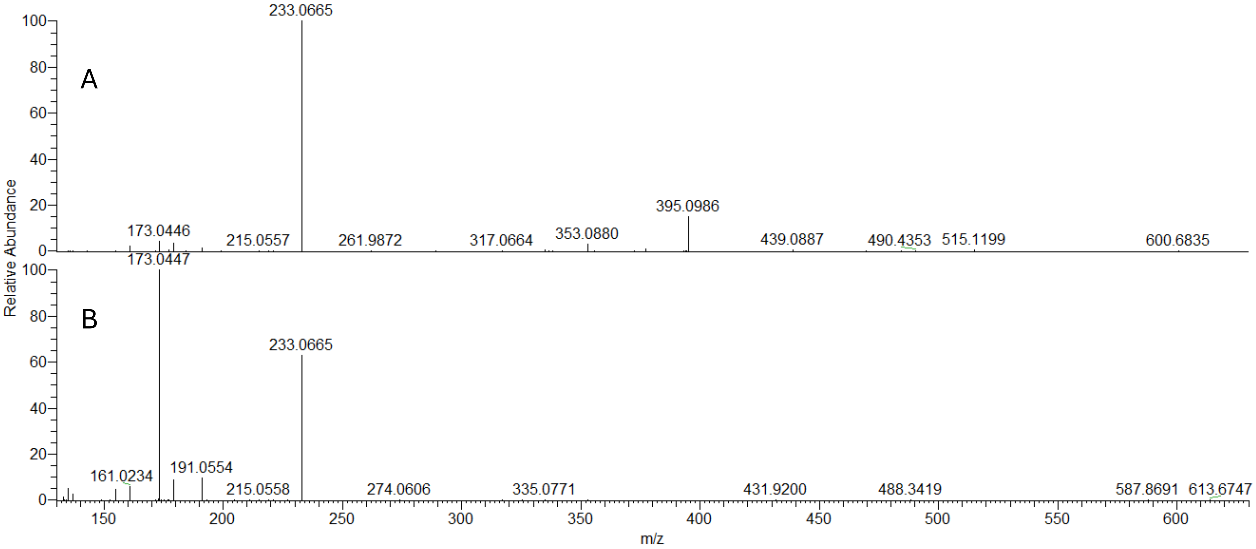

Figure S13. The tandem mass spectra of the deprotonated compound **7**, obtained at collision induced dissociation (CID) energies of 25 eV (A) and 45 eV (B).

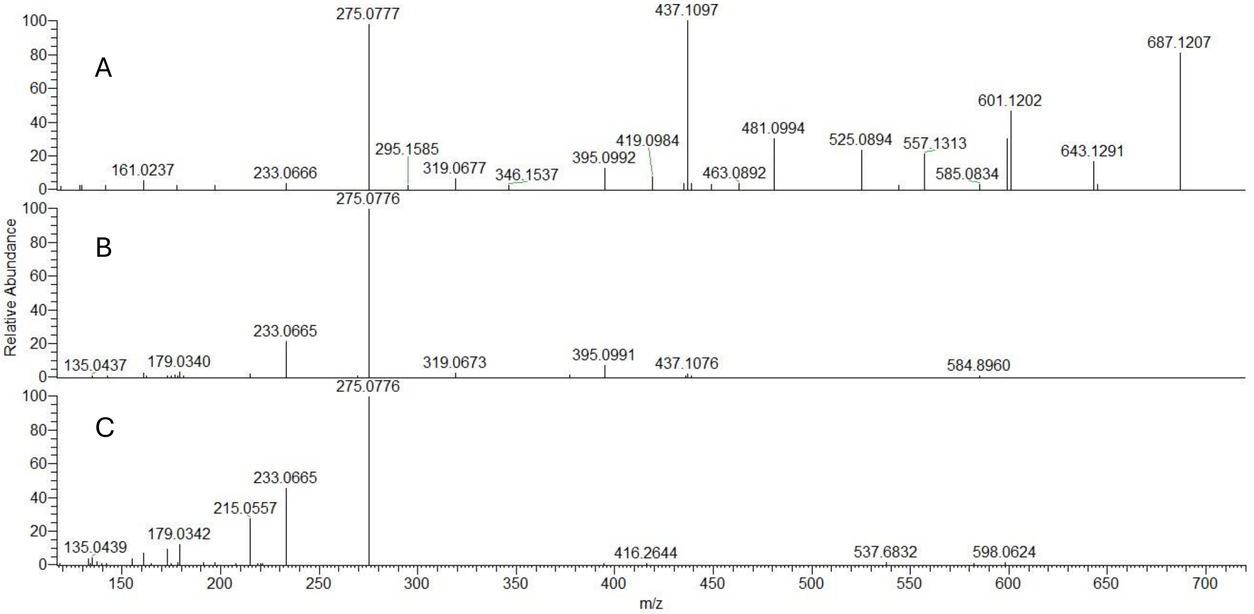

Figure S14. The tandem mass spectra of the deprotonated compound **9**, obtained at collision induced dissociation (CID) energies of 15 eV (A), 30 eV (B), and 45 eV (C).

Table S1.

Mass Spectrometry (MS) Data of Malonyl-Dicaffeoylquinic Acids (MDiCQAs) **4**–**7** and **9**, Obtained from the Mass Spectra of These Compounds, Determined by HPLC High-resolution Orbitrap-MS Using Negative and Positive Electrospray Ionization (ESI) Modes.

No.[a]	formula	measured and calculated masses (*m/z*) of ions and the difference (ppm)[b] between them						
		ionization mode: positive						
		detected ions[c]	$[M+H]^+$	$[M+H\text{-}2H]^+$	$[M+H\text{-}4H]^+$	$[M+NH_4]^+$	$[M+NH_4\text{-}2H]^+$	$[M+NH_4\text{-}4H]^+$
	$C_{28}H_{26}O_{15}$	calculated mass	603.1344	601.1188	599.1031	620.1610	618.1453	616.1297
**4**		measured mas	603.1338	601.1179	599.1021	620.1603	618.1445	616.1286
		mass error	-1.088	-1.458	-1.730	-1.057	-1.416	-1.778
**5**		measured mass	603.1342	601.1176	599.1024	620.1612	618.1447	616.1287
		mass error	-0.475	-1.974	-1.312	0.314	-1.028	-1.583
**6**		measured mass	603.1335	601.1181	-	620.1601	618.1445	616.1285
		mass error	-1.602	-1.158	-	-1.444	-1.319	-1.973
**7**		measured mass	603.1338	601.1182	599.1022	620.1602	618.1445	616.1287
		mass error	-0.989	-0.942	-1.529	-1.250	-1.416	-1.583
	$C_{31}H_{28}O_{18}$	calculated mass	689.1348	687.1192	685.1035	706.1614	704.1457	702.1301
**9**		measured mass	689.1339	687.1182	685.1025	706.1604	704.1447	702.1291
		mass error	-1.350	-1.397	-1.460	-1.401	-1.462	-1.423
		ionization mode: negative						
		detected ions[c]	$[M-H]^-$	$[M-H\text{-}2H]^-$	$[M-H\text{-}4H]^-$			
	$C_{28}H_{26}O_{15}$	calculated mass	601.1199	599.1042	597.0886			
**4**		measured mass	601.1198	599.1043	597.0884			
		mass error	-0.138	0.112	-0.256			
**5**		measured mass	601.1205	599.1044	597.0891			
		mass error	1.076	0.329	0.765			
**6**		measured mass	601.1207	599.1049	597.0890			
		mass error	1.292	1.030	0.665			
**7**		measured mass	601.1201	599.1044	597.0885			
		mass error	0.377	0.212	-0.156			
	$C_{31}H_{28}O_{18}$	calculated mass	687.1203	685.1046	683.0890			
**9**		measured mass	687.1205	685.1049	683.0893			
		mass error	0.368	0.413	0.444			

[a] The numbers correspond to 1,5-dicaffeoyl-3-malonylquinic acid (**4**), 3,5-dicaffeoyl-1-malonylquinic acid (**5**), 3,5-dicaffeoyl-4-malonyl-*epi*-quinic acid (**6**), 1,5-dicaffeoyl-4-malonylquinic acid (**7**), and 1,5-dicaffeoyl-3,4-dimalonylquinic acid (**9**).

[b] The differences between the measured and calculated masses (*m/z*) are expressed in ppm values.

[c] The MS spectra of the MDiCQAs, displaying detected ions, can be found in Supporting Information Figures S2–S6. Italicized hydrogens in the detected ion structures found within square brackets represent singly oxidized (-*2H*) and doubly oxidized (-*4H*) ions. These ions result from the loss of hydrogen atoms from the *ortho*-hydroxyl groups of one catechol unit or from the *ortho*-hydroxyl groups of two catechol units. This oxidation leads to the detection of ions with masses that are 2 Da or 4 Da lower than their non-oxidized counterparts.

Table S2.
Mass Spectrometry (MS) Data of Flavonoid-Malonyl-Glucosides (FMGls) **1**–**3** and **8**, Obtained from the Mass Spectra of These Compounds, Determined by HPLC High-resolution Orbitrap-MS Using Negative and Positive Electrospray Ionization (ESI) Modes.

No.[a]	formula	MS mode[b]	detected ion[c]	measured mass (m/z)	calculated mass (m/z)	mass error (ppm)
**1**	$C_{24}H_{22}O_{15}$	+	$[M+Na]^+$	573.0837	573.0851	-2.462
			$[M+H]^+$	551.1018	551.1031	-2.425
		-	$[M-H]^-$	549.0895	549.0886	1.615
**2**	$C_{24}H_{22}O_{14}$	+	$[M+Na]^+$	557.0886	557.0902	-2.794
			$[M+H]^+$	535.1074	535.1082	-1.629
		-	$[M-H]^-$	533.0945	533.0937	1.504
**3**	$C_{24}H_{22}O_{14}$	+	$[M+Na]^+$	557.0887	557.0902	-2.560
			$[M+H]^+$	535.1074	535.1082	-1.517
		-	$[M-H]^-$	533.0944	533.0937	1.391
**8**	$C_{27}H_{24}O_{17}$	+	$[M+H]^+$	621.1078	621.1086	-1.345
			$[M+H\text{-}2H]^+$	619.0920	619.0930	-1.608
		-	$[M-H]^-$	619.0943	619.0941	0.368
			$[M-H\text{-}2H]^-$	617.0784	617.0784	-0.085

[a] Compound numbers correspond to quercetin-3-*O*-(6"-*O*-malonyl)-β-D-glucoside (**1**), luteolin-7-*O*-(6"-*O*-malonyl)-β-D-glucoside (**2**), kaempferol-3-*O*-(6"-*O*-malonyl)-β-D-glucoside (**3**), luteolin-7-*O*-(2",6"-di-*O*-malonyl)-β-D-glucoside (**8**).
[b] High-resolution mass spectrometry (HR-MS) detection was operated in positive (+) and negative (-) ionization modes.
[c] The MS spectra of FMGl **8**, displaying detected ions, can be found in the Supporting Information Figure S9. Italicized hydrogens in the detected ion structures found within square brackets represent singly oxidized (-*2H*) ions. These ions result from the loss of hydrogen atoms from the *ortho*-hydroxyl groups of a catechol unit. This oxidation leads to the detection of ions with masses that are 2 Da lower than their non-oxidized counterparts.

Table S3.

Relative Abundance of Characteristic Ions in the Tandem Mass Spectra (MS2) of Malonyl-Dicaffeoylquinic Acids (MDiCQAs) **4**–**7**, Determined by HPLC High-Resolution Orbitrap Tandem Mass Spectrometry Using Negative Electrospray Ionization (ESI) Mode.

No.[a]	CID[b] (eV)	Relative abundances (%)[c] of characteristic ions (CIs)[e]																
	CI→	$[M-H]^-$	$[M-H-CO_2]^-$	$[M-H-MA]^-$	$[M-H-MA-H_2O]^-$	$[M-H-CA]^-$	$[M-H-CA-CO_2]^-$	$[M-H-CA-CO_2-H_2O]^-$	$[M-H-CA-MA]^-$	$[M-H-CA-MA-H_2O]^-$	$[M-H-2\times CA-CO_2]^-$	$[QA-H]^-$	$[CA-H]^-$	$[QA-H-H_2O]^-$	$[CA-H-H_2O]^-$	$[QA-H-2\times H_2O]^-$	$[QA-H-3\times H_2O]^-$	$[CA-H-CO_2]^-$
	m/z→	601.12	557.13	515.12	497.11	439.09	395.10	377.09	353.09	335.08	233.07	191.06	179.03	173.04	161.02	155.03	137.02	135.04
**4**	15	13.9	9.5	6.6	0.13	15.5	28.4	2.3	1.4	0.38	14.7	0.41	054	0.11	0.45	-	-	-
	25	-	0.30	0.74	-	0.98	11.1	1.0	3.9	1.2	65.6	1.4	1.9	0.70	1.6	-	-	0.16
	30	-	-	-	-	-	2.4	0.59	2.9	1.2	68.0	3.5	2.7	1.6	2.2	0.13	0.34	0.54
	35	-	-	-	-	-	0.41	0.28	1.3	1.1	60.9	4.9	3.3	3.3	3.0	0.37	0.24	0.73
	45	-	-	-	-	-	-	-	0.13	0.44	30.4	7.7	4.4	8.0	4.4	1.0	0.50	2.2
**7**	15	14.5	6.8	3.9	-	8.7	35.5	2.5	0.87	0.17	18.8	0.18	0.80	0.29	0.47	-	0.087	-
	25	0.10	-	0.51	-	0.59	10.0	0.79	2.2	0.43	69.0	0.92	2.3	2.8	1.5	0.20	0.16	0.23
	30	-	-	-	-	0.13	2.5	0.40	1.8	0.58	68.0	1.9	3.4	6.8	2.0	0.51	0.14	0.48
	35	-	-	-	-	-	0.44	0.23	0.77	0.55	59.1	3.5	4.1	15.2	2.3	0.94	0.52	1.2
	45	-	-	-	-	-	-	-	-	0.13	28.0	4.3	3.9	38.7	2.8	2.0	1.2	2.3
**5**	15	2.0	20.2	9.7	2.5	0.23	46.4	0.48	5.1	1.4	0.086	0.061	2.8	0.11	0.20	-	-	0.20
	25	-	0.26	0.56	1.2	-	35.2	1.1	12.8	11.1	1.4	0.85	18.5	0.61	1.1	0.96	-	1.7
	30	-	-	-	0.33	-	11.7	0.62	9.0	12.4	12.1	2.3	28.9	2.1	2.1	1.2	0.15	3.0
	35	-	-	-	-	-	1.8	0.23	3.4	5.6	5.4	2.8	24.6	2.3	1.6	0.97	0.23	3.4
	45	-	-	-	-	-	-	0.12	0.93	3.4	1.5	6.9	42.6	4.3	5.4	1.4	0.80	13.8
**6**	15	2.8	4.1	5.8	-	12.1	58.7	0.57	7.5	0.29	5.2	0.19	0.67	0.33	0.30	-	-	0.19
	25	-	-	0.46	-	0.71	32.3	0.10	15.2	1.2	36.4	2.1	2.8	3.3	0.71	0.27	-	0.43
	30	-	-	-	-	-	11.2	-	11.2	1.2	48.5	5.5	5.8	9.7	1.1	0.52	0.21	0.91
	35	-	-	-	-	-	2.7	-	6.5	1.1	40.9	10.0	10.1	18.7	1.9	1.1	0.38	2.0
	45	-	-	-	-	-	-	-	0.77	0.52	14.5	13.6	12.7	39.0	2.9	2.2	1.1	4.2

[a] The numbers correspond to 1,5-dicaffeoyl-3-malonylquinic acid (**4**), 3,5-dicaffeoyl-1-malonylquinic acid (**5**), 3,5-dicaffeoyl-4-malonyl-*epi*-quinic acid (**6**), and 1,5-dicaffeoyl-4-malonylquinic acid (**7**). [b] Fragmentations of deprotonated molecules were analyzed by parallel reaction monitoring (PRM) method using collision-induced dissociation (CID) energies of 15 eV, 25 eV, 30 eV, 35 eV, and 45 eV. [c] Expressed as percentages of the total fragment ion abundance. [e] The structures of characteristic ions (CIs) were identified based on their *m/z* values obtained through HR-MS/MS, enabling the calculation of their molecular formulas. The abbreviations in the structures of the CIs correspond to caffeic acid (CA), malonic acid (MA), and quinic acid (QA). The differences between the calculated and measured masses of the CIs were within 5.0 ppm. Note: The measured *m/z* values of the CIs are reported to two decimal places due to space limitations. The HR-MS2 spectra of MDiCQAs **4**–**7**, acquired at CID energies of 25 eV and 45 eV, are presented in Figures S10–14 of the Supporting Information.

Table S4.
Relative Abundance of Characteristic Ions in the Tandem Mass Spectra (MS2) of 1,5-Dicaffeoyl-3,4-Dimalonylquinic Acid **9**, Determined by HPLC High-Resolution Orbitrap Tandem Mass Spectrometry Using Negative Electrospray Ionization (ESI) Mode.

No.[a]	CID[b] (eV)	Relative abundances (%)[c] of characteristic ions (CIs)[e]																					
	CI→	[M-H]$^-$	[M-H-CO_2]$^-$	[M-H-MA]$^-$	[M-H-MA-CO_2]$^-$	[M-H-CA]$^-$	[M-H-CA-CO_2]$^-$	[M-H-CA-CO_2-H_2O]$^-$	[M-H-CA-MA]$^-$	[M-H-CA-2×CO_2]$^-$	[M-H-CA-2×CO_2-H_2O]$^-$	[M-H-CA-MA-CO_2]$^-$	[M-H-CA-MA-CO_2-H_2O]$^-$	[M-H-2×CA-CO_2]$^-$	[M-H-2×CA-2×CO_2]$^-$	[M-H-2×CA-MA-CO_2]$^-$	[M-H-2×CA-MA-CO_2-H_2O]$^-$	[QA-H]$^-$	[CA-H]$^-$	[QA-H-H_2O]$^-$	[CA-H-H_2O]$^-$	[QA-H-2×H_2O]$^-$	[CA-H-CO_2]$^-$
	m/z→	687.12	643.13	601.12	557.13	525.09	481.10	463.09	439.09	437.11	419.10	395.10	377.09	319.07	275.08	233.07	215.06	191.06	179.03	173.04	161.02	155.03	135.04
**9**	15	18.3	3.7	9.5	4.4	4.1	6.1	0.32	0.80	19.7	1.1	2.7	-	0.87	21.1	0.35	-	-	0.25	-	0.48	-	-
	30	-	-	-	-	-	0.22	-	0.26	0.84	0.21	4.8	0.33	0.97	67.3	13.3	1.2	-	2.5	0.19	1.4	-	0.53
	45	-	-	-	-	-	-	-	-	-	-	-	-	-	35.8	16.8	9.4	0.32	4.3	3.9	2.2	1.2	1.5

[a] The number corresponds to 1,5-dicaffeoyl-3,4-dimalonylquinic acid. [b] Fragmentation of the deprotonated molecule was analyzed by parallel reaction monitoring (PRM) method using collision-induced dissociation (CID) energies of 15 eV, 30 eV, and 45 eV. The corresponding HR-MS2 spectra, showing the detected ions, are presented in Figure S14 of the Supporting Information. [c] Expressed as percentages of the total fragment ion abundance. [e] The structures of characteristic ions (CIs) were identified based on their *m/z* values obtained through HR-MS/MS, enabling the calculation of their molecular formulas. The abbreviations in the structures of the CIs correspond to caffeic acid (CA), malonic acid (MA), and quinic acid (QA). The differences between the calculated and measured masses of the CIs were within 5.0 ppm. Note: The measured *m/z* values of the CIs are reported to two decimal places due to space limitations.

Table S5. Relative Abundance of Characteristic Ions in the Tandem Mass Spectra (MS2) of Flavonoid-Malonyl-Glucosides (FMGls) **1**–**3** and **8**, Determined by HPLC High-Resolution Orbitrap Tandem Mass Spectrometry Using Negative Electrospray Ionization (ESI) Mode.

No.[a]	CID[b] (eV)	Relative abundances (%)[c] of characteristic ions (CIs)[e]										
		negative ESI ionization mode							positive ESI ionization mode			
	CI→	[M-H]$^-$	[M-H-CO_2]$^-$	[M-H-2×CO_2]$^-$	[M-H-mal]$^-$	[M-H-mal-CO_2]$^-$	[M-H-mal-CO_2- H_2O]$^-$	[Agl-H]$^-$	[M+H]$^-$	[M+H-CO_2]$^-$	[M+H-mal]$^-$	[Agl+H]$^-$
**1**	*m/z*→	549.09	505.10	-	463.09	-	-	301.04	551.10	-	-	303.05
	10	1.5	88.0	-	0.52	-	-	1.1	28.3	-	-	60.0
	15	-	82.1	-	1.4	-	-	4.4	3.2	-	-	75.0
	20	-	61.8	-	2.5	-	-	14.5	0.18	-	-	62.5
	25	-	24.0	-	2.7	-	-	24.0	-	-	-	59.1
	30	-	7.6	-	2.2	-	-	26.9	-	-	-	56.7
	45	-	-	-	0.10	-	-	8.0	-	-	-	48.3
**2**	*m/z*→	533.09	489.10	-	447.09	-	-	285.04	535.11	491.12	449.11	287.05
	10	4.5	81.5	-	0.43	-	-	0.81	82.1	0.71	2.0	11.1
	12	1.36	82.8	-	0.59	-	-	1.0	69.5	1.0	3.3	20.2
	15	0.21	87.8	-	0.58	-	-	2.0	41.3	1.21	5.1	38.1
	20	-	87.5	-	0.75	-	-	7.5	14.1	0.49	4.6	76.7
	25	-	68.3	-	1.1	-	-	21.7	1.5	0.031	1.3	93.9
	30	-	44.4	-	1.1	-	-	49.6	0.10	-	0.17	96.3
	35	-	18.3	-	0.97	-	-	62.8	-	-	-	95.0
**3**	*m/z*→	533.09	489.10	-	447.09	-	-	285.04	535.11	-	449.11	287.05
	10	1.3	80.8	-	-	-	-	3.8	22.8	-	0.017	62.9
	12	0.32	82.0	-	0.14	-	-	7.4	10.4	-	0.05	70.8
	15	-	76.7	-	0.12	-	-	15.3	2.2	-	-	69.8
	20	-	43.3	-	0.14	-	-	39.4	0.10	-	-	62.0
	25	-	16.3	-	0.36	-	-	53.4	-	-	-	61.7
	30	-	3.6	-	0.22	-	-	51.4	-	-	-	53.3
	35	-	0.36	-	-	-	-	42.3	-	-	-	49.7
**8**	*m/z*→	619.09	575.10	531.11	533.09	489.10	471.09	285.04	621.11	577.12	535.11	287.05
	10	13.6	3.5	69.6	0.54	0.96	-	0.68	72.5	0.27	0.61	10.0
	12	5.3	1.7	75.4	0.37	1.3	-	0.80	67.2	0.39	1.3	18.5
	15	0.87	0.40	83.3	0.25	1.9	0.15	1.25	38.9	0.40	1.9	35.0
	20	-	-	82.1	-	2.6	0.26	3.0	10.7	0.18	1.7	64.2
	25	-	-	75.2	-	3.4	1.1	8.9	1.2	-	0.52	72.2
	30	-	-	58.1	-	4.0	3.0	24.0	-	-	0.12	81.6
	35	-	-	31.3	-	4.8	4.1	39.1	-	-	-	75.0

[a] The numbers correspond to quercetin-3-*O*-(6''-*O*-malonyl)-β-D-glucoside (**1**), luteolin-7-*O*-(6''-*O*-malonyl)-β-D-glucoside (**2**), kaempferol-3-*O*-(6''-*O*-malonyl)-β-D-glucoside (**3**), and luteolin-7-*O*-(2'',6''-di-O-malonyl)-β-D-glucoside (**8**). [b] The fragmentations of deprotonated and protonated molecules were analyzed using the parallel reaction monitoring (PRM) method with collision-induced dissociation (CID) energies ranging from 10 eV to 45 eV. [c] Expressed as percentages of the total fragment ion abundance. [e] The structures of characteristic ions (CIs) were identified based on their *m/z* values obtained through HR-MS/MS, enabling the calculation of their molecular formulas. The differences between the calculated and measured masses of the CIs were within 5.0 ppm. Note: The measured *m/z* values of the CIs are reported to two decimal places due to space limitations.

Table S6.
Composition of Dried Leaf, Inflorescence, and Root Samples of *Anthriscus cerefolium* Collected at the Beginning of the Vegetation Cycle and in the Flowering Stage, Determined by HPLC-UV-MS.

Tissue	Coll. time[a]	Coll. locat.[b]	Sample name[c]	Amounts of compounds in the dried tissues (mg/g)		
				**4**	**7**	**9**
Leaf	BVC	1	AC-L-BVC-1*A*	3.17	0.80	6.06
			AC-L-BVC-1*B*	3.18	0.84	6.18
		2	AC-L-BVC-2	2.68	0.85	4.33
		3	AC-L-BVC-3	3.36	1.14	2.84
		4	AC-L-BVC-4	3.14	0.83	6.34
	FS	1	AC-L-FS-1*A*	6.20	1.27	0.66
			AC-L-FS-1*B*	6.33	1.31	0.65
			AC-L-FS-1*C*	6.26	1.26	0.64
		6	AC-L-FS-6*A*	10.08	1.93	1.30
			AC-L-FS-6*B*	10.64	2.00	1.34
		7	AC-L-FS-7	5.12	1.17	0.58
		8	AC-L-FS-8	11.58	2.43	1.28
Inflorescence	FS	1	AC-I-FS-1*A*	10.65	2.07	-
			AC-I-FS-1*B*	10.85	2.11	-
		5	AC-I-FS-5*A*	14.75	2.79	0.51
			AC-I-FS-5*B*	14.98	2.88	0.51
		6	AC-I-FS-6*A*	28.60	4.62	1.01
			AC-I-FS-6*B*	28.53	4.64	1.00
		7	AC-I-FS-7	11.81	1.86	0.19
Root	BVC	1	AC-R-BVC-1	0.42	0.46	0.22
		2	AC-R-BVC-2	0.60	0.43	0.30
		3	AC-R-BVC-3	0.51	0.64	0.23
	FS	7	AC-R-FS-7	0.30	0.27	0.09
		9	AC-R-FS-9	0.37	0.29	0.13

[a] Collection time: Samples were collected at the beginning of the vegetation cycle (in January and February) (BVC samples) and in the flowering stage (April) (FS samples) of *Anthriscus cerefolium*.
[b] Sample collection location: plant samples were collected from different Hungarian locations marked with numbers 1–9.
[c] Sample name abbreviations refer to the plant species (AC - *Anthriscus cerefolium*), analyzed tissues (L - leaf, I - Inflorescence, or R - root), collection times (BVC - beginning of the vegetation cycle, or FS - flowering stage), and independent extraction parallels (A–C, printed in italic).

Table S7. Composition of Dried Leaf, Inflorescence, and Root Samples of *Chaerophyllum bulbosum* Collected at the Beginning of the Vegetation Cycle and in the Flowering Stage, Determined by HPLC-UV-MS.

Tissue	Coll. time[a]	Coll. locat.[b]	Sample name[c]	Amounts of compounds in the dried tissues (mg/g)						
				MDiCQAs[d]			FMGls[e]			
				**4**	**7**	**9**	**1**	**2**	**3**	**8**
Leaf	BVC	1	CB-L-BVC-1	7.57	1.49	0.81	-	6.27	-	4.39
		2	CB-L-BVC-2	7.40	1.55	0.91	-	8.35	-	2.69
		3	CB-L-BVC-3*A*	6.89	1.46	0.66	-	6.45	-	3.45
			CB-L-BVC-3*B*	6.96	1.45	0.65	-	6.55	-	3.46
		4	CB-L-BVC-4	8.64	1.76	1.18	-	10.64	-	2.42
		5	CB-L-BVC-5	5.86	1.56	0.74	-	4.95	-	3.35
	FS	1	CB-L-FS-1	2.04	0.50	0.17	0.34	1.68	-	-
		2	CB-L-FS-2	2.75	0.64	0.83	1.26	4.99	-	-
		3	CB-L-FS-3	1.77	0.54	0.76	1.89	2.89	-	-
		4	CB-L-FS-4*A*	4.01	0.89	1.02	1.54	3.35	-	0.38
			CB-L-FS-4*B*	3.96	0.89	1.05	1.47	3.22	-	0.39
		5	CB-L-FS-5*A*	4.62	0.99	1.59	2.73	5.63	-	0.76
			CB-L-FS-5*B*	4.98	1.06	1.64	2.96	5.78	-	0.75
		6	CB-L-FS-6*A*	3.95	0.89	1.79	0.35	1.63	-	-
			CB-L-FS-6*B*	4.17	0.90	1.87	0.36	1.65	-	-
			CB-L-FS-6*C*	3.87	0.86	1.65	0.34	1.52	-	-
		7	CB-L-FS-7	4.57	1.20	2.54	0.36	1.81	-	-
Inflorescence	FS	1	CB-I-FS-1	8.73	1.67	5.71	6.90	0.61	1.49	-
		2	CB-I-FS-2	8.78	2.02	10.00	10.43	0.72	2.40	-
		4	CB-I-FS-4*A*	9.87	2.19	15.03	16.33	1.12	5.54	0.39
			CB-I-FS-4*B*	8.93	2.07	13.55	14.86	0.98	5.03	0.37
		5	CB-I-FS-5*A*	20.22	4.57	30.31	25.61	1.92	9.98	0.79
			CB-I-FS-5*B*	20.29	4.56	30.97	25.87	1.84	9.66	0.82
			CB-I-FS-5*C*	18.46	4.35	27.82	22.63	1.95	9.29	0.76
		6	CB-I-FS-6*A*	9.33	1.68	13.87	5.19	0.52	2.95	-
			CB-I-FS-6*B*	9.06	1.62	13.65	5.32	0.54	2.84	-
			CB-I-FS-6*C*	8.73	1.63	13.35	4.91	0.47	2.83	-
			CB-I-FS-6*D*	8.80	1.65	13.32	5.01	0.50	2.80	-
		7	CB-I-FS-7	7.71	1.74	24.32	6.34	0.70	3.89	-
Root	BVC	1	CB-R-BVC-1*A*	0.49	0.31	0.45	-	-	-	-
			CB-R-BVC-1*B*	0.53	0.32	0.36	-	-	-	-
			CB-R-BVC-1*C*	0.53	0.32	0.43	-	-	-	-
		2	CB-R-BVC-2	0.42	0.30	0.27	-	-	-	-
		3	CB-R-BVC-3*A*	0.74	0.34	0.47	-	-	-	-
			CB-R-BVC-3*B*	0.72	0.34	0.46	-	-	-	-
		4	CB-R-BVC-4	1.07	0.42	0.33	-	-	-	-
		5	CB-R-BVC-5	1.34	0.49	0.22	-	-	-	-
	FS	2	CB-R-FS-2	0.40	0.28	0.00	-	-	-	-
		4	CB-R-FS-4*A*	0.82	0.36	0.12	-	-	-	-
			CB-R-FS-4*B*	0.85	0.36	0.13	-	-	-	-
		7	CB-R-FS-7	0.36	-	-	-	-	-	-

[a] Collection time: Samples were collected at the beginning of the vegetation cycle (in March and April) (BVC samples) and in the flowering stage (June) (FS samples) of *Chaerophyllum bulbosum*.
[b] Sample collection location: plant samples were collected from different Hungarian locations marked with numbers 1–7.
[c] Sample name abbreviations refer to the plant species (CB - *Chaerophyllum bulbosum*), analyzed tissue (L - leaf, I - Inflorescence, or R - root), collection time (BVC - beginning of the vegetation cycle, or FS - flowering stage), and independent extraction parallels (A–D printed in italic).
[d, e] Malonyl-dicaffeoylquinic acids (MDiCQAs) and flavonoid-malonyl-glucosides (FMGls) were determined in the samples.

Table S8.
Composition of Dried Leaf, Inflorescence, and Root Samples of *Anthriscus sylvestris* Collected at the Beginning of the Vegetation Cycle and in the Flowering Stage, Determined by HPLC-UV-MS.

Tissue	Coll. time[a]	Coll. locat.[b]	Sample name[c]	Amounts of compounds in the dried tissues (mg/g)[d]		
				**4**	**5**	**6**
Leaf	BVC	1	AS-L-BVC-1	1.79	2.08	1.91
		2	AS-L-BVC-2	3.29	1.20	0.78
		5	AS-L-BVC-5*A*	1.98	1.25	0.79
		5	AS-L-BVC-5*B*	1.93	1.20	0.81
	FS	1	AS-L-FS-1	2.47	-	-
		2	AS-L-FS-2	1.45	0.12	0.23
		4	AS-L-FS-4	2.64	-	-
Inflores-cence	FS	1	AS-I-FS-1	6.70	-	-
		2	AS-I-FS-2	2.85	0.36	0.28
		3	AS-I-FS-3	2.93	0.62	0.57
		4	AS-I-FS-4	2.46	0.18	0.17
Root	BVC	1	AS-R-BVC-1	1.80	0.59	0.10
		5	AS-R-BVC-5	1.32	0.28	0.15
	FS	1	AS-R-FS-1	2.71	0.22	0.11
		3	AS-R-FS-3	3.52	0.22	0.10

[a] Collection time: Samples were collected at the beginning of the vegetation cycle (from January to March) (BVC samples) and in the flowering stage (May) (FS samples) of *Anthriscus sylvestris*.
[b] Sample collection location: plant samples were collected from different Hungarian locations marked with numbers 1–5.
[c] Sample name abbreviations refer to the plant species (AS - *Anthriscus sylvestris*), analyzed tissue (L - leaf, I - Inflorescence, or R - root), collection time (BVC - beginning of the vegetation cycle, or FS - flowering stage), and independent extraction parallel (A and B printed in italic).
[d] The HPLC separations were performed by the HPLC gradient programs 1 and 2. Because compounds **5** and **6** were co-eluting by the HPLC gradient program 1 used in this study to quantify compounds, only their total amounts and the amounts of compound **4** could be determined using program 1. To separate compounds **5** and **6**, gradient program 2 was developed. Considering the peak area ratios between compounds **5** and **6** obtained by the gradient programs 2, and their total amount determined by the gradient program 1, the amounts of compounds **5** and **6** could also be calculated.

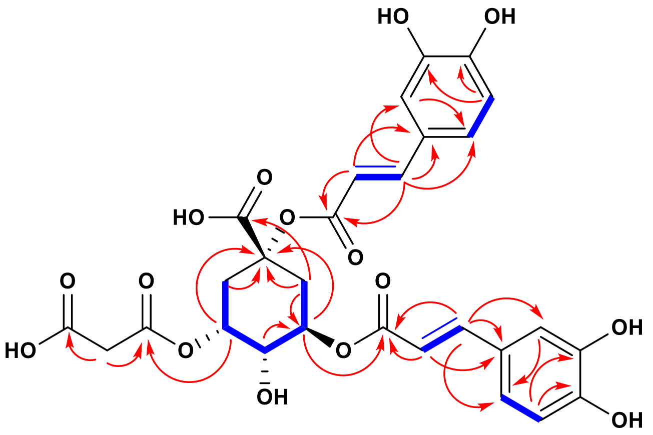

4

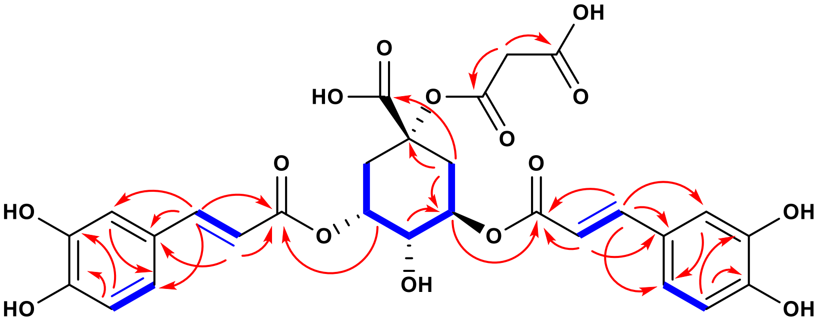

5

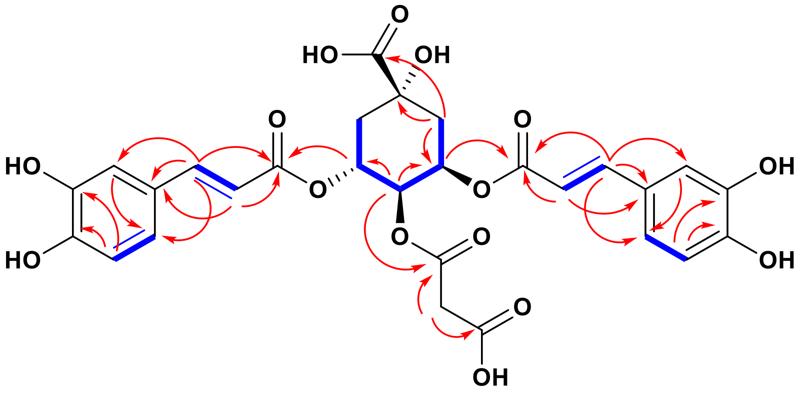

6

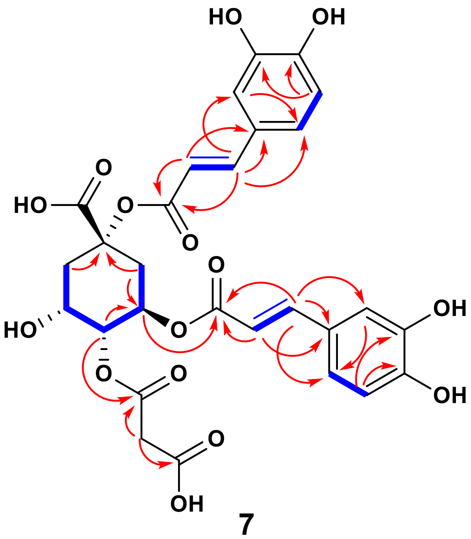

7

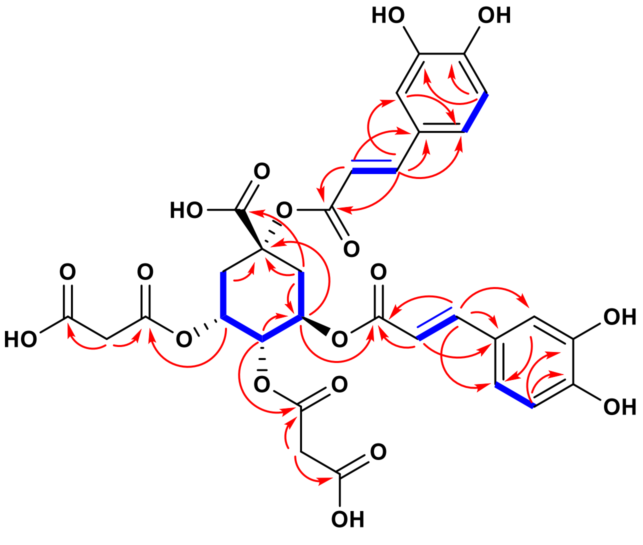

9

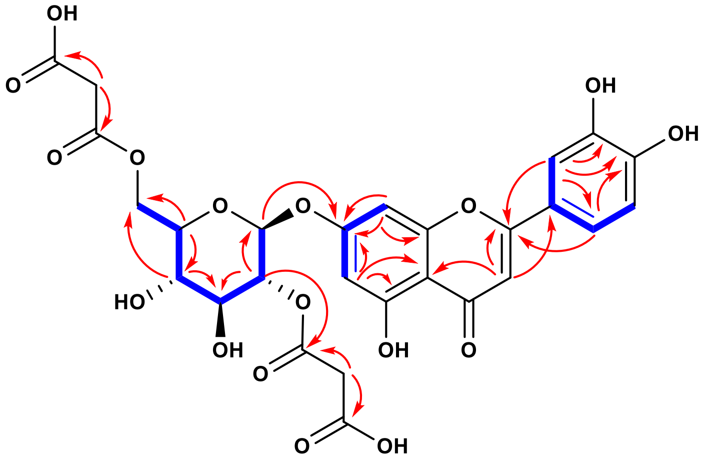

8

(**a**)

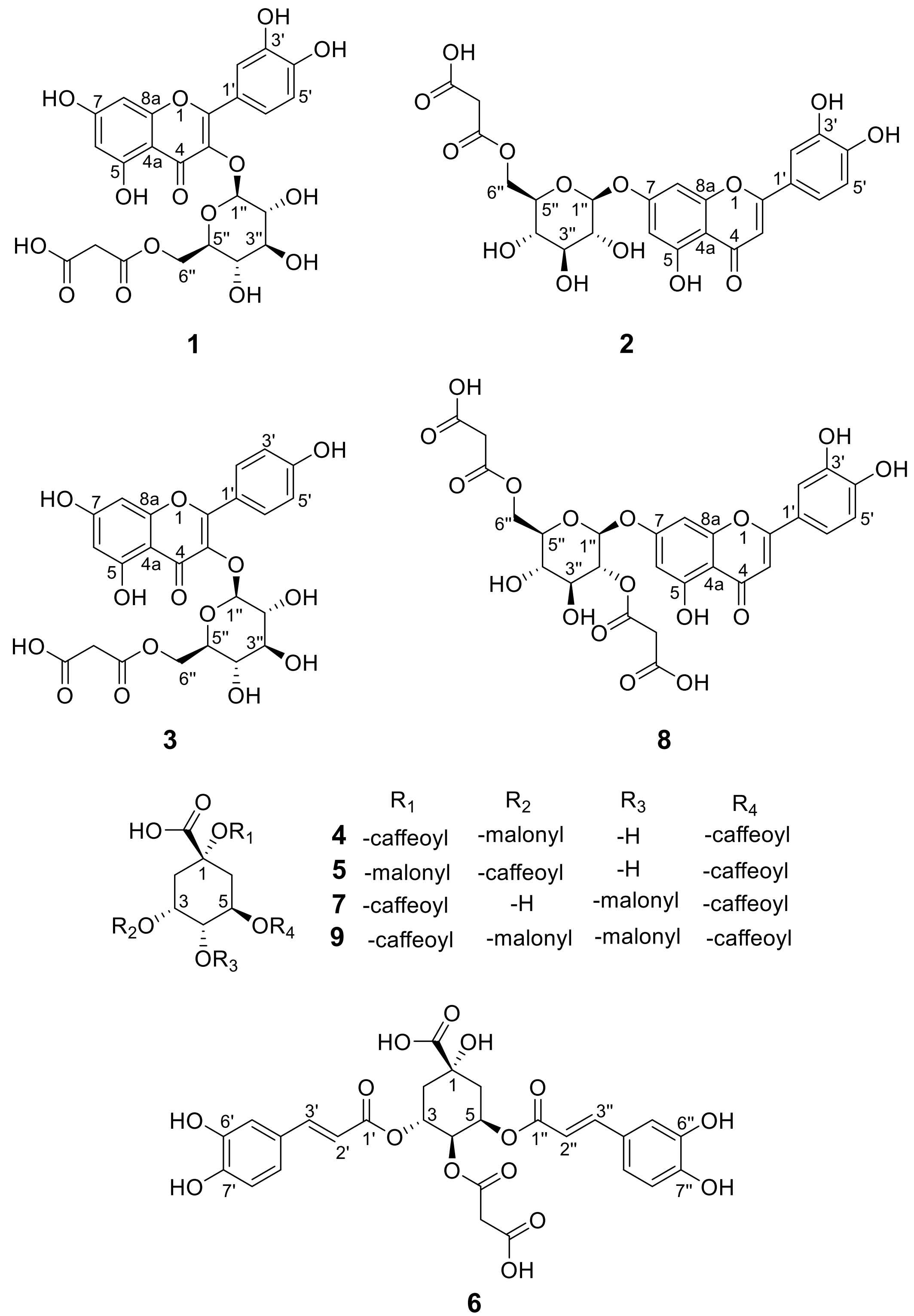

(**b**)

Figure S15. (**a**) Key 2D-NMR correlations (^{1}H–^{1}H COSY; blue, ^{1}H–^{13}C HMBC; red arrow) of isolated compounds **4**–**9**. (**b**) Chemical structures of isolated compounds **1**–**9**.

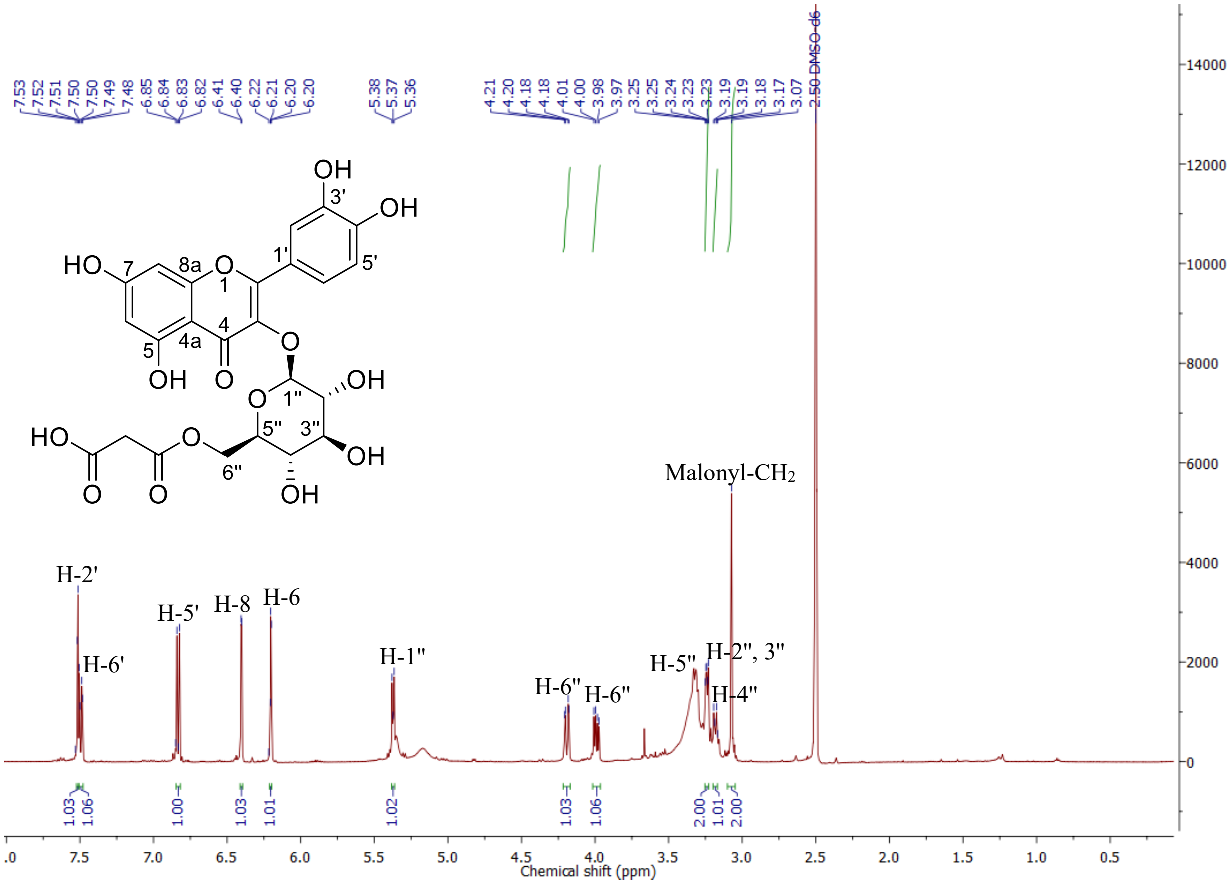

Figure S16. ^{1}H NMR (DMSO-d_6, 500 MHz) spectrum of compound **1**.

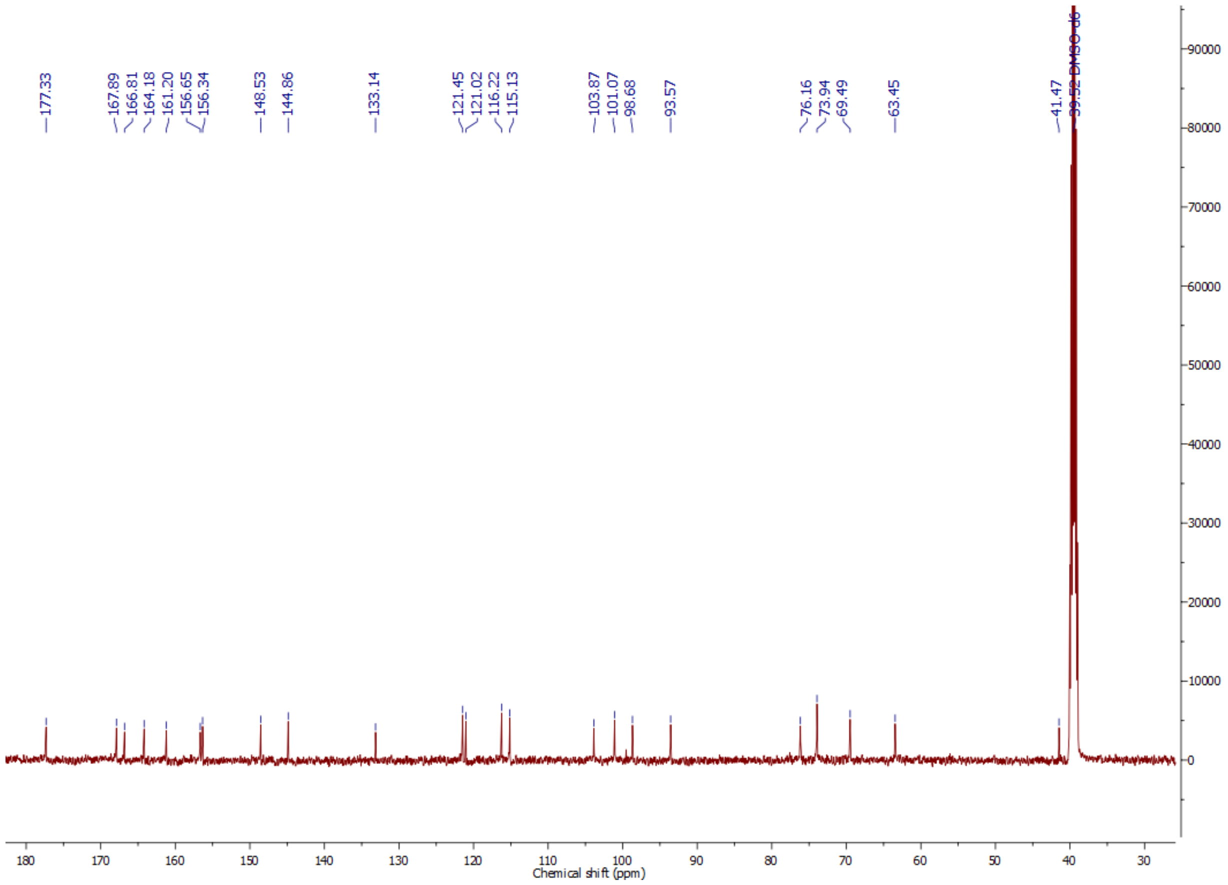

Figure S17. ^{13}C NMR (DMSO-d_6, 125 MHz) spectrum of compound **1**.

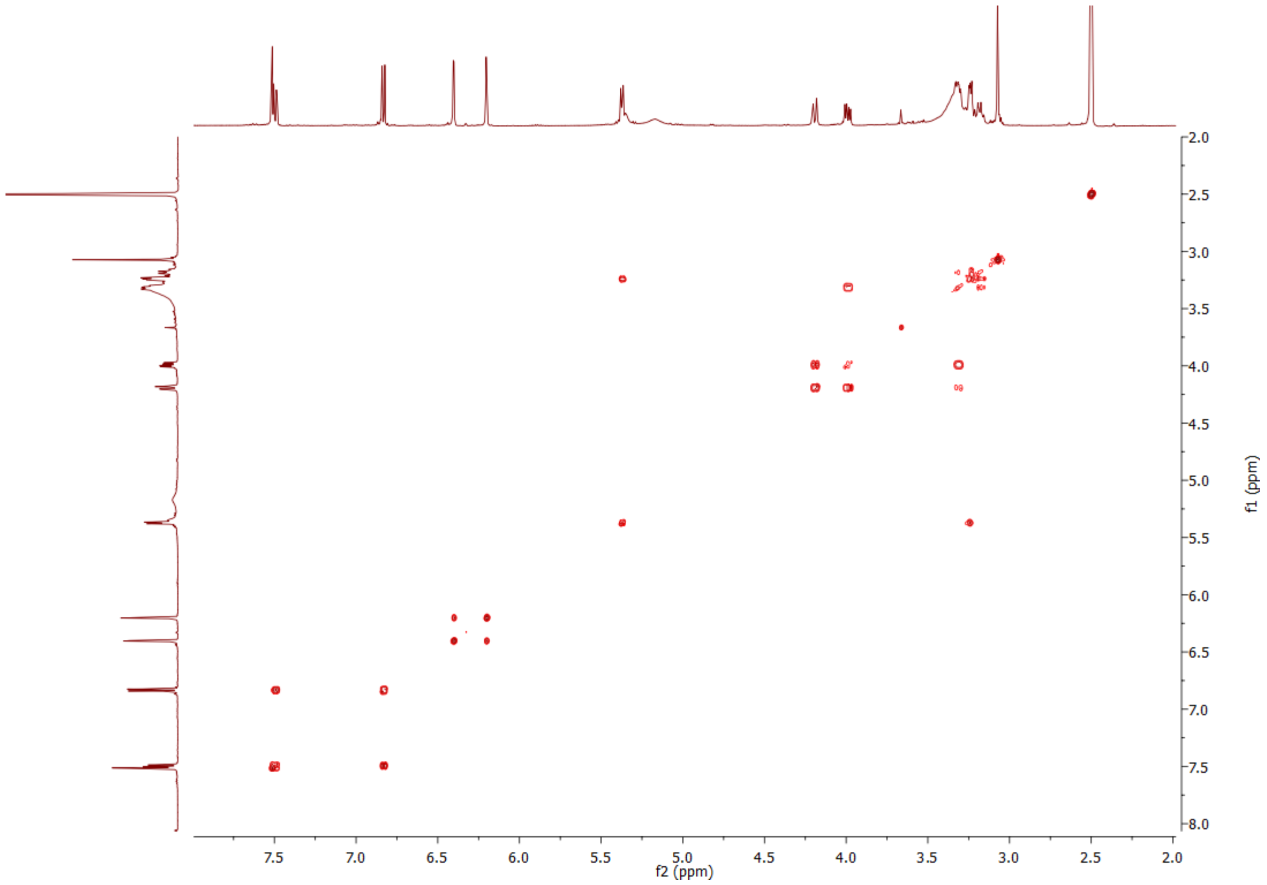

Figure S18. ^{1}H-^{1}H COSY (DMSO-d_6, 500 MHz) spectrum of compound **1**.

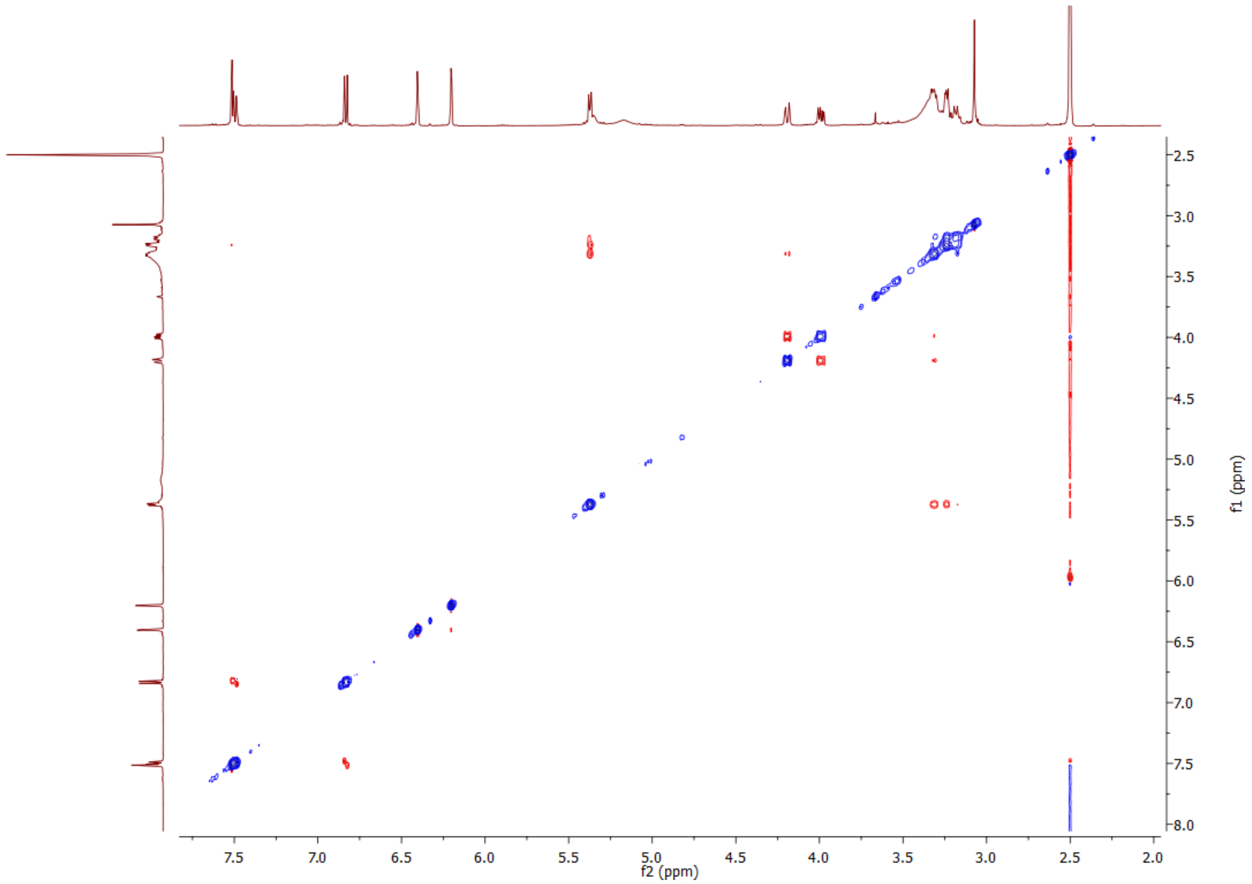

Figure S19. ^{1}H-^{1}H ROESY (DMSO-d_6, 500 MHz) spectrum of compound **1**.

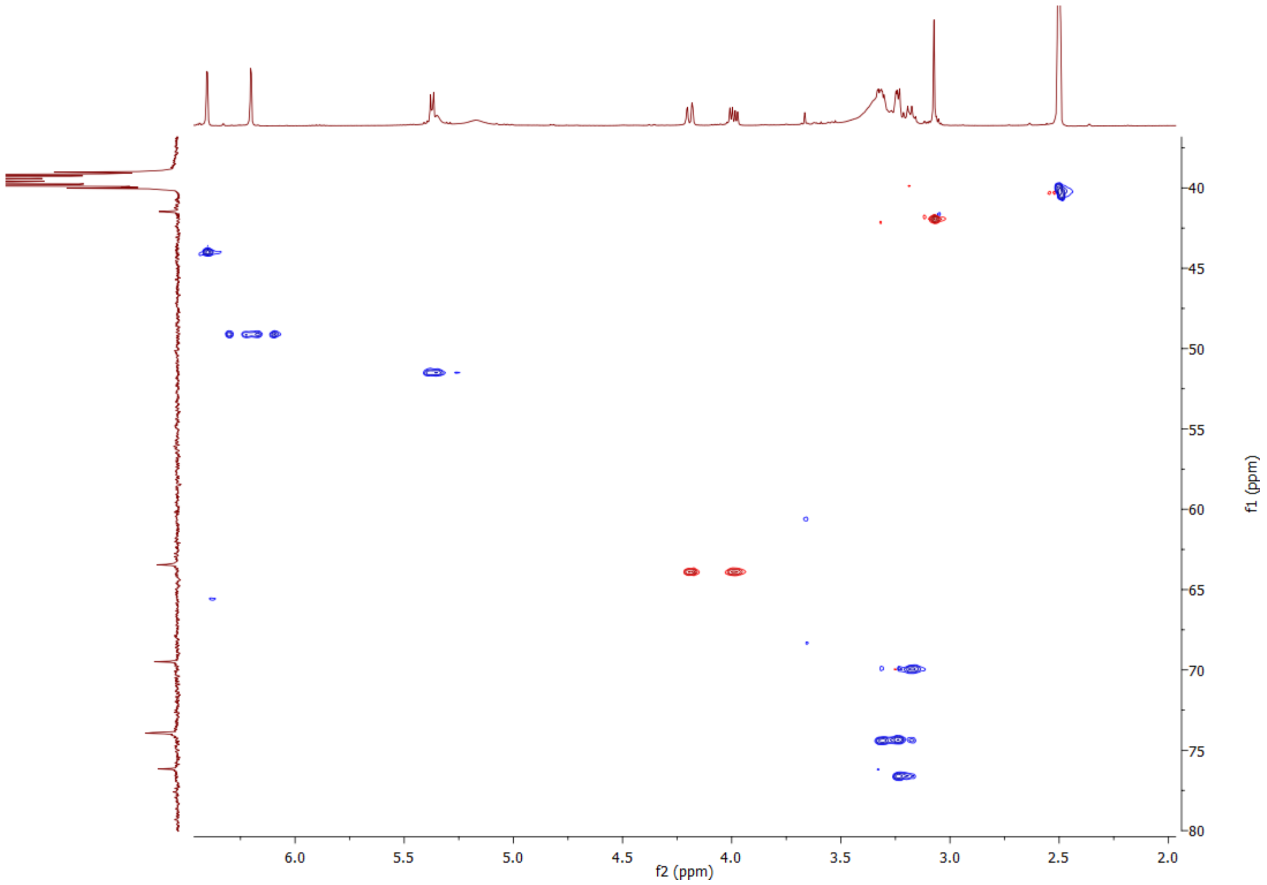

Figure S20. ^{1}H–^{13}C HSQC (DMSO-d_6, 500/125 MHz) spectrum of compound **1** for aliphatic region.

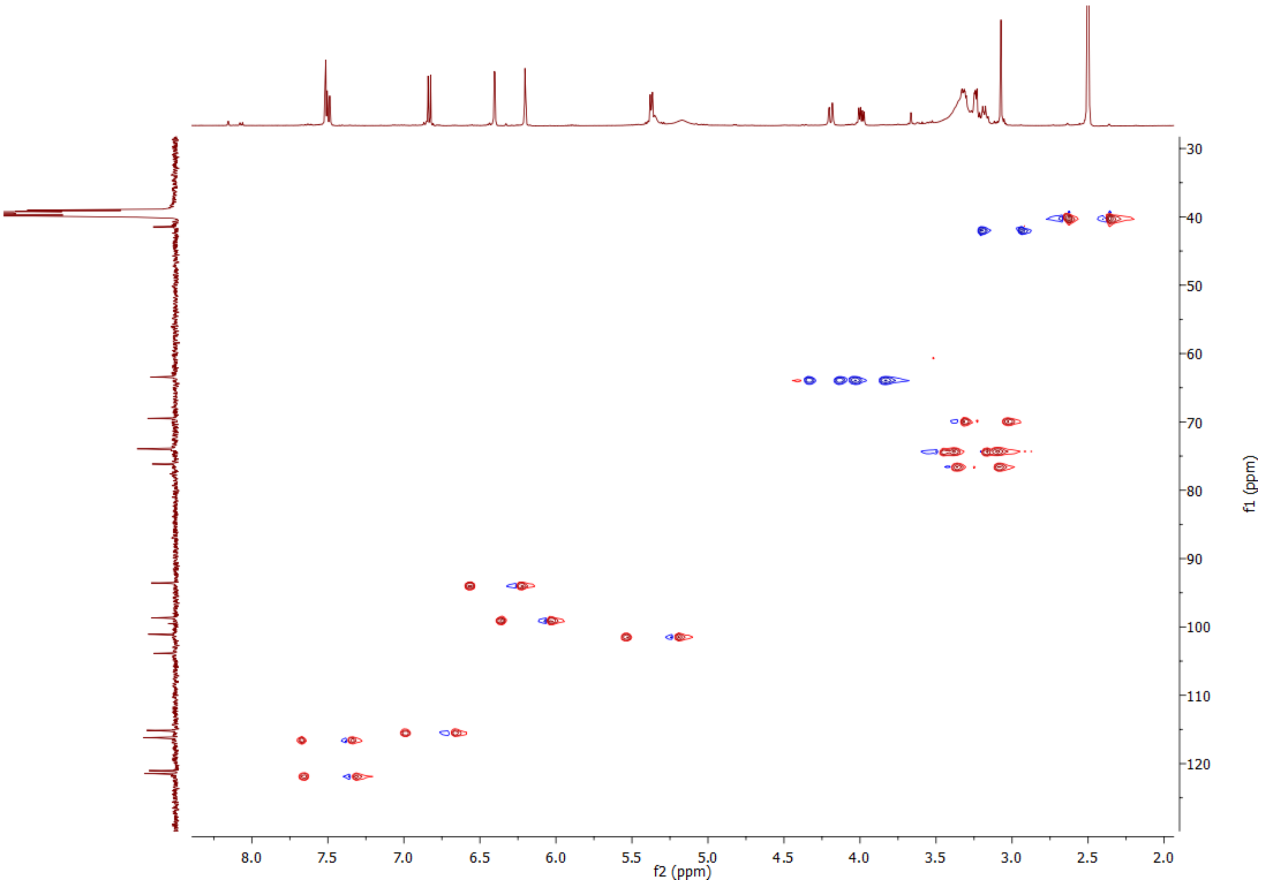

Figure S21. ^{1}H–^{13}C HSQC (DMSO-d_6, 500/125 MHz) spectrum (not decoupled) of compound **1**.

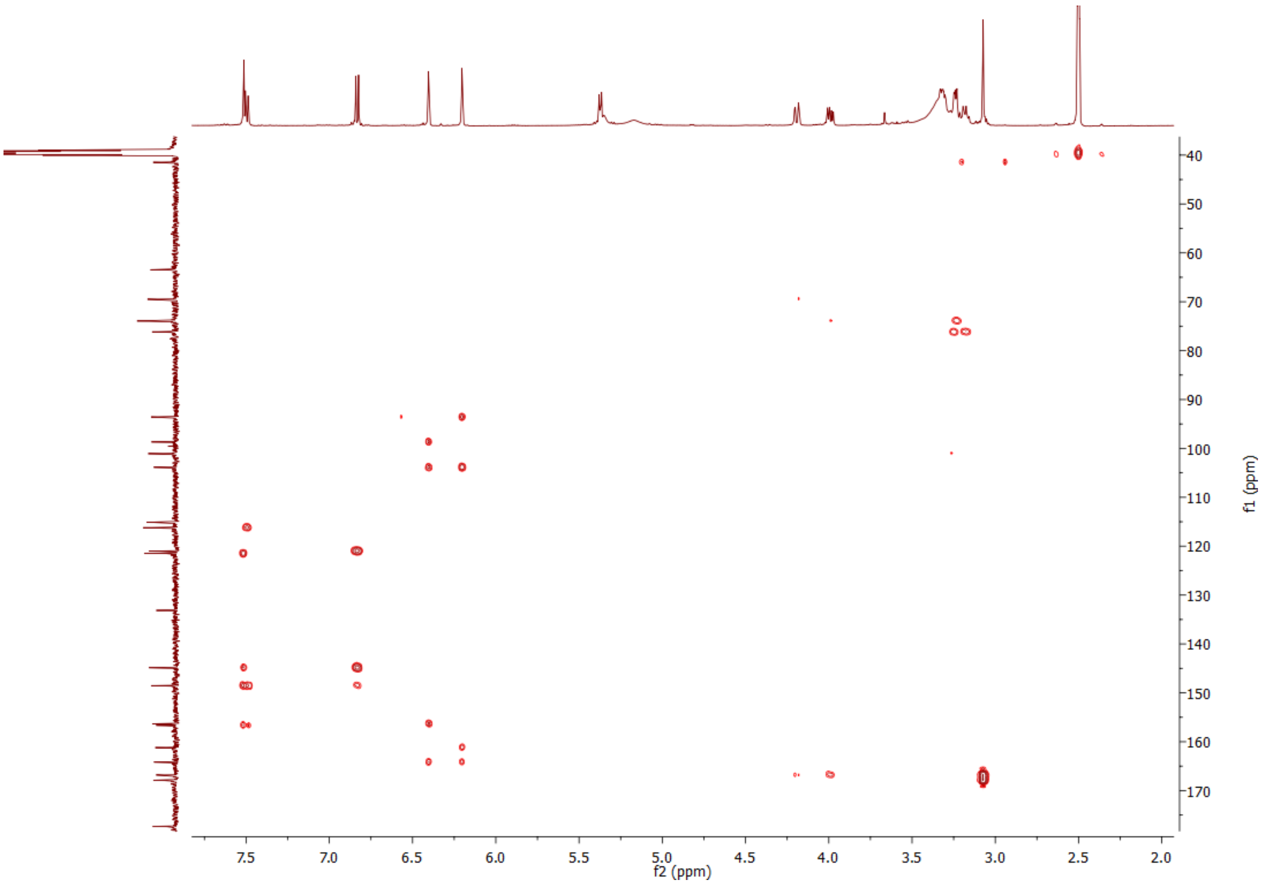

Figure S22. ^{1}H–^{13}C HMBC (DMSO-d_6, 500/125 MHz) spectrum of compound **1**.

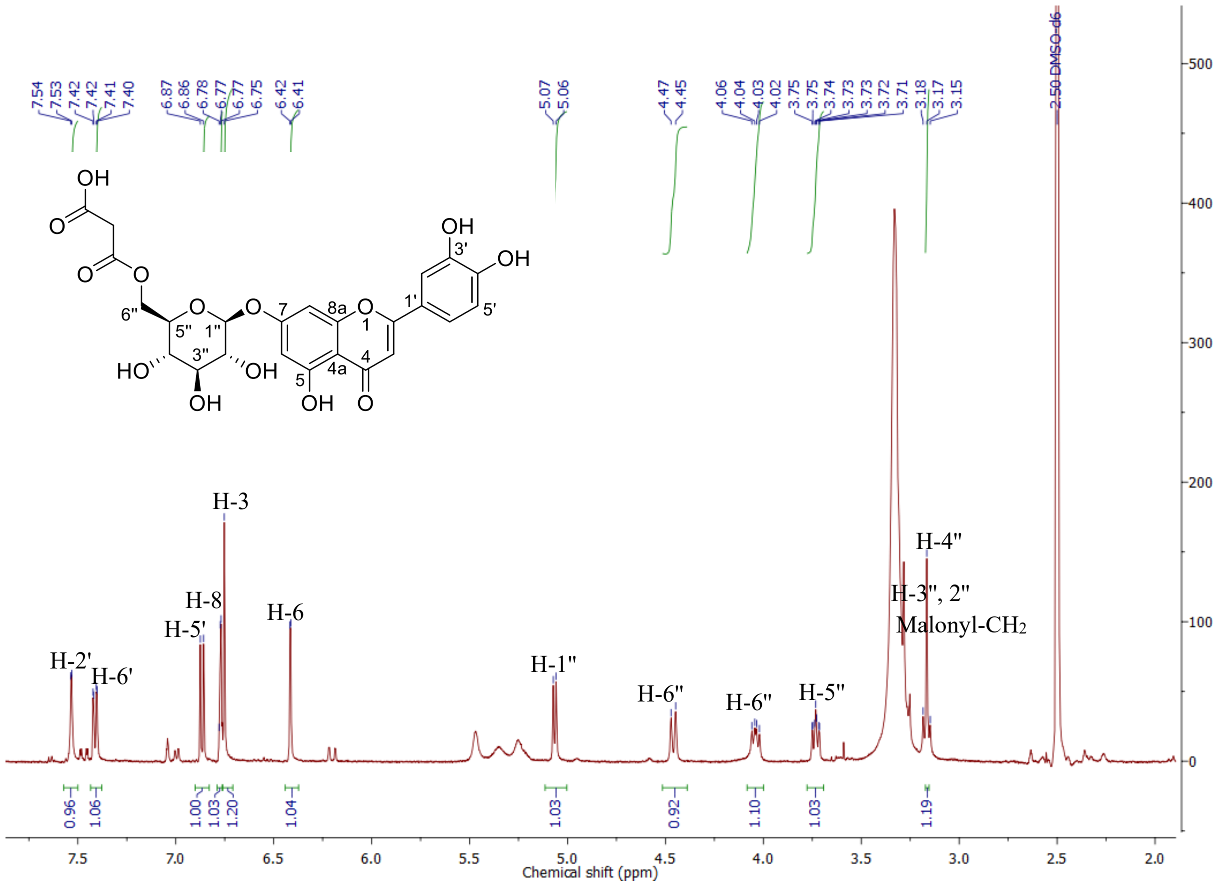

Figure S23. ^{1}H NMR (DMSO-d_6, 500 MHz) spectrum of compound **2.**

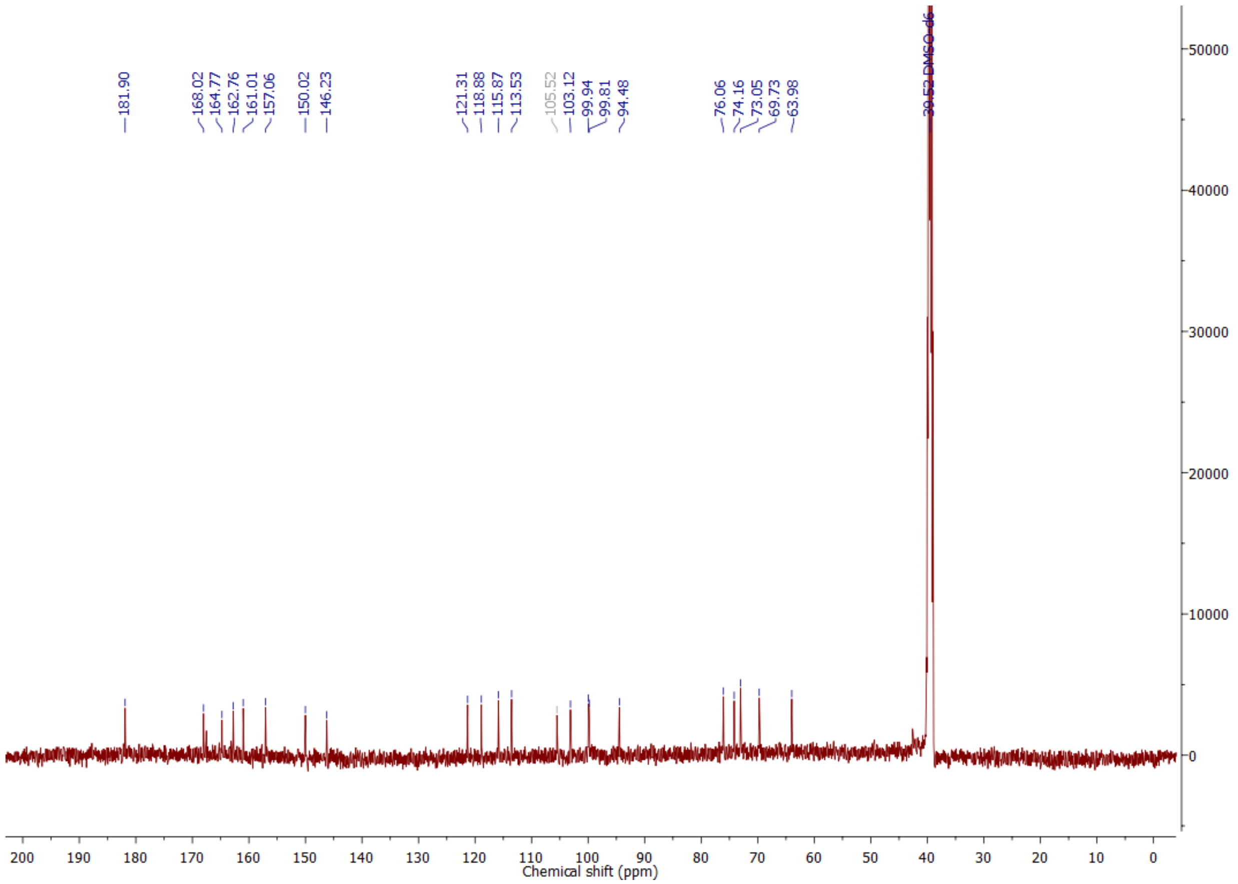

Figure S24. ^{13}C NMR (DMSO-d_6, 125 MHz) spectrum of compound **2**.

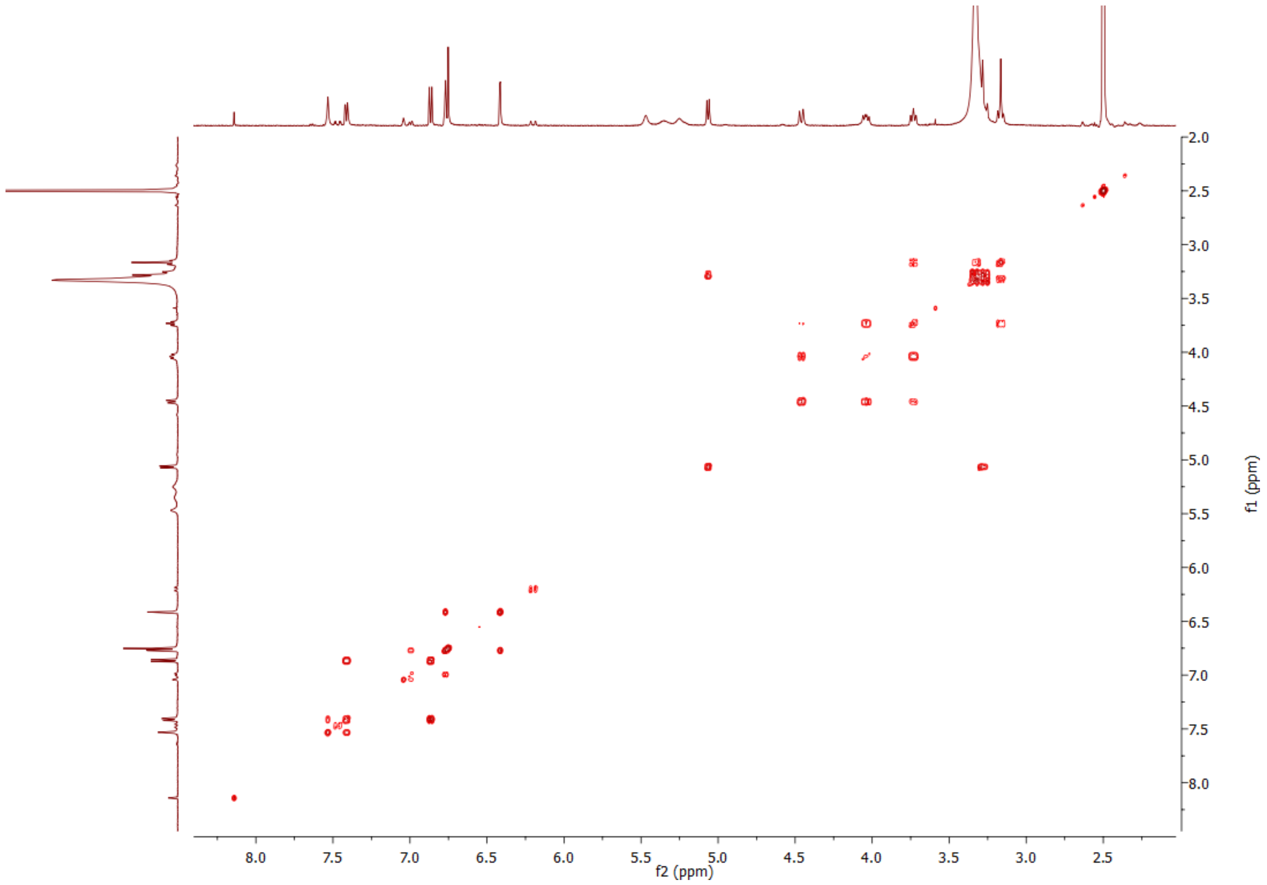

Figure S25. ^{1}H-^{1}H COSY (DMSO-d_6, 500 MHz) spectrum of compound **2**.

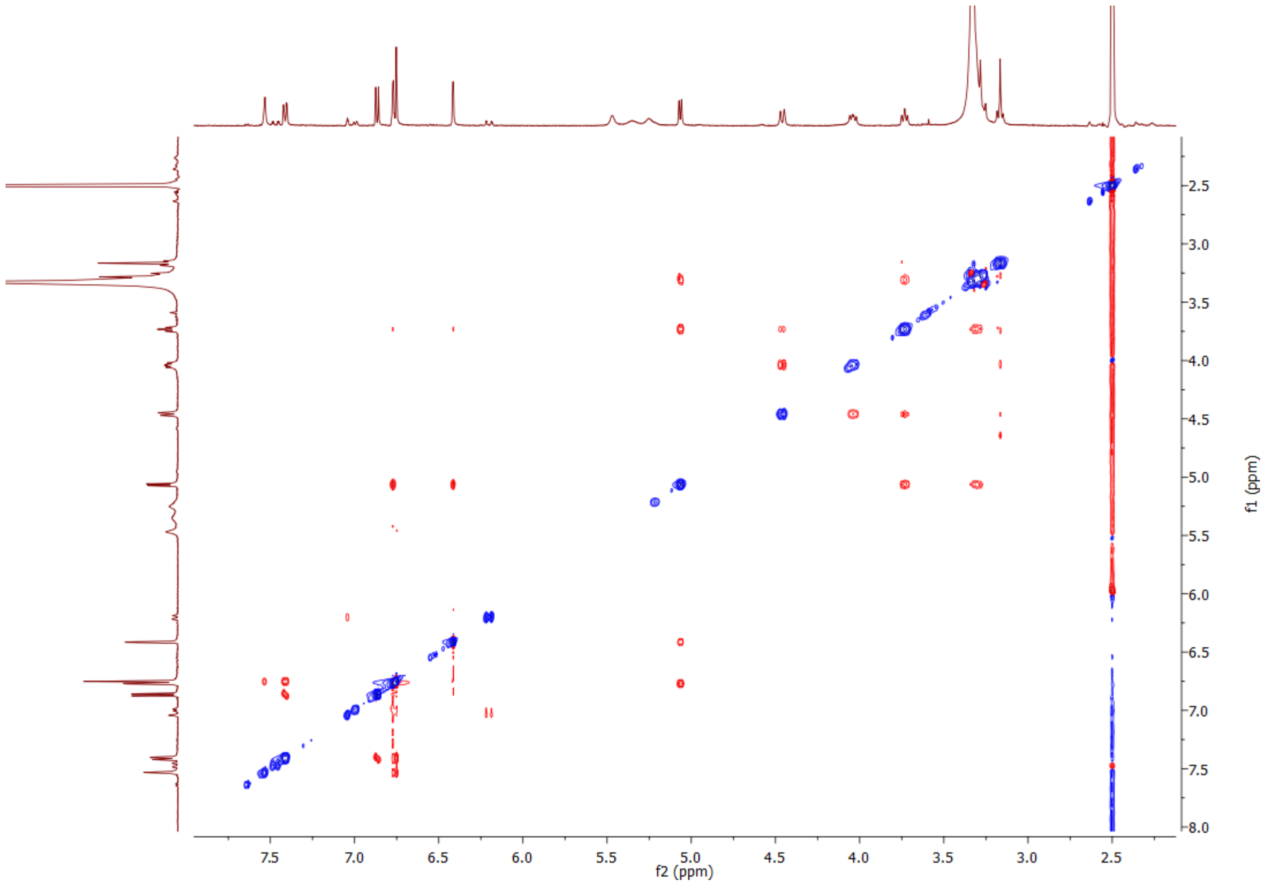

Figure S26. ^{1}H-^{1}H ROESY (DMSO-d_6, 500 MHz) spectrum of compound **2**.

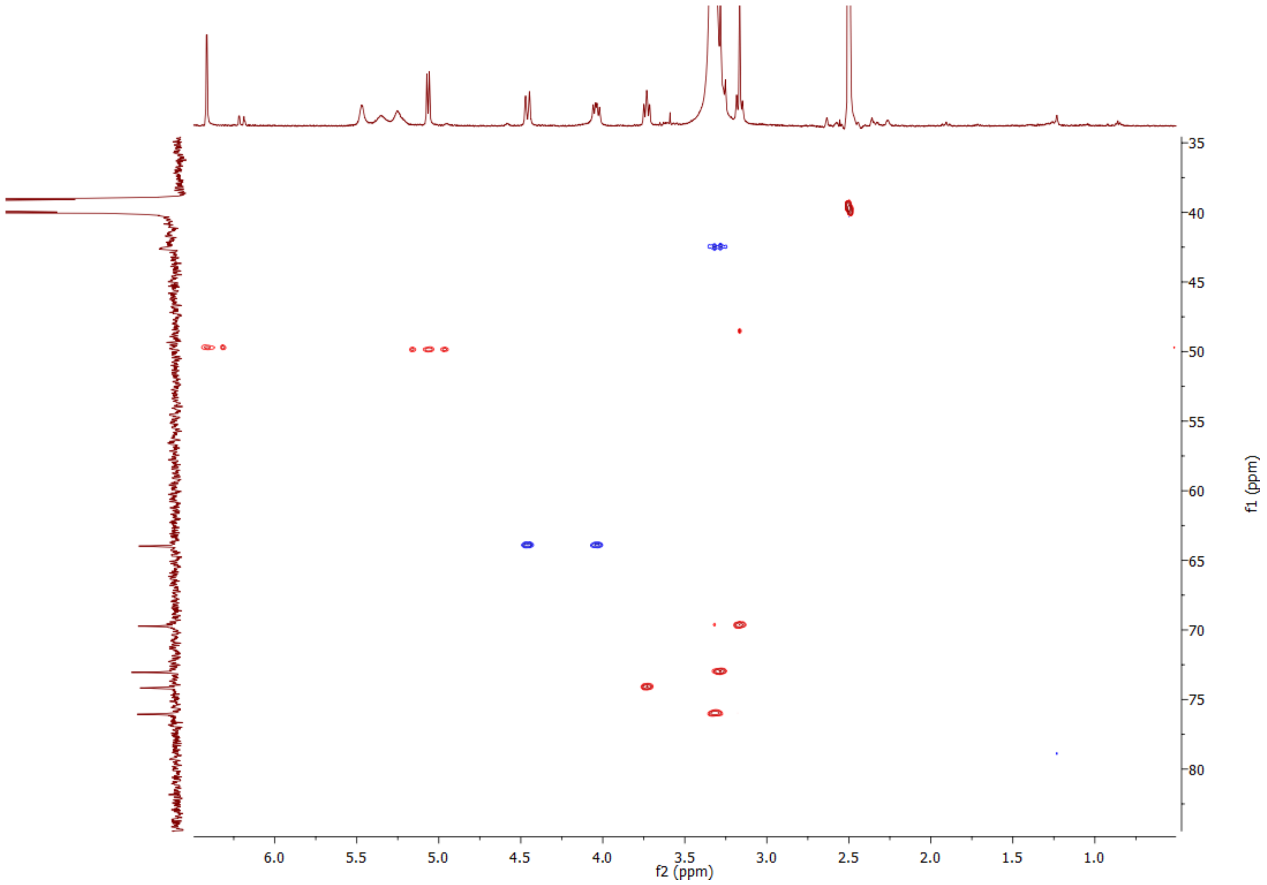

Figure S27. ^{1}H-^{13}C HSQC (DMSO-d_6, 500/125 MHz) spectrum of compound **2** for aliphatic region.

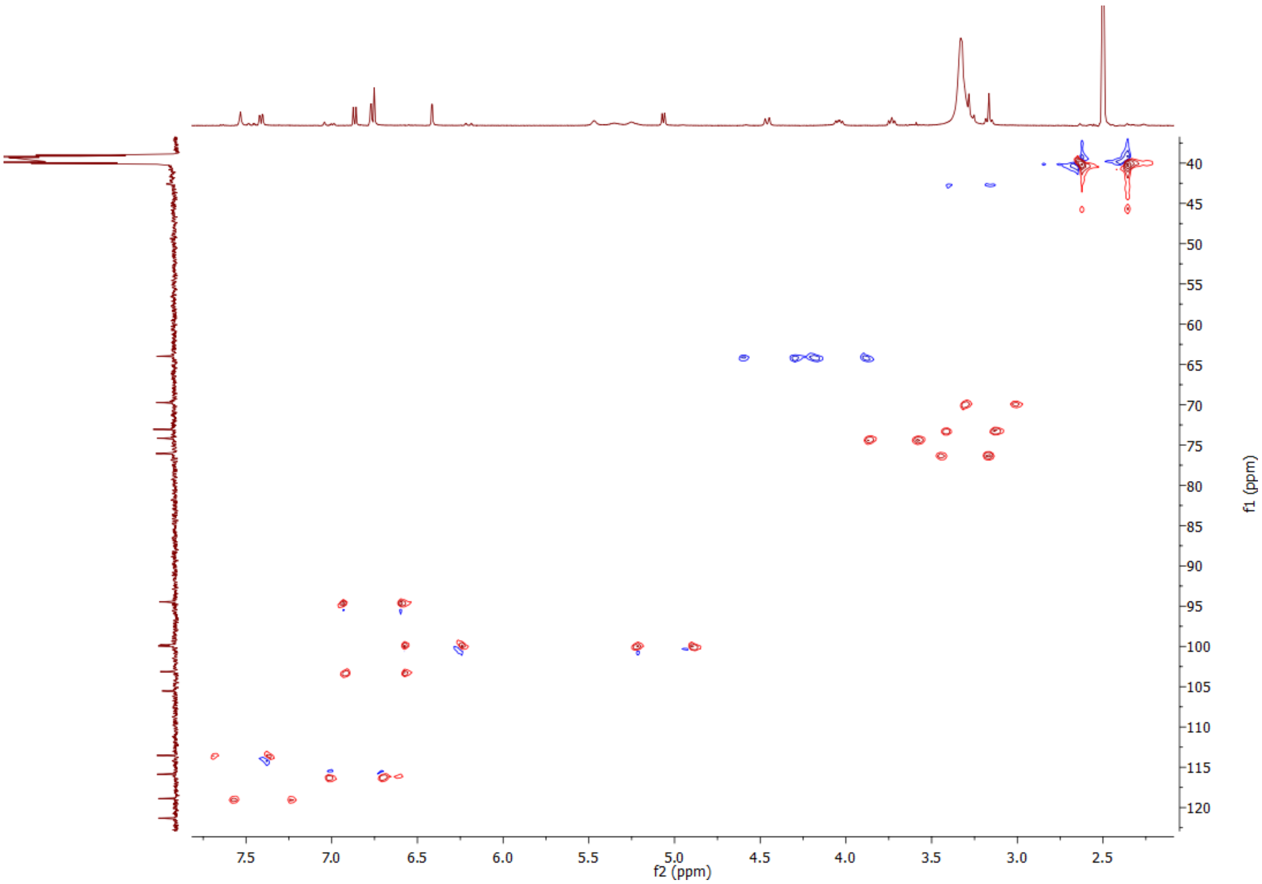

Figure S28. ^{1}H-^{13}C HSQC (DMSO-d_6, 500/125 MHz) spectrum (not decoupled) of compound **2**.

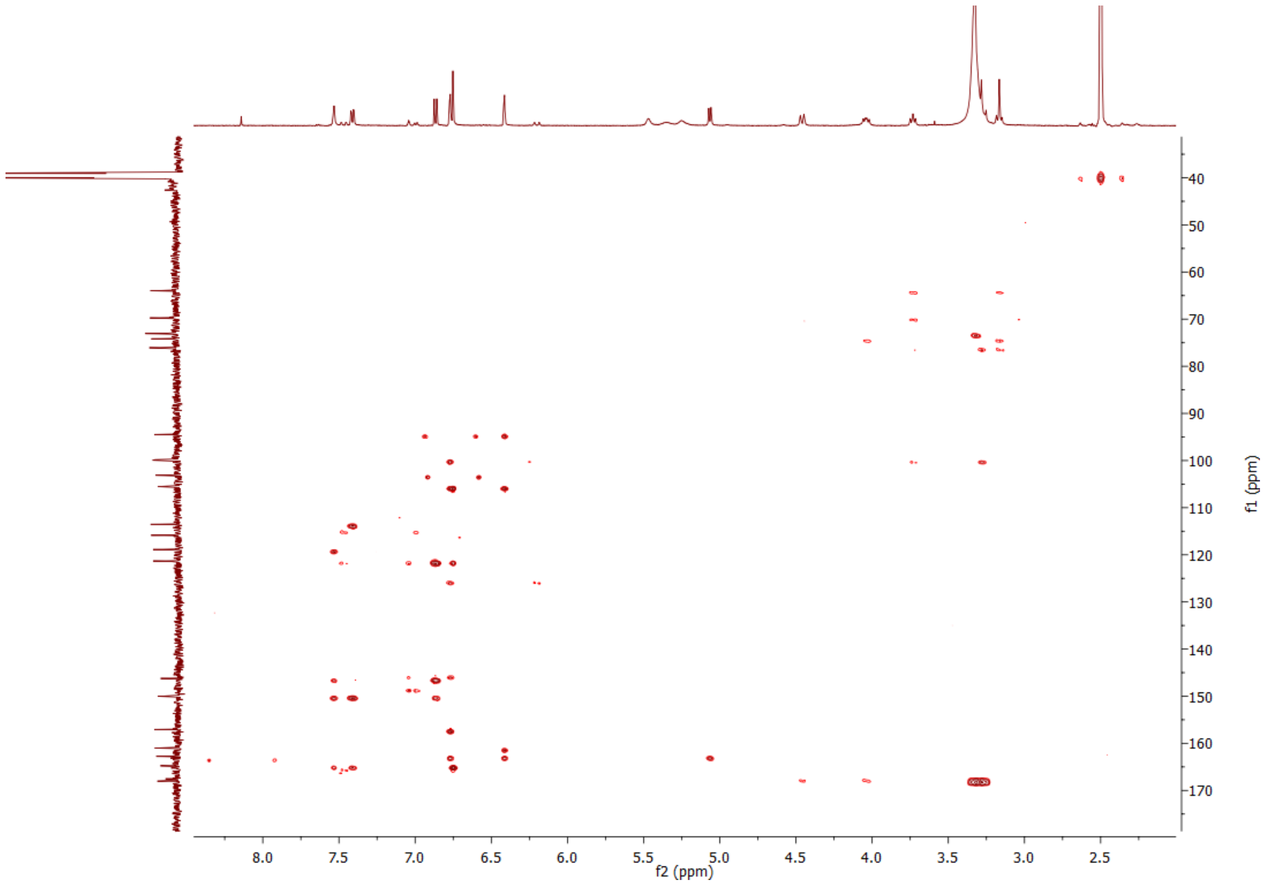

Figure S29. ^{1}H-^{13}C HMBC (DMSO-d_6, 500/125 MHz) spectrum of compound **2**.

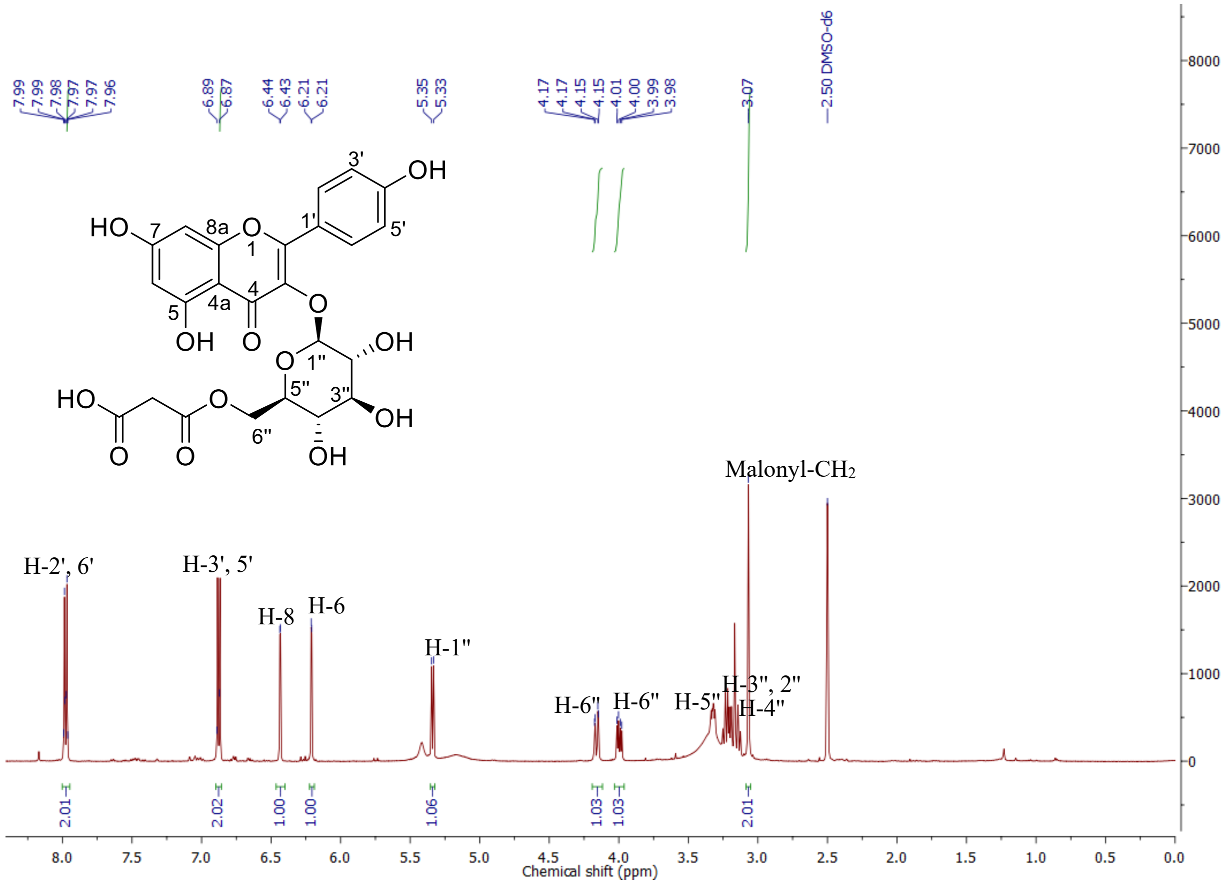

Figure S30. ^{1}H NMR (DMSO-d_6, 500 MHz) spectrum of compound **3**.

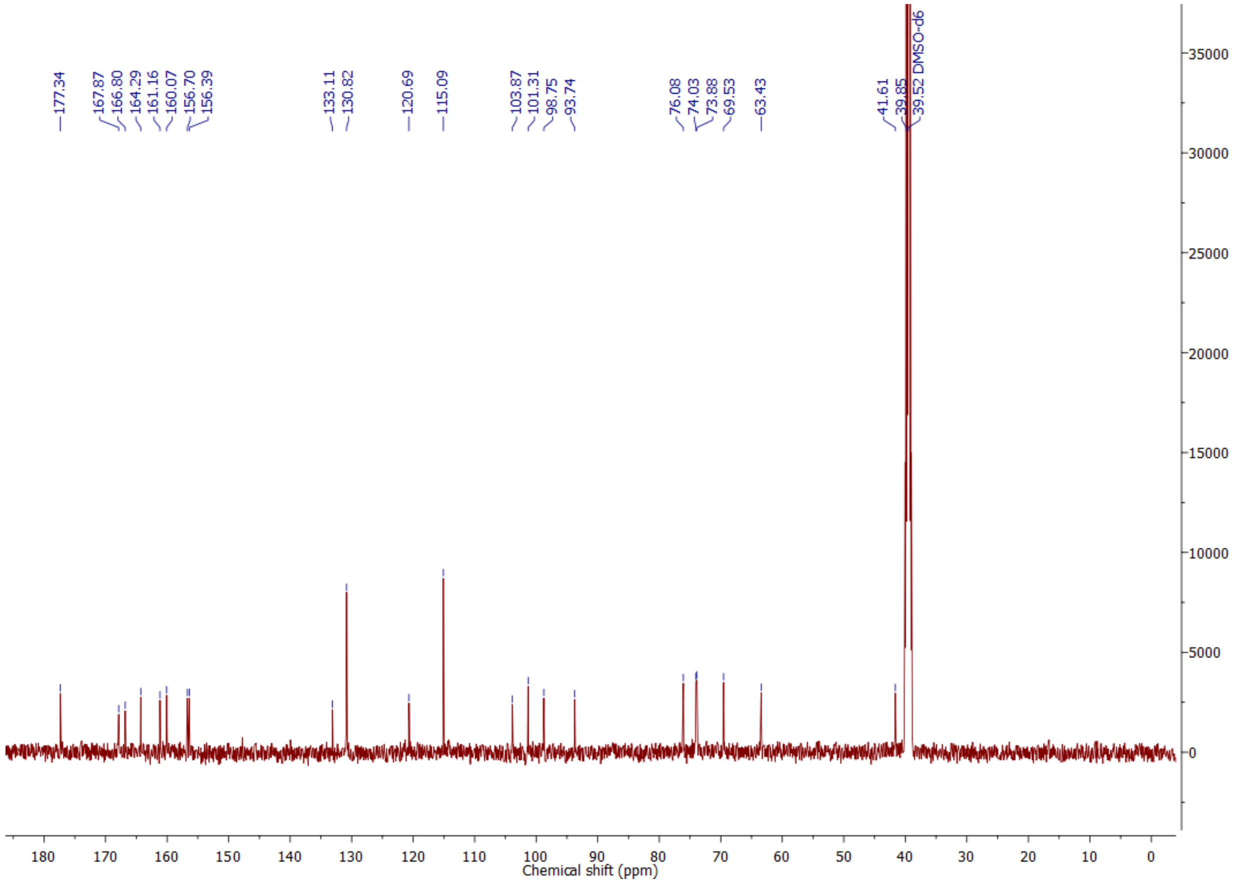

Figure S31. ^{13}C NMR (DMSO-d_6, 125 MHz) spectrum of compound **3**.

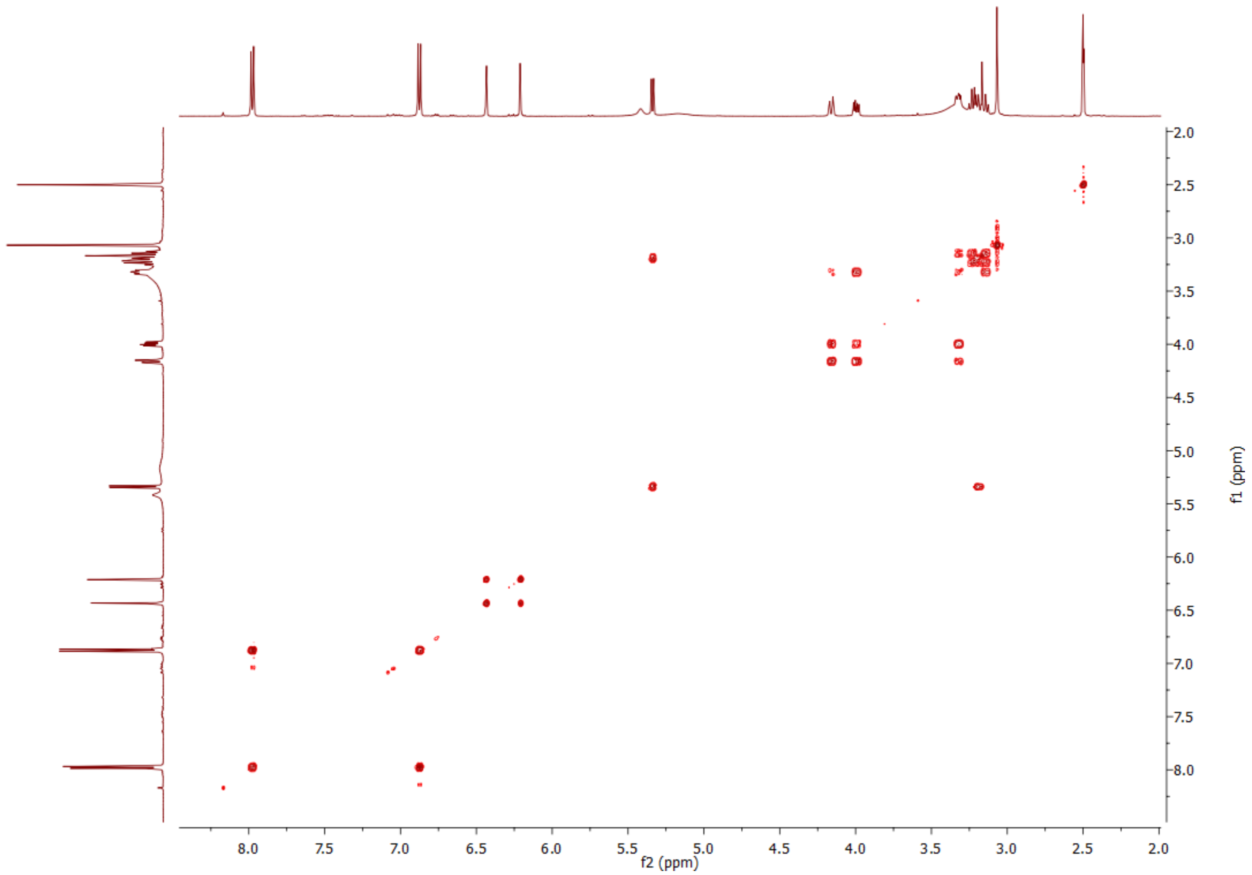

Figure S32. ^{1}H-^{1}H COSY (DMSO-d_6, 500 MHz) spectrum of compound **3**.

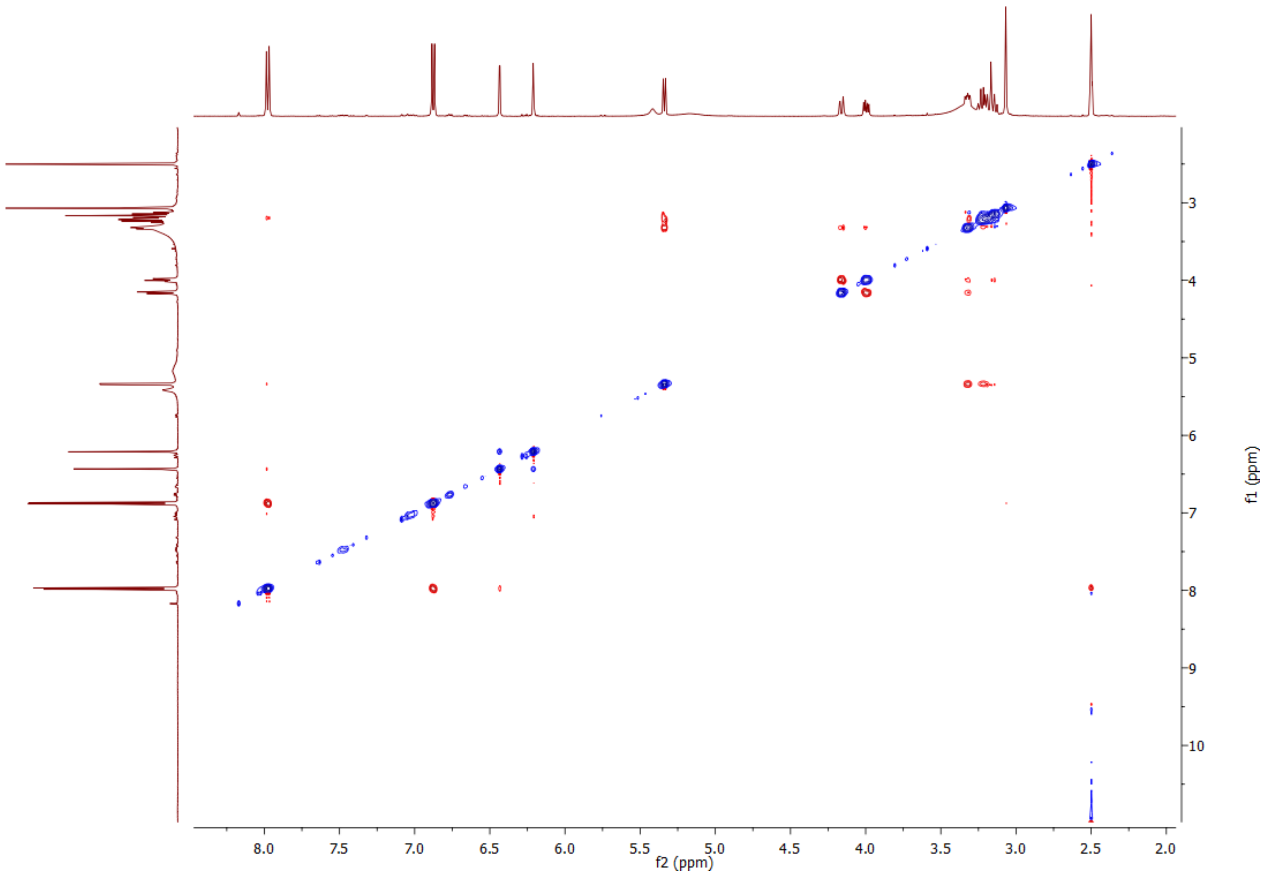

Figure S33. ^{1}H-^{1}H ROESY (DMSO-d_6, 500 MHz) spectrum of compound **3**.

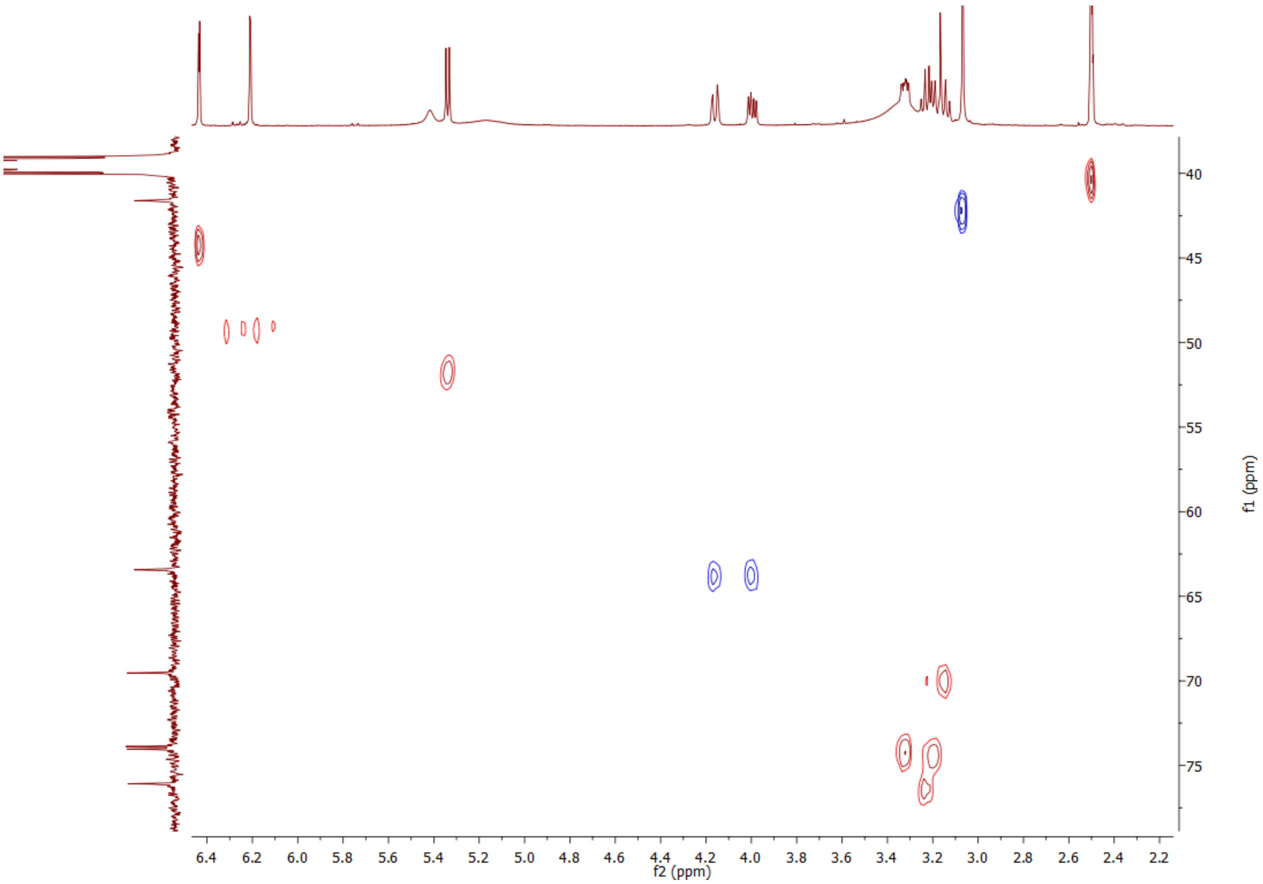

Figure S34. ^{1}H-^{13}C HSQC (DMSO-d_6, 500/125 MHz) spectrum of compound **3** for aliphatic region.

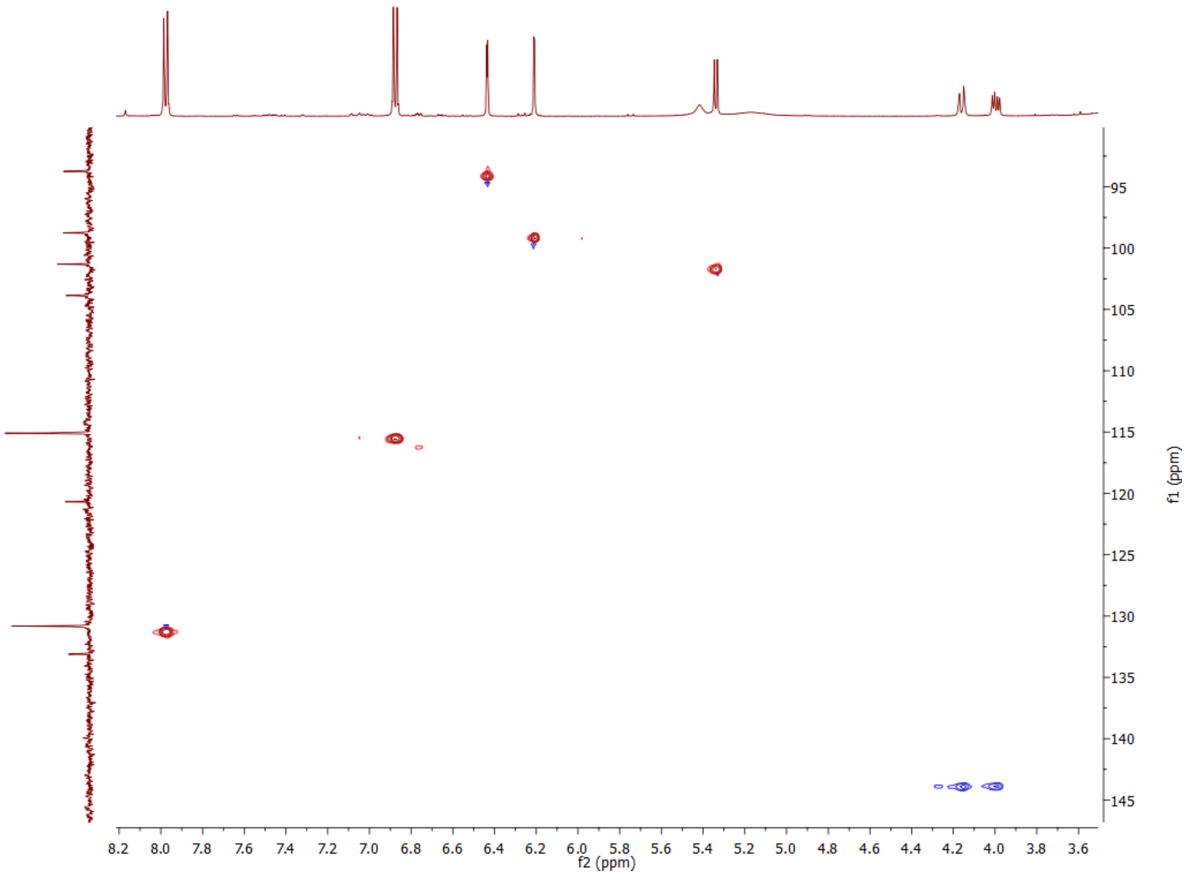

Figure S35. ^{1}H-^{13}C HSQC (DMSO-d_6, 500/125 MHz) spectrum of compound **3** for aromatic region.

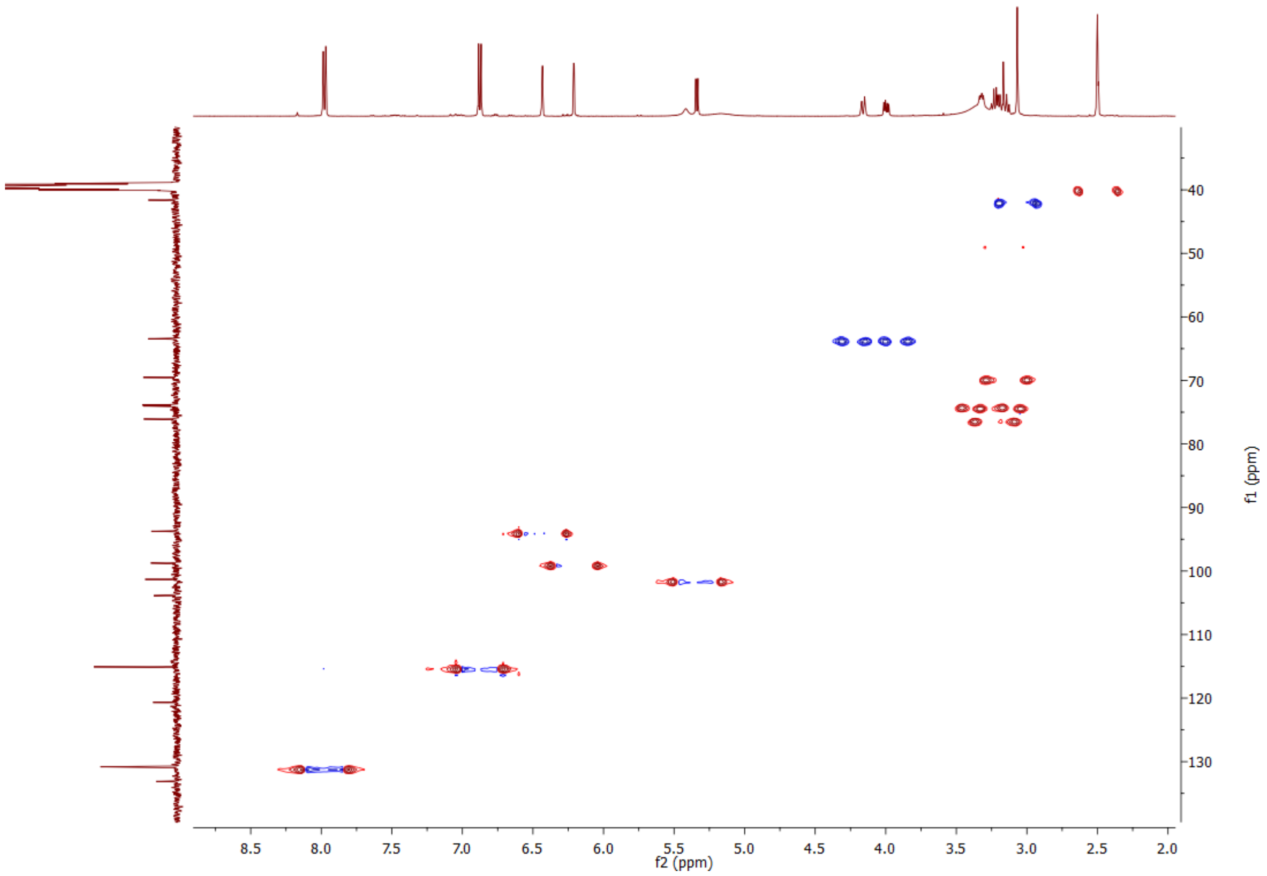

Figure S36. ^{1}H-^{13}C HSQC (DMSO-d_6, 500/125 MHz) spectrum (not decoupled) of compound **3**.

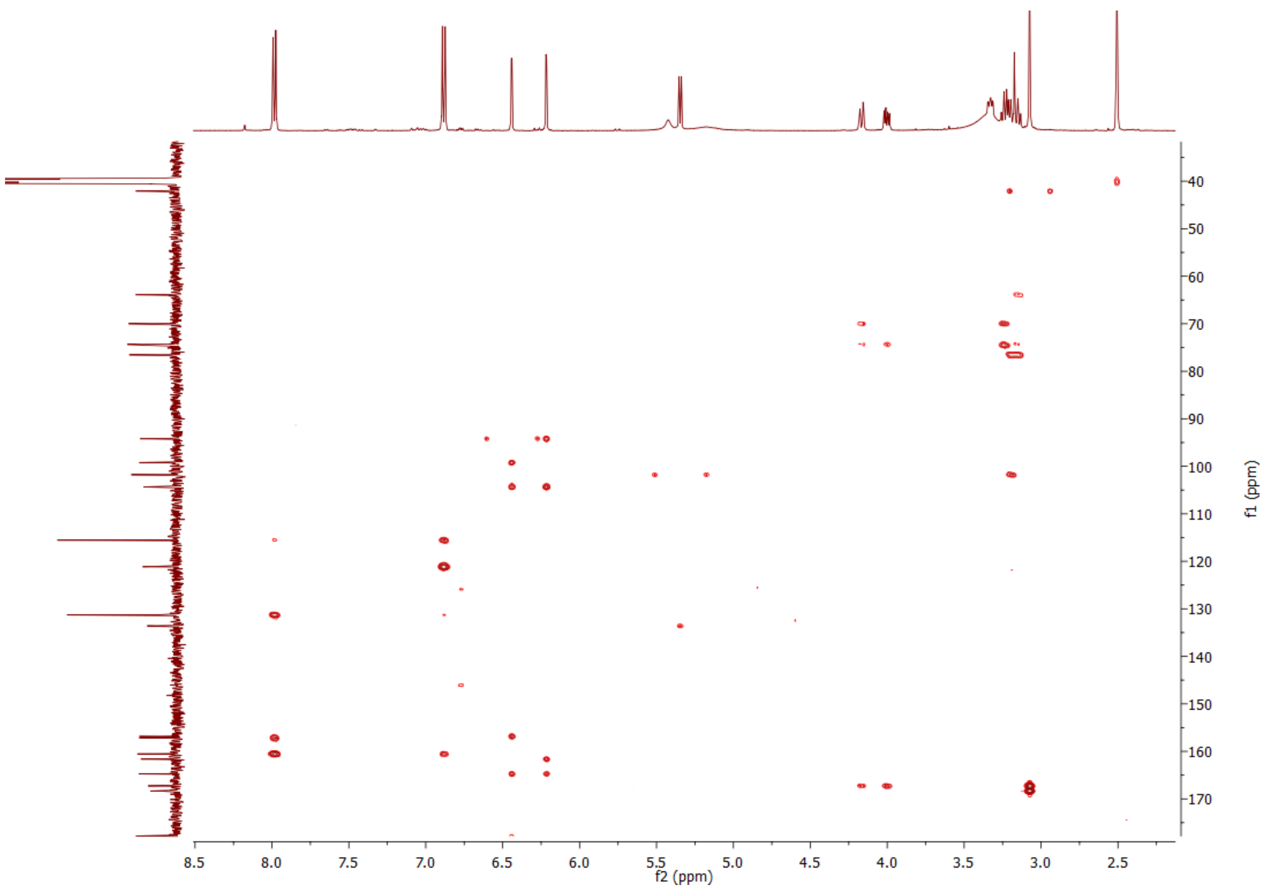

Figure S37. ^{1}H-^{13}C HMBC (DMSO-d_6, 500/125 MHz) spectrum of compound **3**.

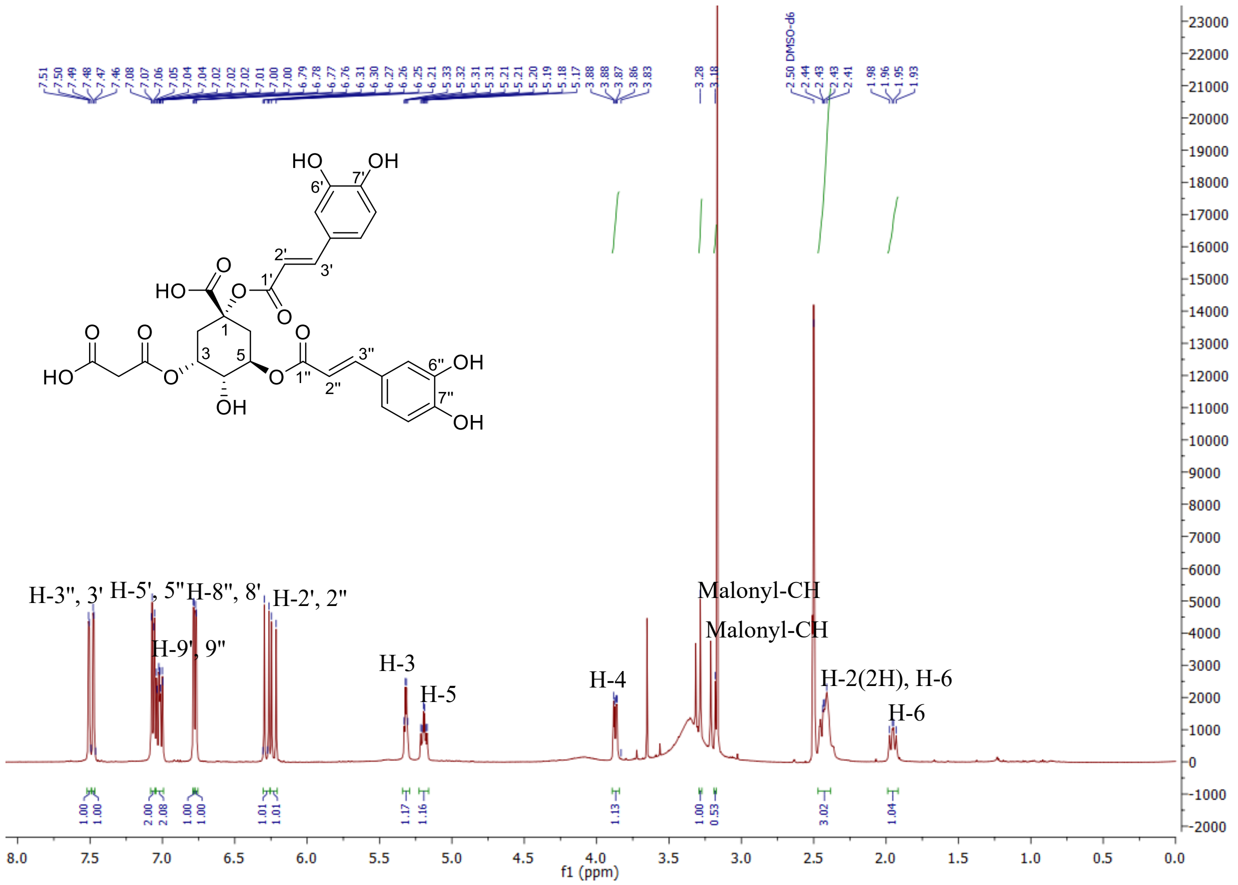

Figure S38. ^{1}H NMR (DMSO-d_6, 500 MHz) spectrum of compound **4**.

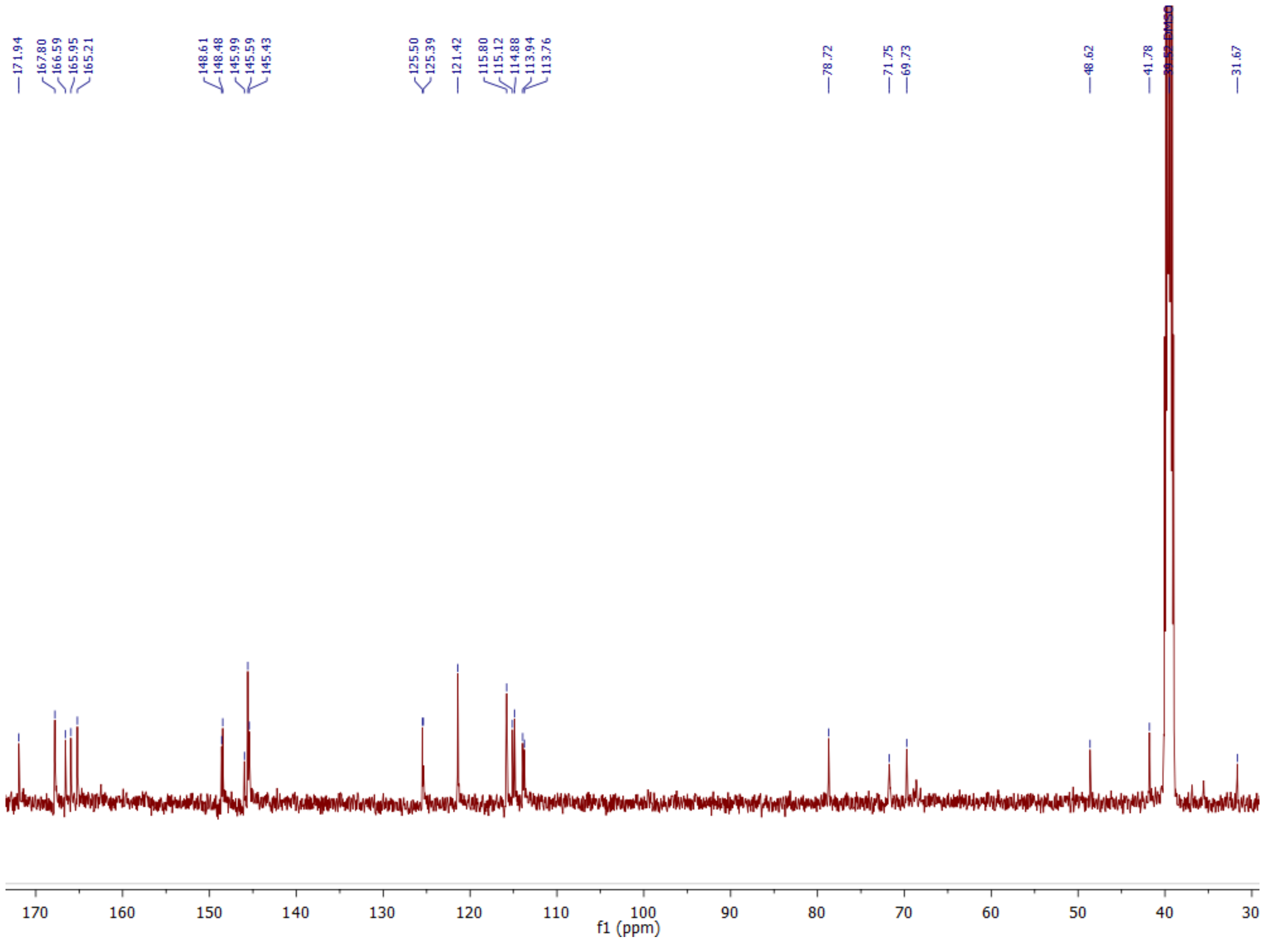

Figure S39. ^{13}C NMR (DMSO-d_6, 125 MHz) spectrum of compound **4**.

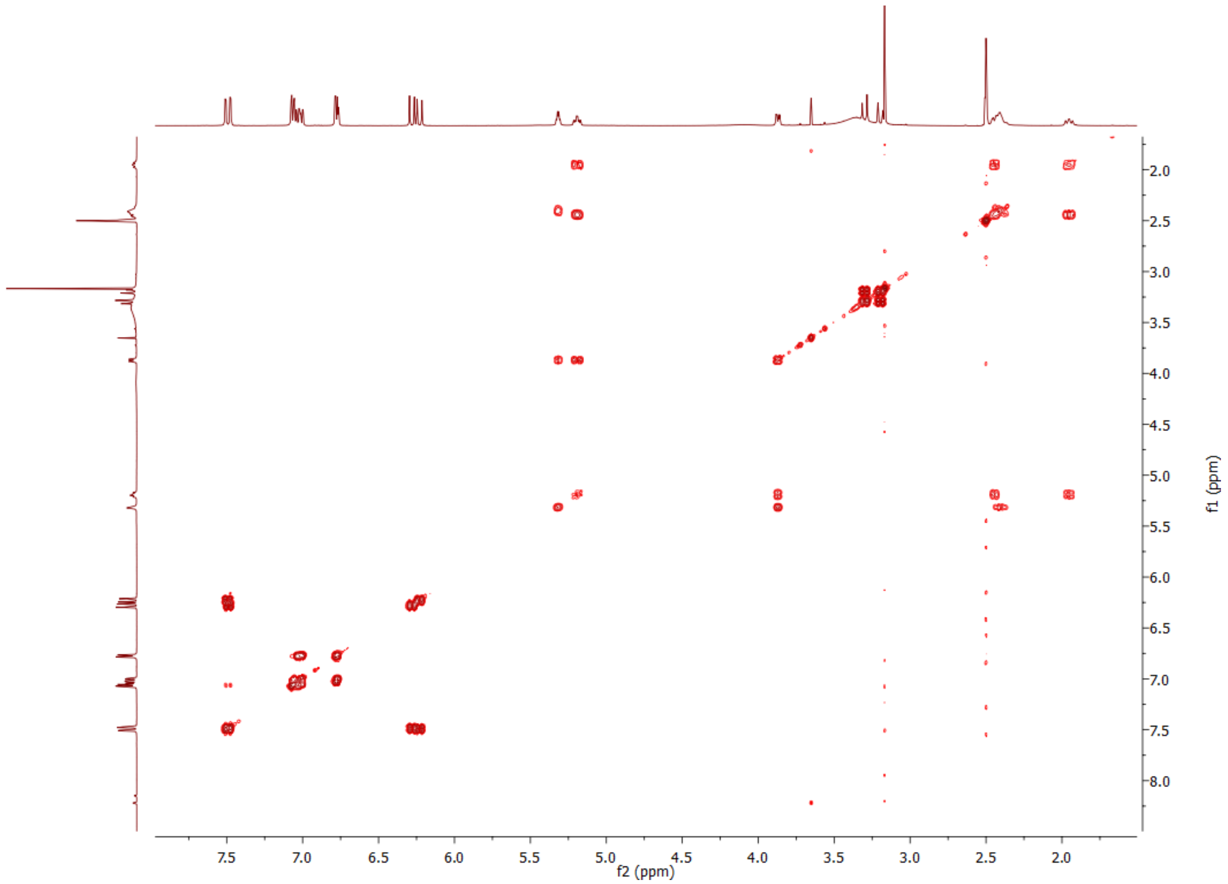

Figure S40. ^{1}H-^{1}H COSY (DMSO-d_6, 500 MHz) spectrum of compound **4**.

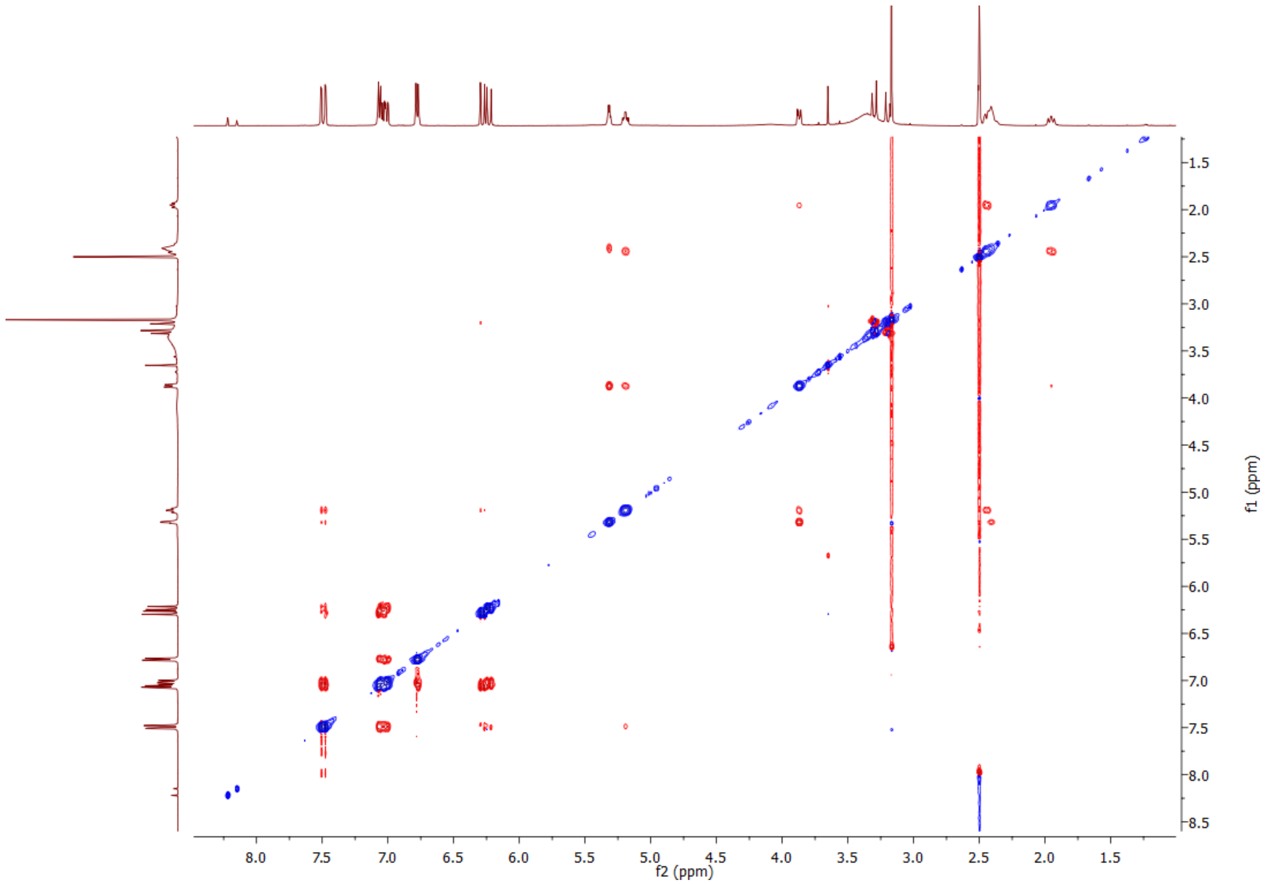

Figure S41. ^{1}H-^{1}H ROESY (DMSO-d_6, 500 MHz) spectrum of compound **4**.

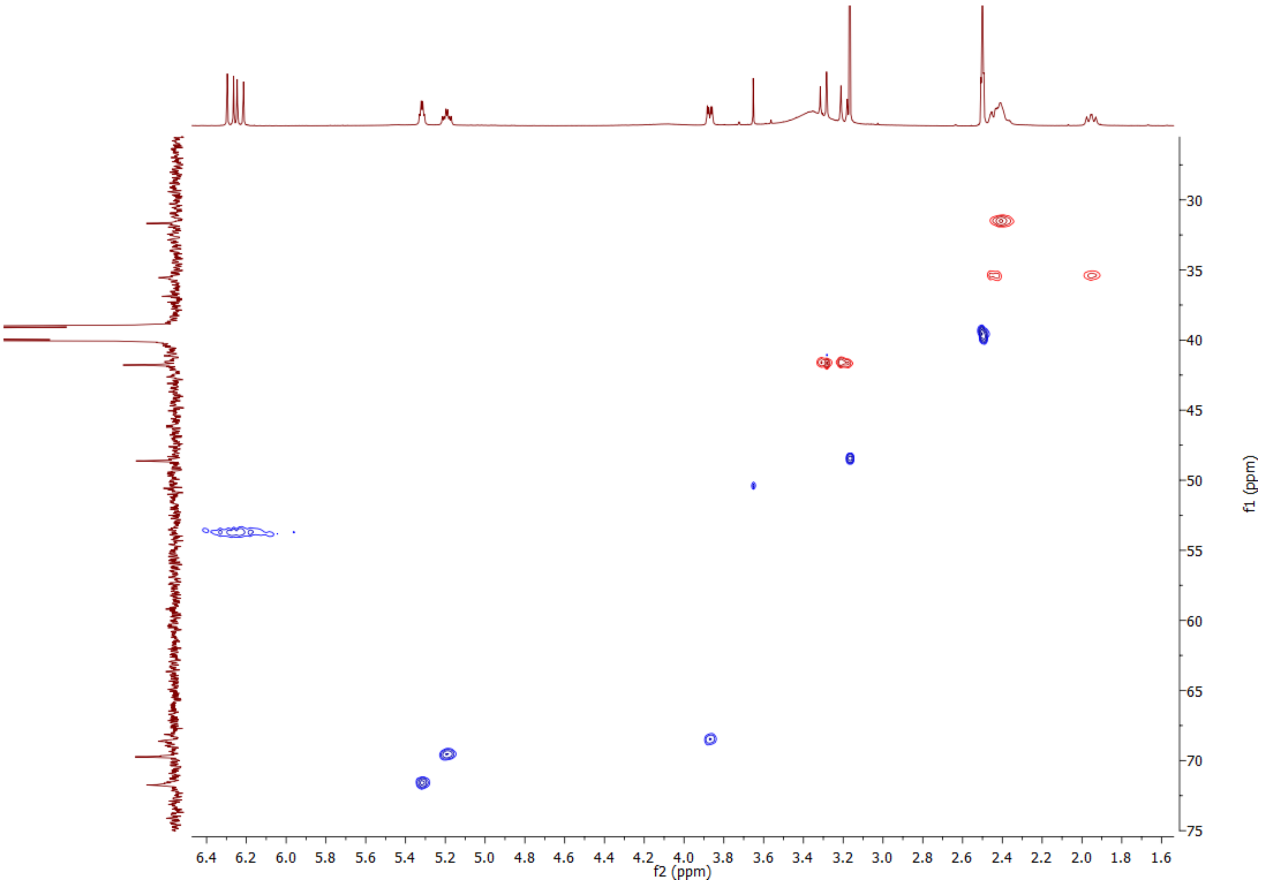

Figure S42. ^{1}H-^{13}C HSQC (DMSO-d_6, 500/125 MHz) spectrum of compound **4** for aliphatic region.

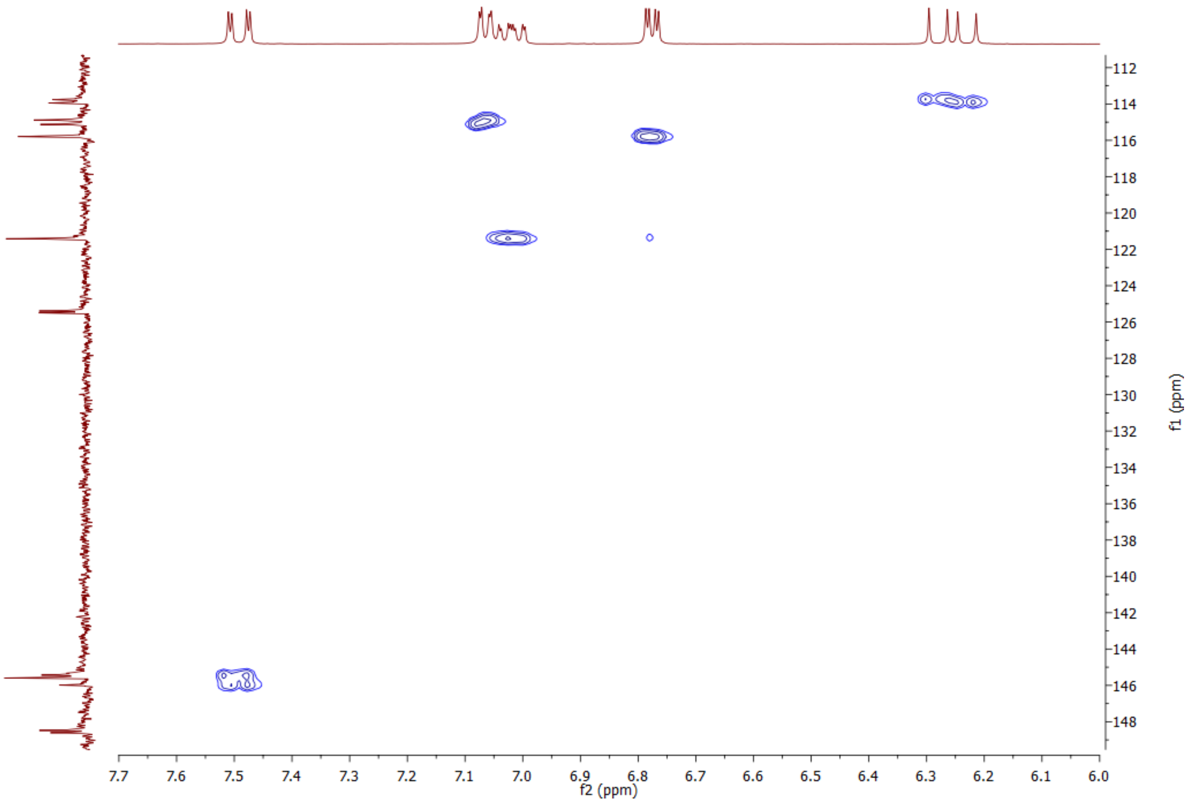

Figure S43. ^{1}H-^{13}C HSQC (DMSO-d_6, 500/125 MHz) spectrum of compound **4** for aromatic region.

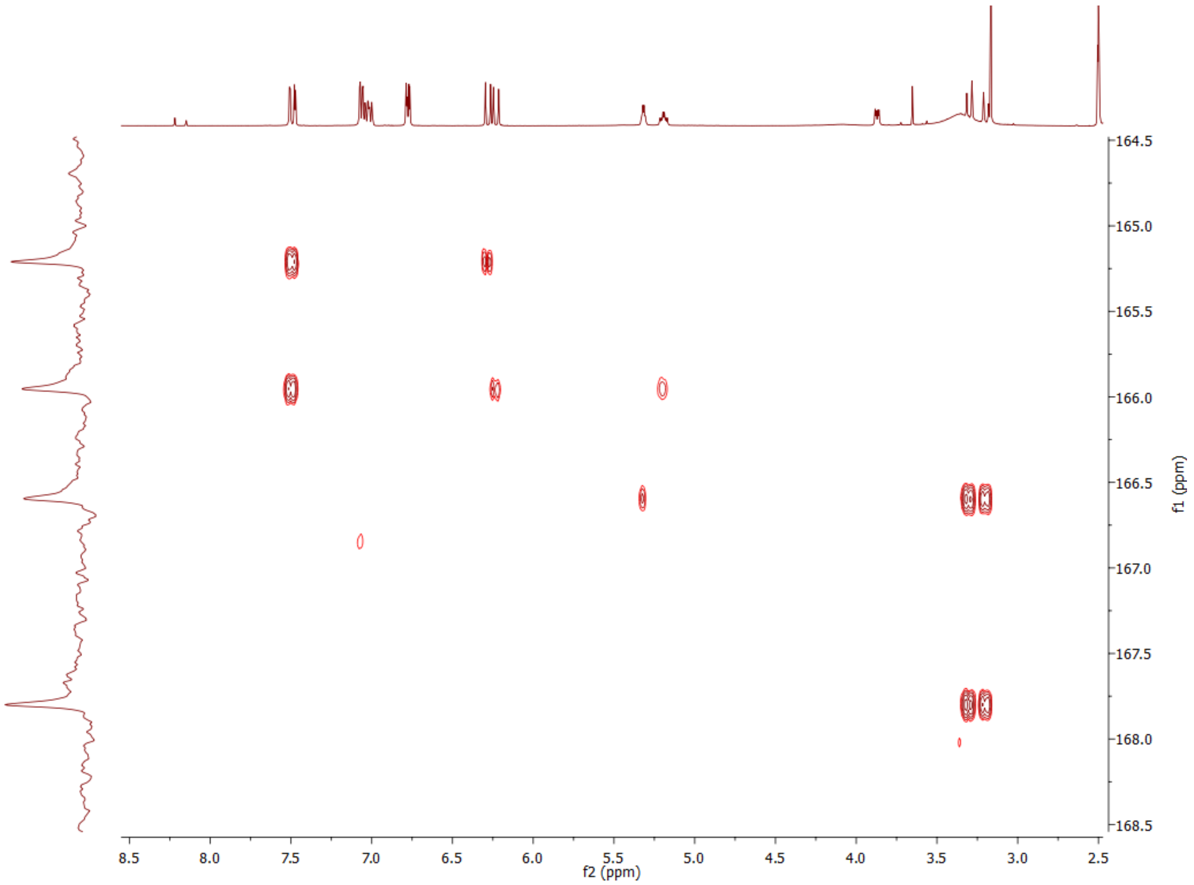

Figure S44. Band-selective ^{1}H-^{13}C HMBC (DMSO-d_6, 500/125 MHz) spectrum of compound **4**.

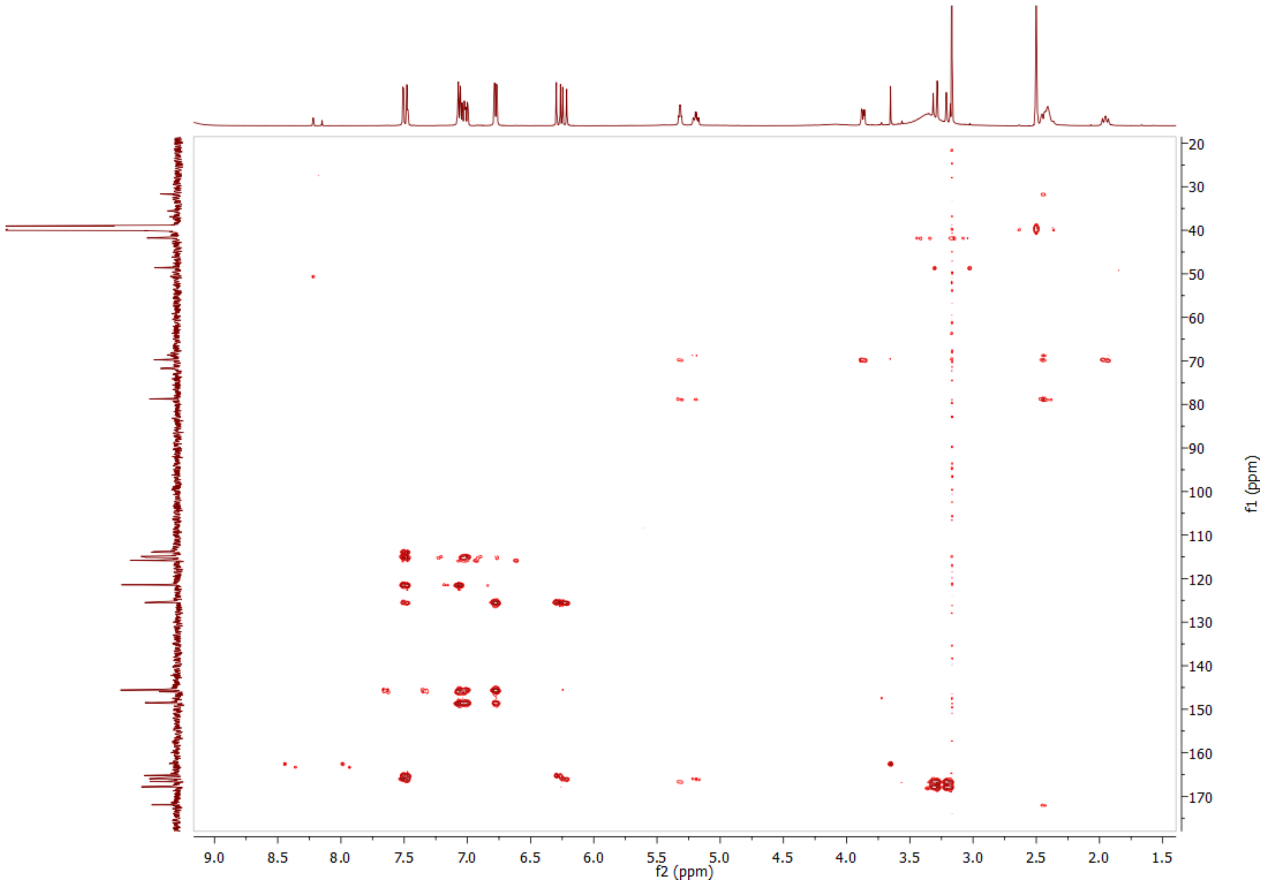

Figure S45. Full ^{1}H-^{13}C HMBC (DMSO-d_6, 500/125 MHz) spectrum of compound **4**.

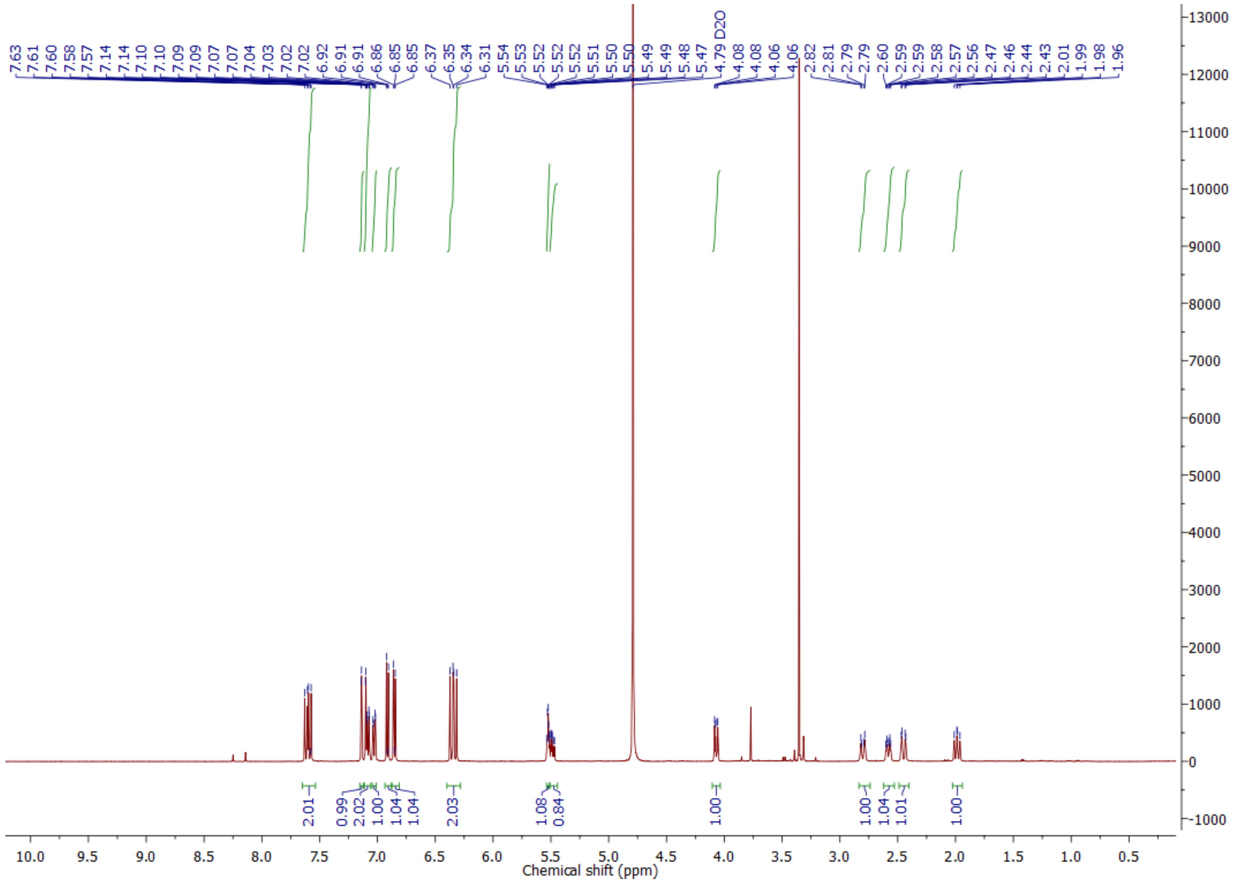

Figure S46. ^1H NMR (D_2O, 500 MHz) spectrum of compound **4**.

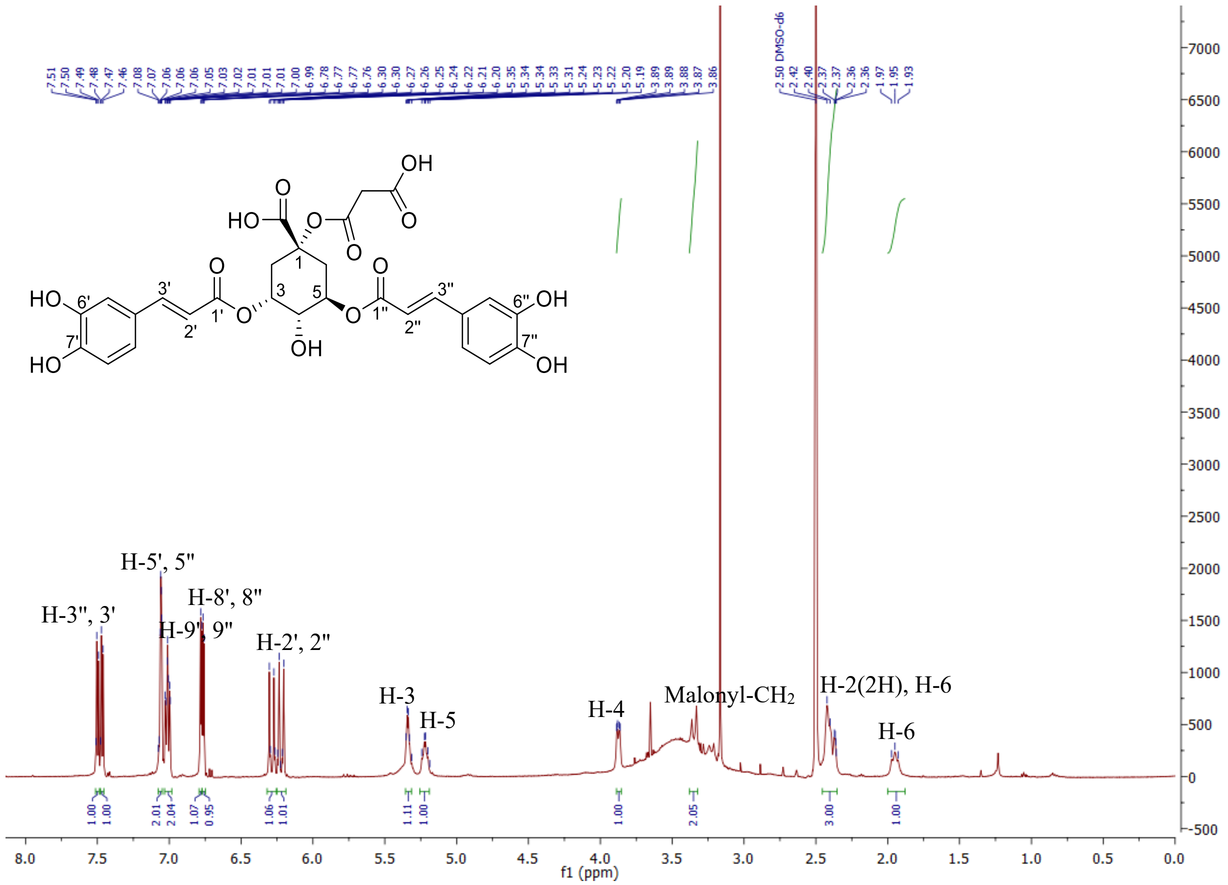

Figure S47. ^{1}H NMR (DMSO-d_6, 500 MHz) spectrum of compound **5**.

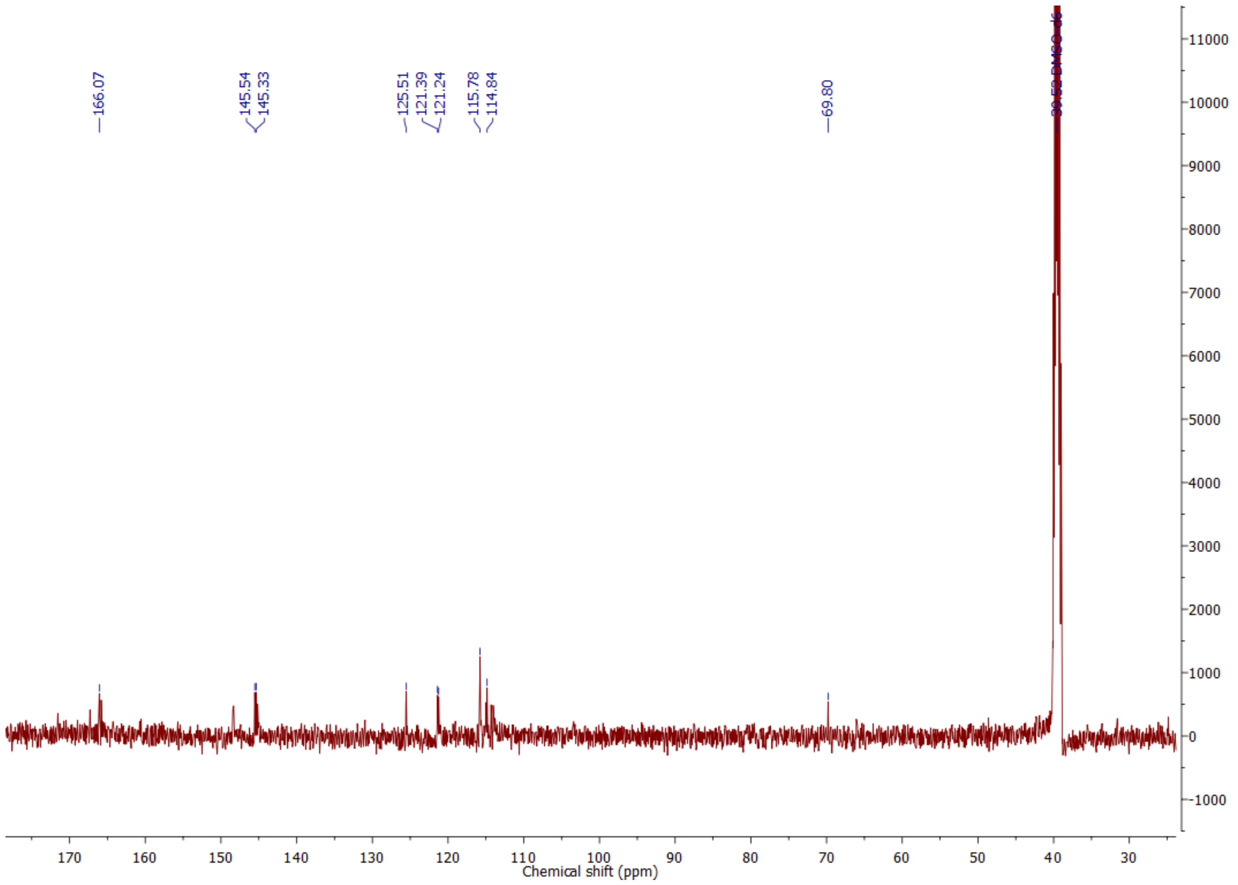

Figure S48. ^{13}C NMR (DMSO-d_6, 125 MHz) spectrum of compound **5**.

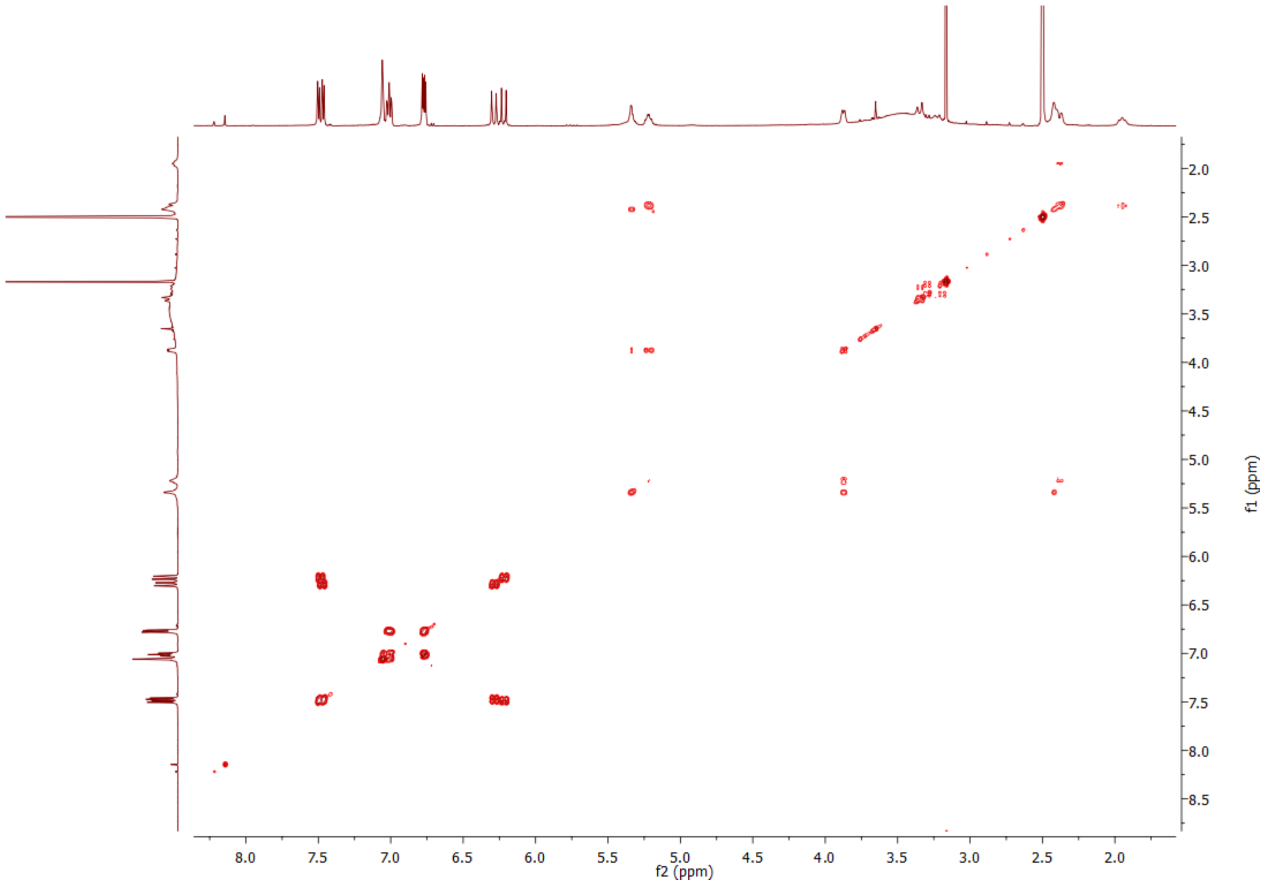

Figure S49. ^{1}H-^{1}H COSY (DMSO-d_6, 500 MHz) spectrum of compound **5**.

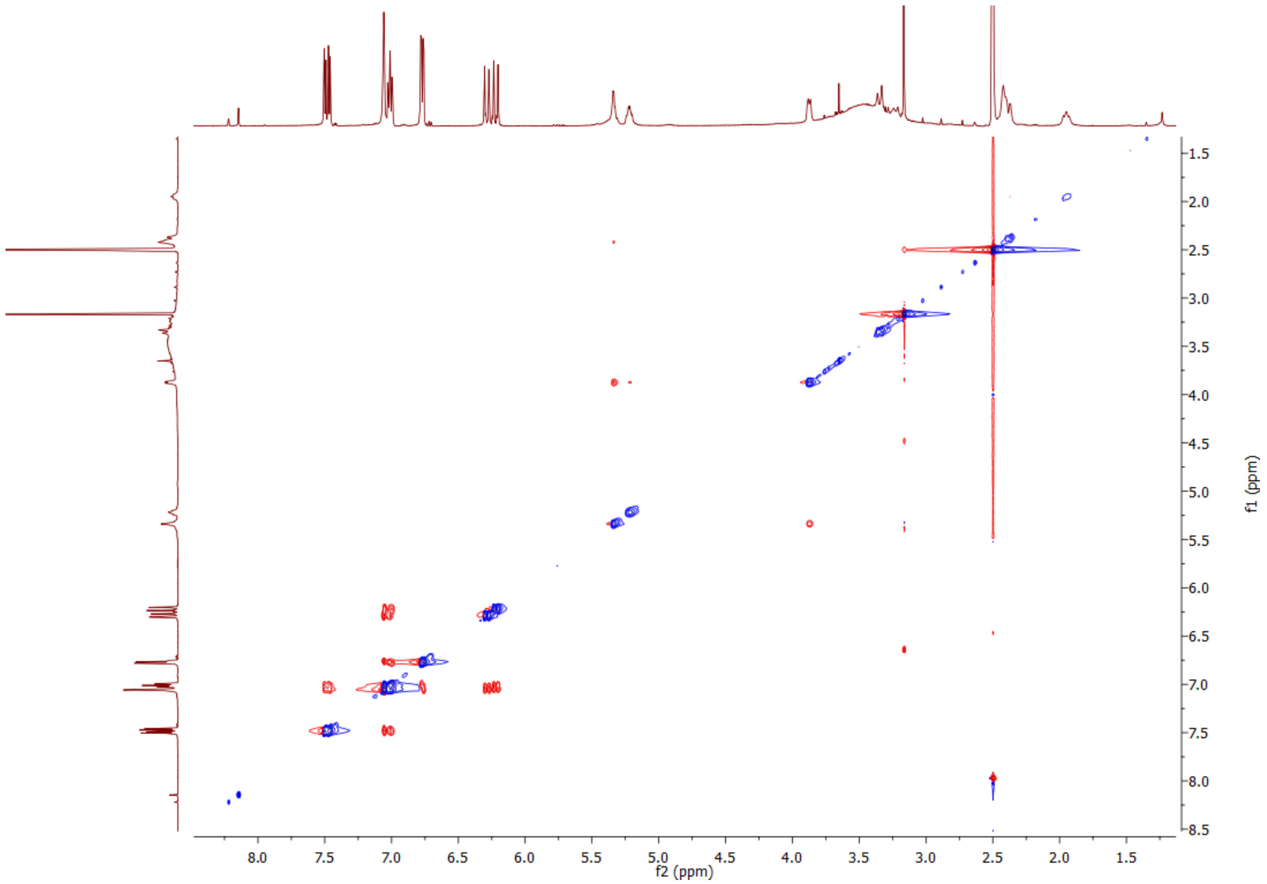

Figure S50. ^1H-^1H ROESY (DMSO-d_6, 500 MHz) spectrum of compound **5**.

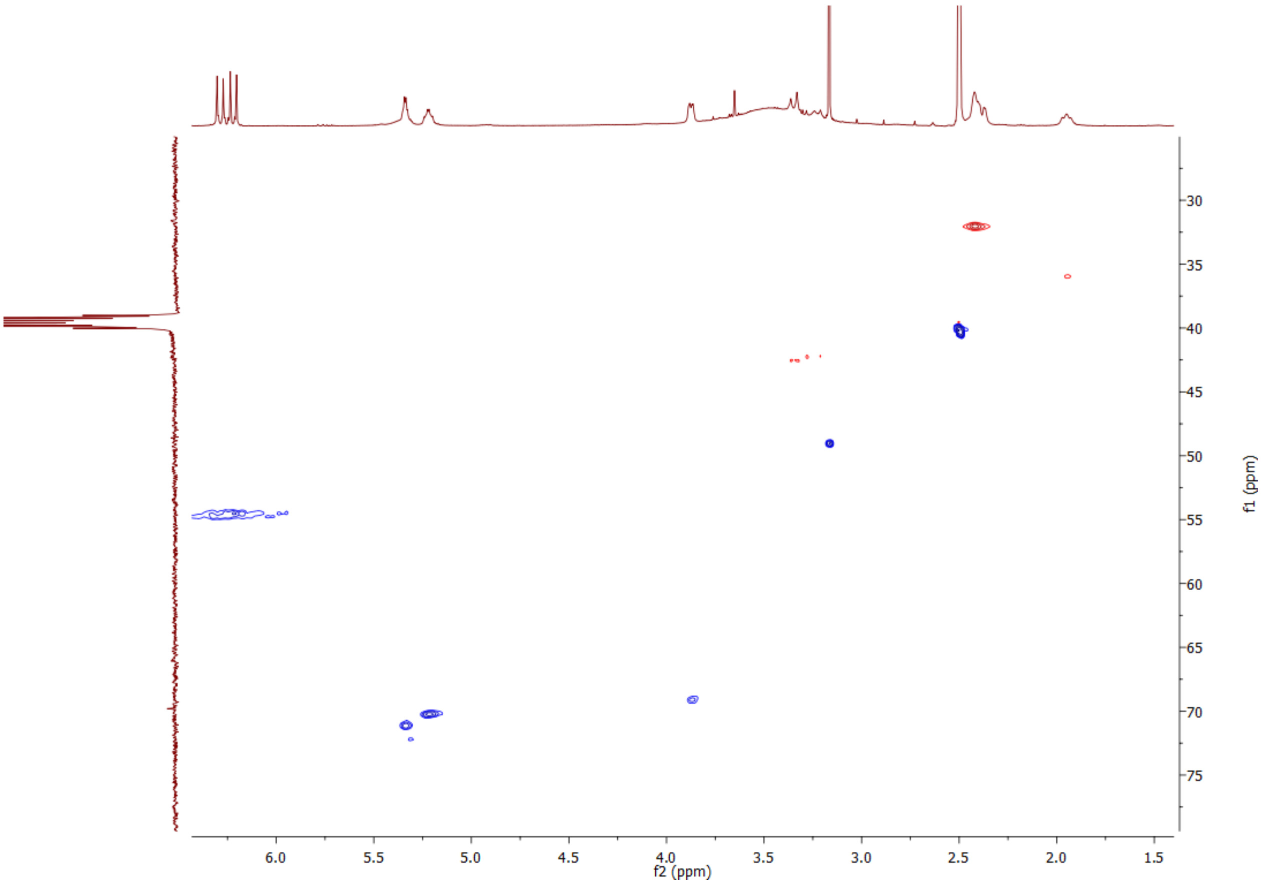

Figure S51. ^{1}H-^{13}C HSQC (DMSO-d_6, 500/125 MHz) spectrum of compound **5** for aliphatic region.

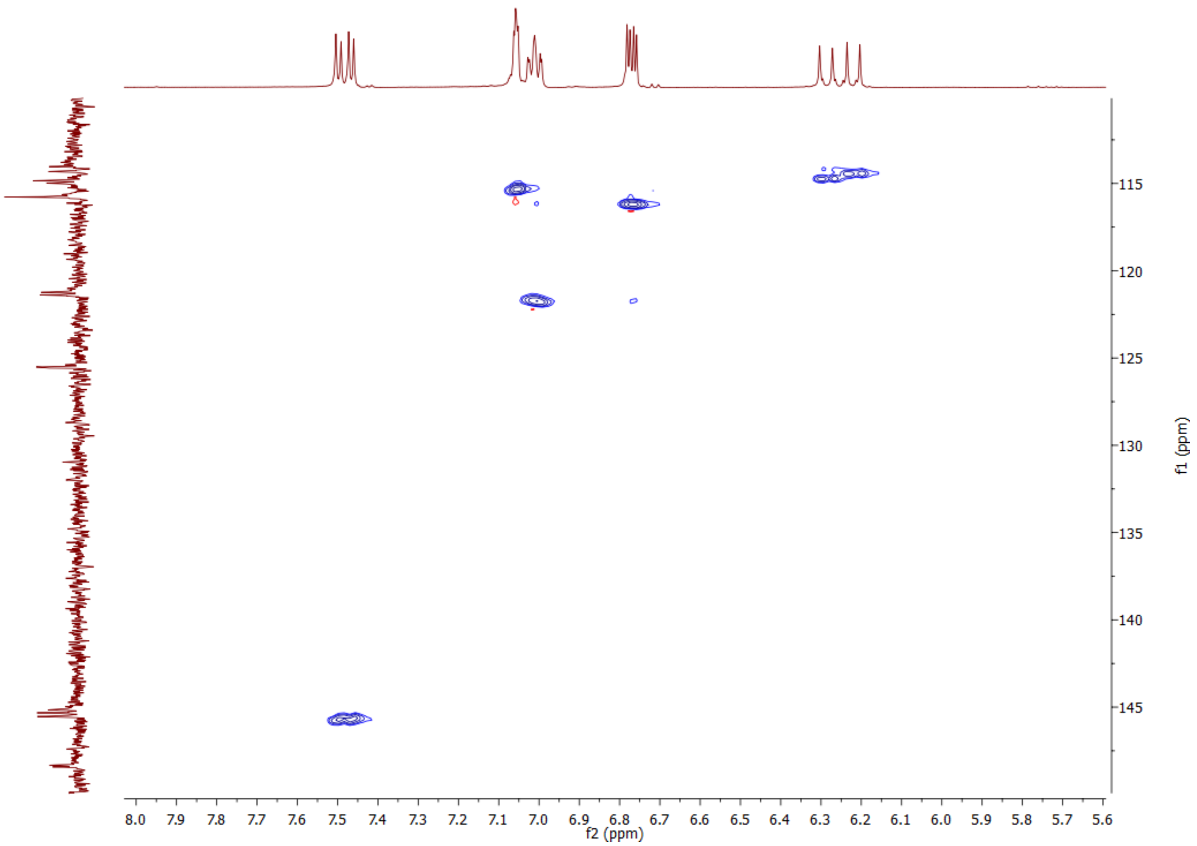

Figure S52. ^1H-^{13}C HSQC (DMSO-d_6, 500/125 MHz) spectrum of compound **5** for aromatic region.

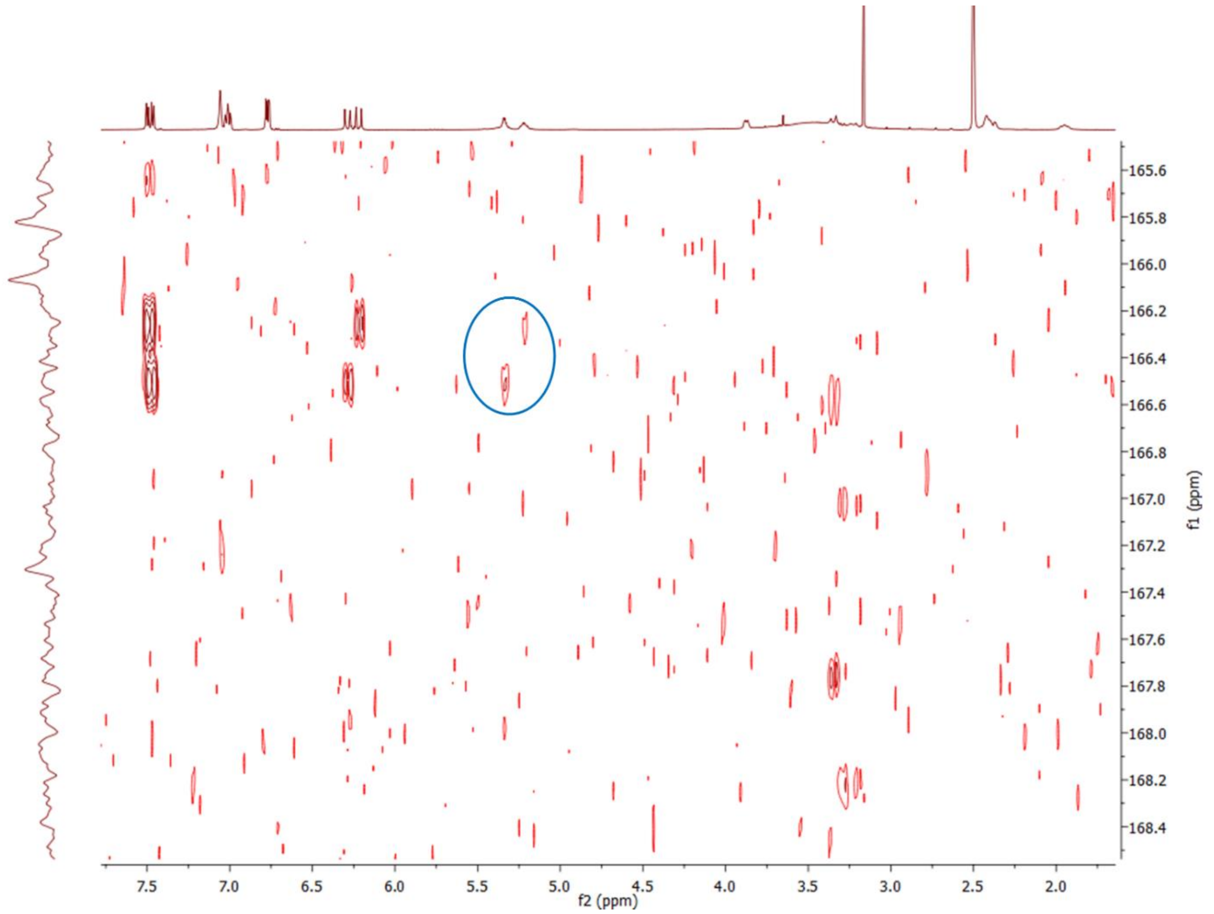

Figure S53. Band-selective ^{1}H-^{13}C HMBC (DMSO-d_6, 500/125 MHz) spectrum of compound **5**.

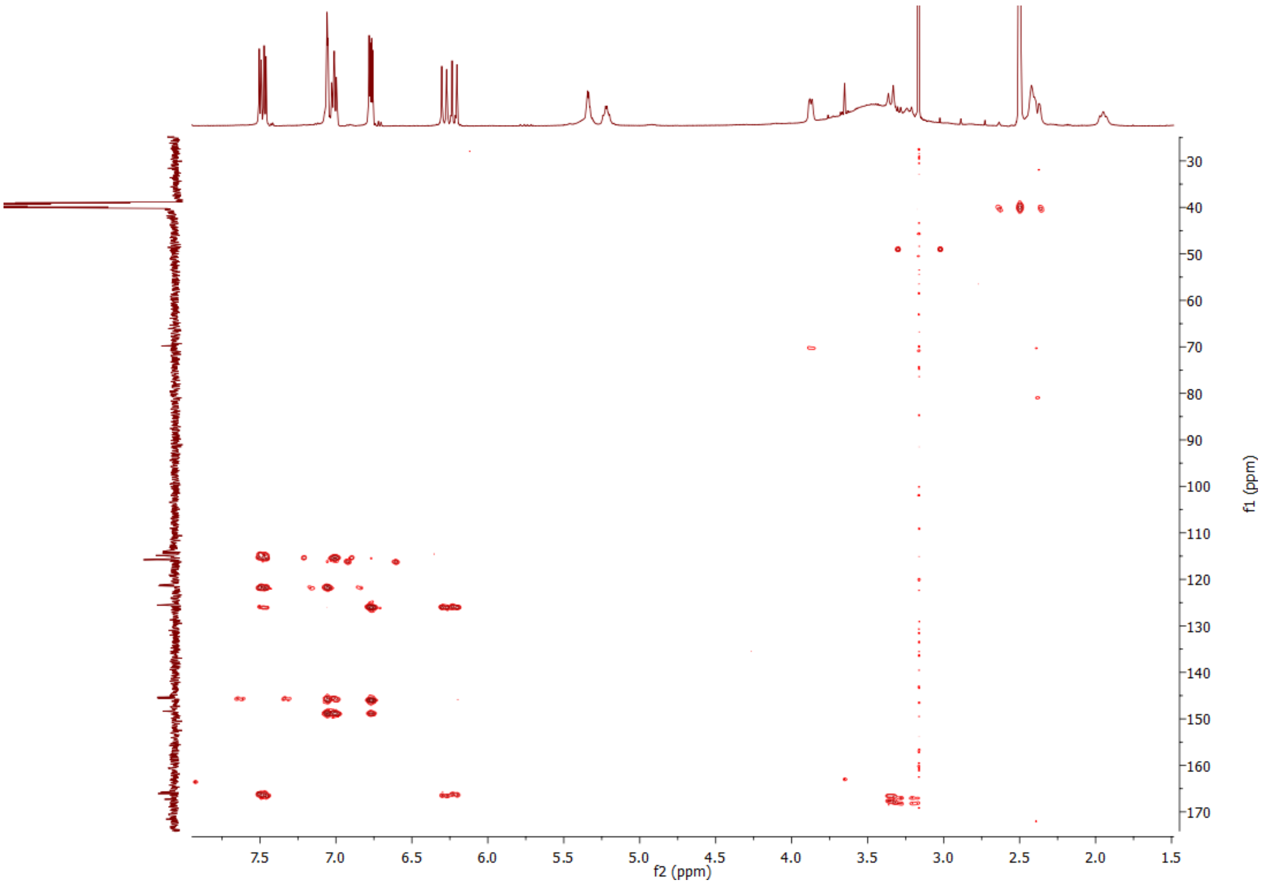

Figure S54. Full ^{1}H-^{13}C HMBC (DMSO-d_6, 500/125 MHz) spectrum of compound **5**.

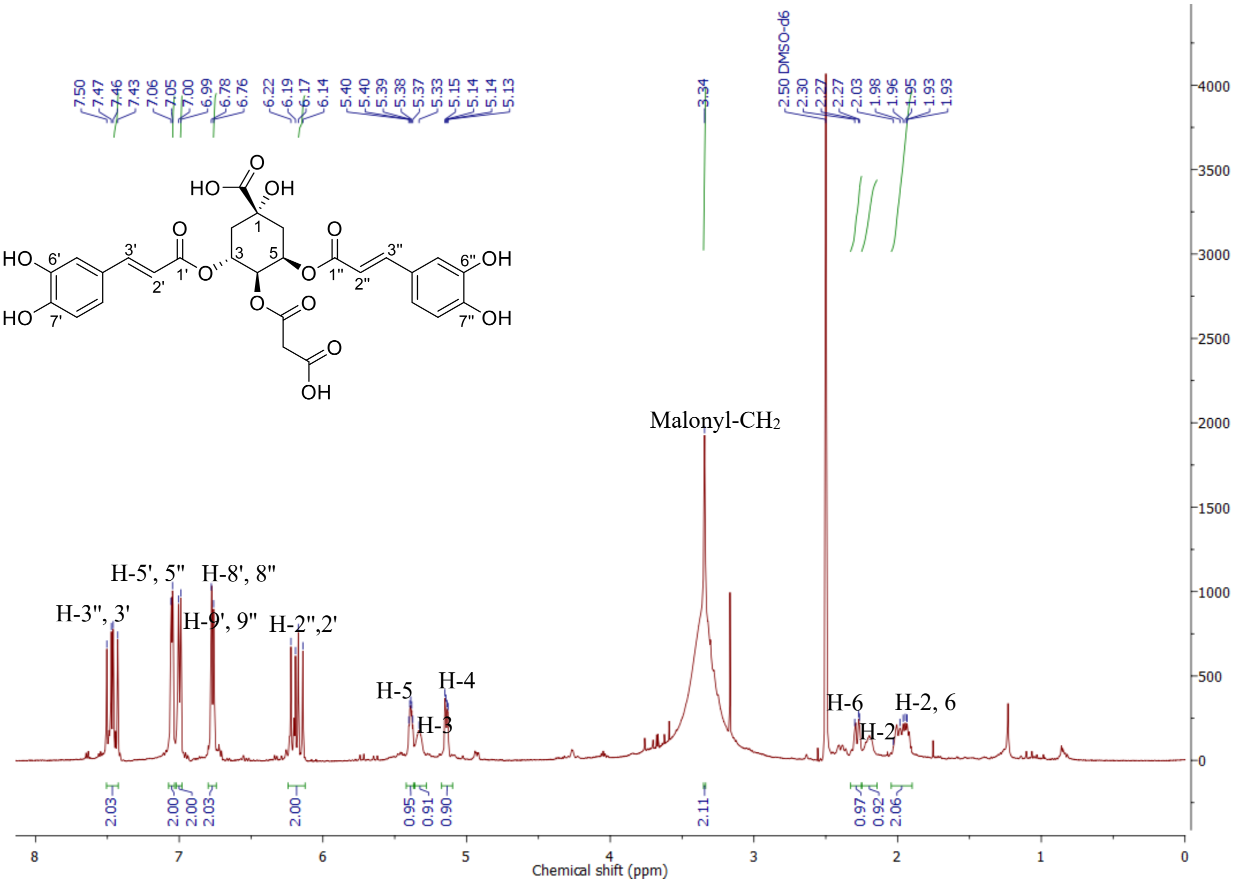

Figure S55. ^{1}H NMR (DMSO-d_6, 500 MHz) spectrum of compound **6**.

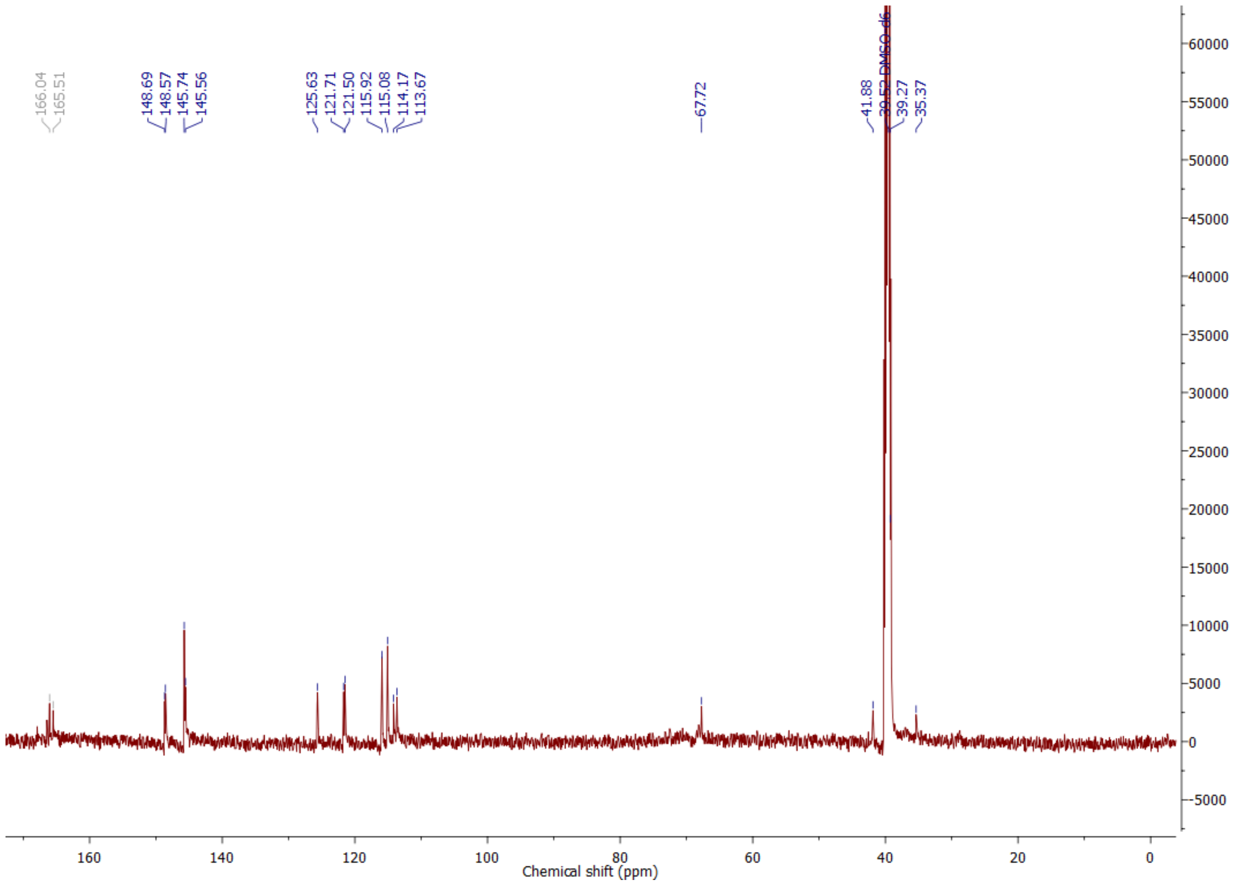

Figure S56. ^{13}C NMR (DMSO-d_6, 500 MHz) spectrum of compound **6**.

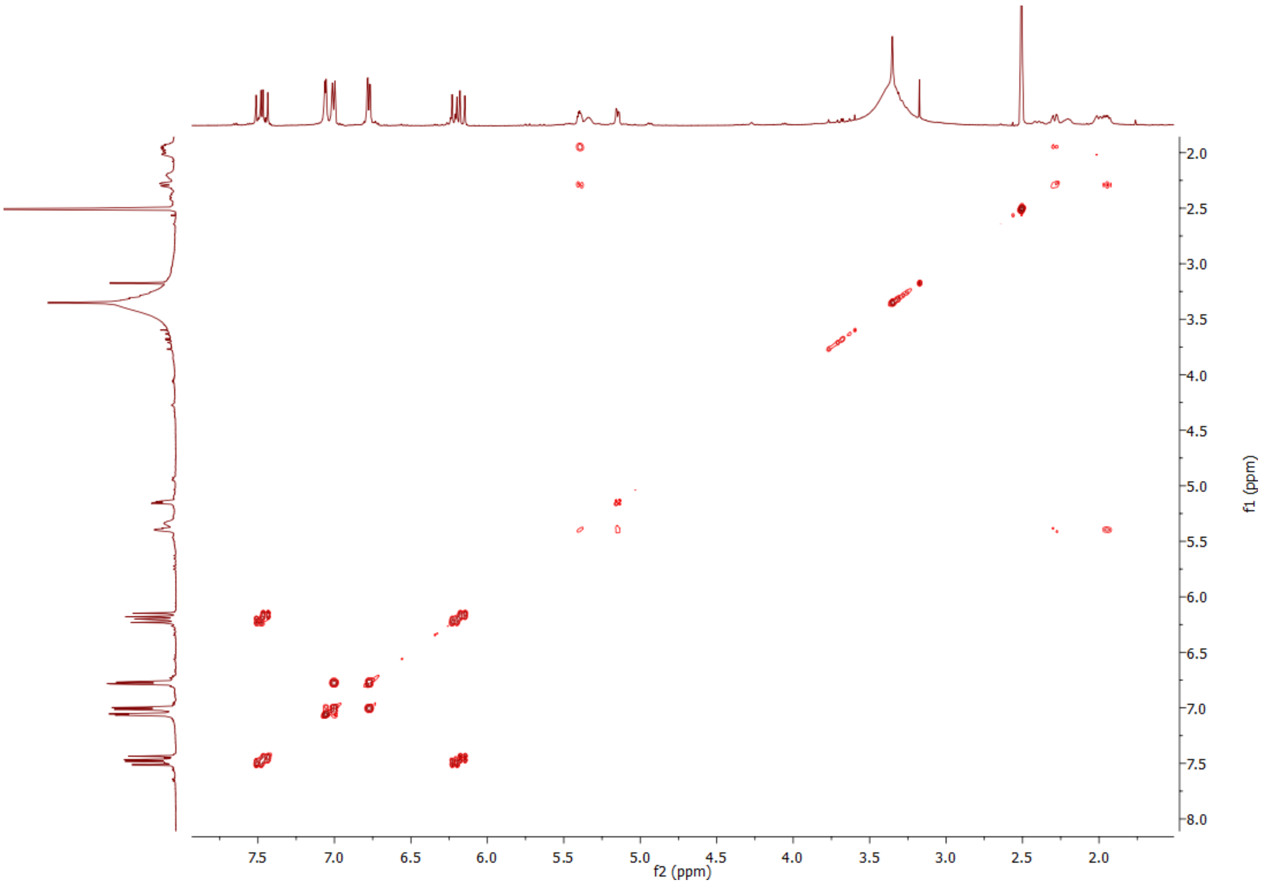

Figure S57. ^{1}H-^{1}H COSY (DMSO-d_6, 500 MHz) spectrum of compound **6**.

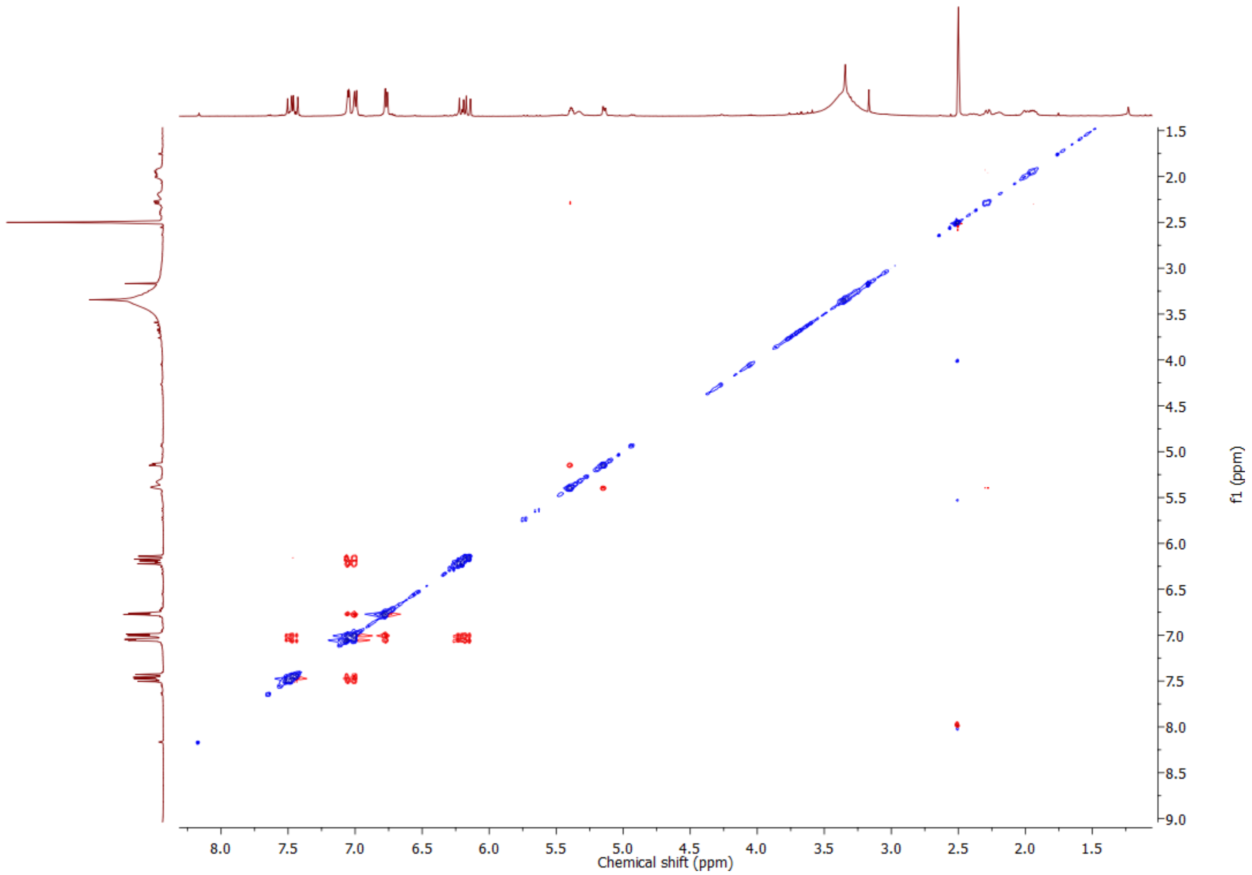

Figure S58. ^{1}H-^{1}H ROESY (DMSO-d_6, 500 MHz) spectrum of compound **6**.

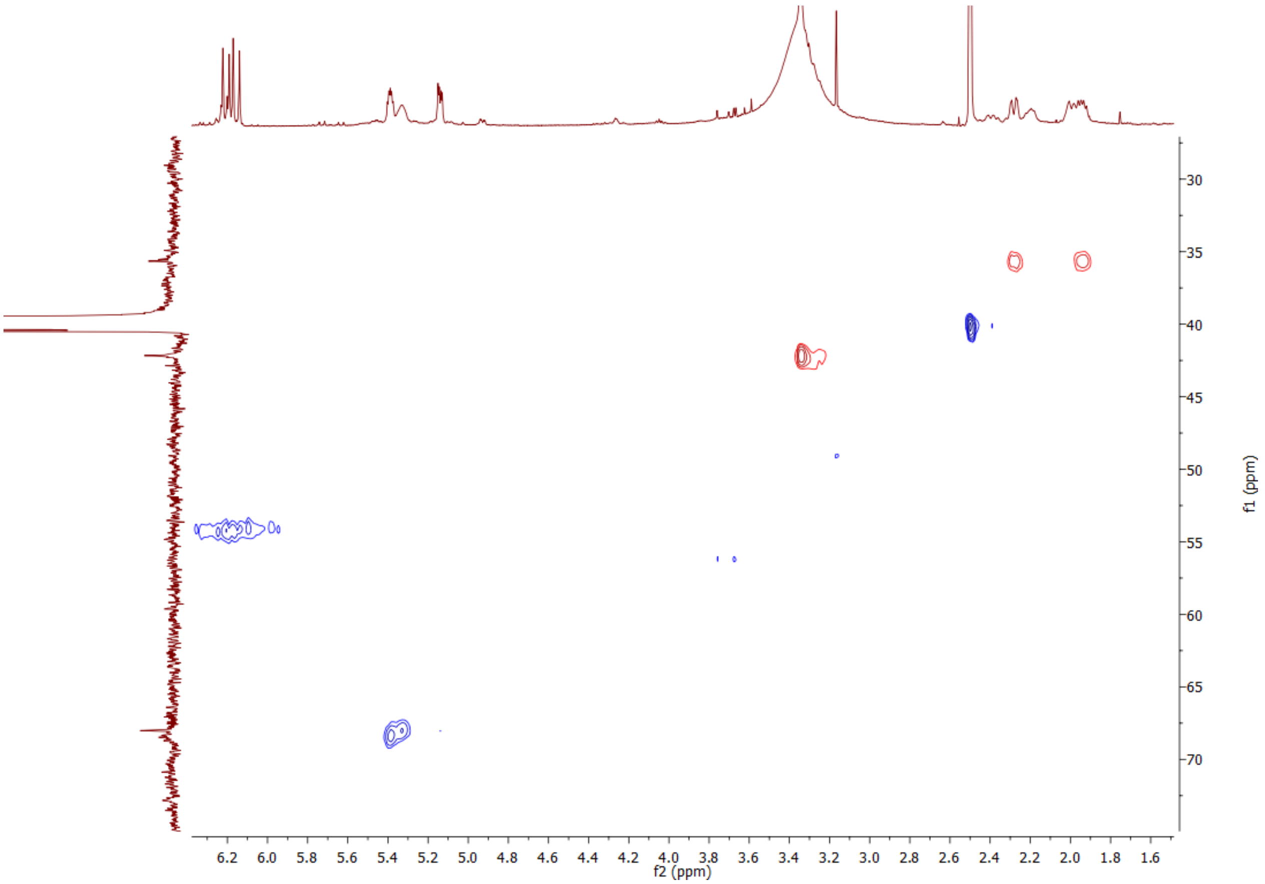

Figure S59. ^{1}H-^{13}C HSQC (DMSO-d_6, 500/125 MHz) spectrum of compound **6** for aliphatic region.

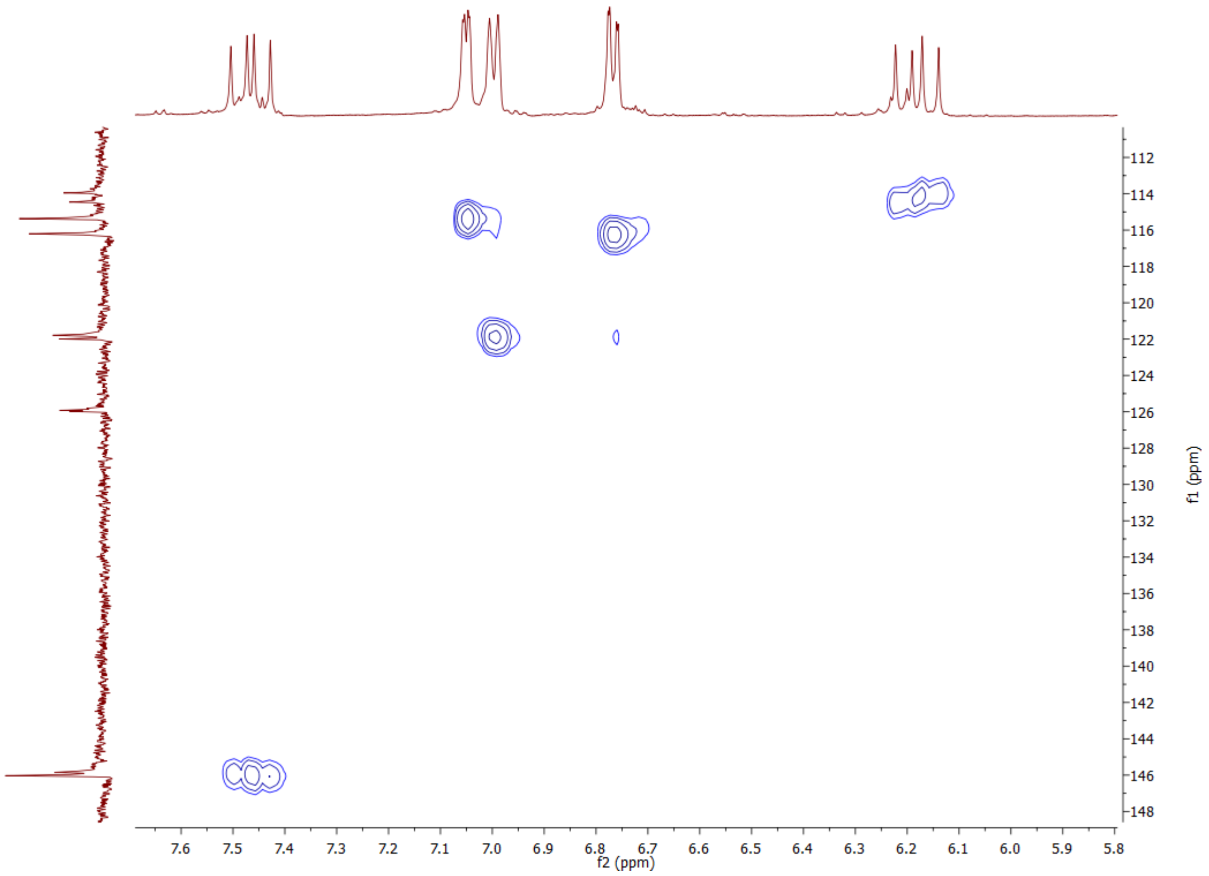

Figure S60. ^{1}H-^{13}C HSQC (DMSO-d_6, 500/125 MHz) spectrum of compound **6** for aromatic region.

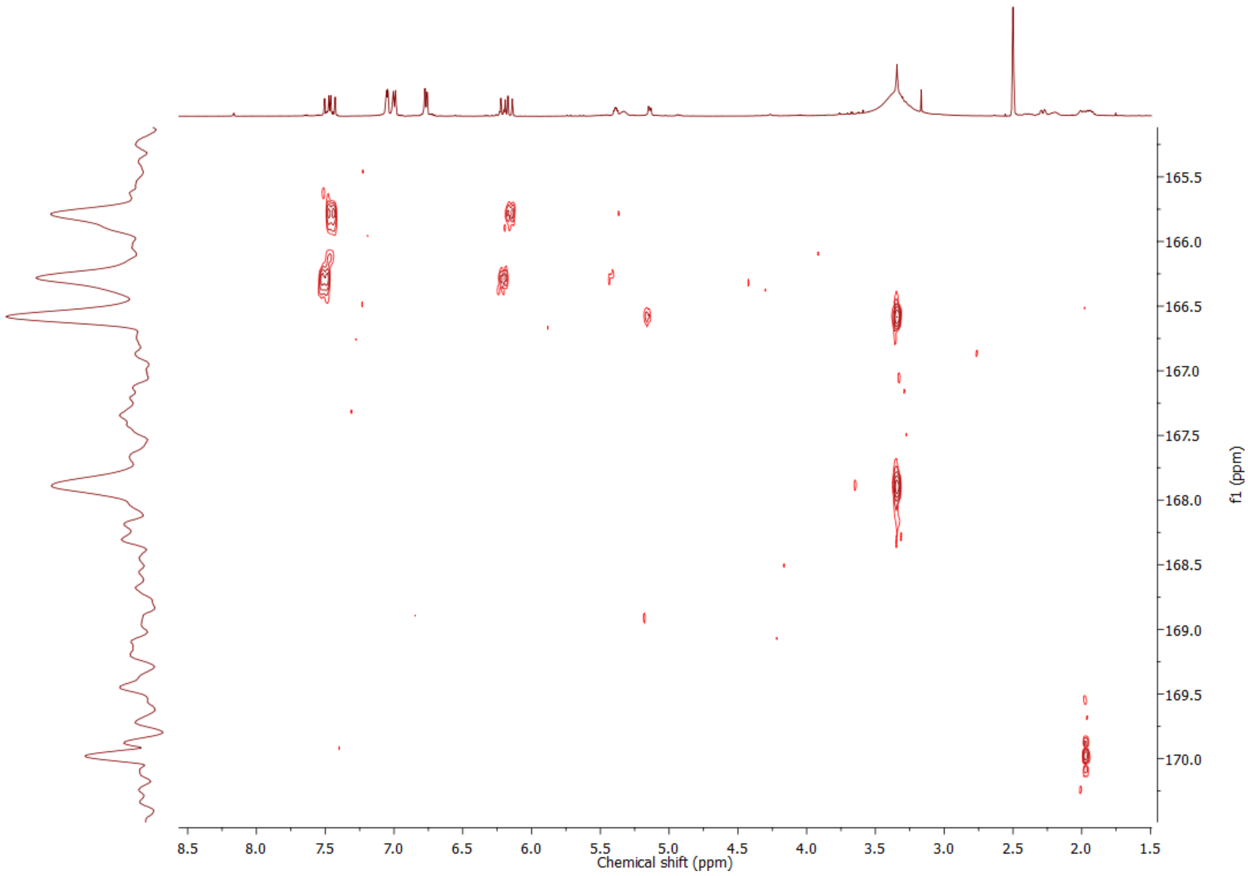

Figure S61. Band-selective ^{1}H-^{13}C HMBC (DMSO-d_6, 500/125 MHz) spectrum of compound **6** recorded at 328 Kelvin.

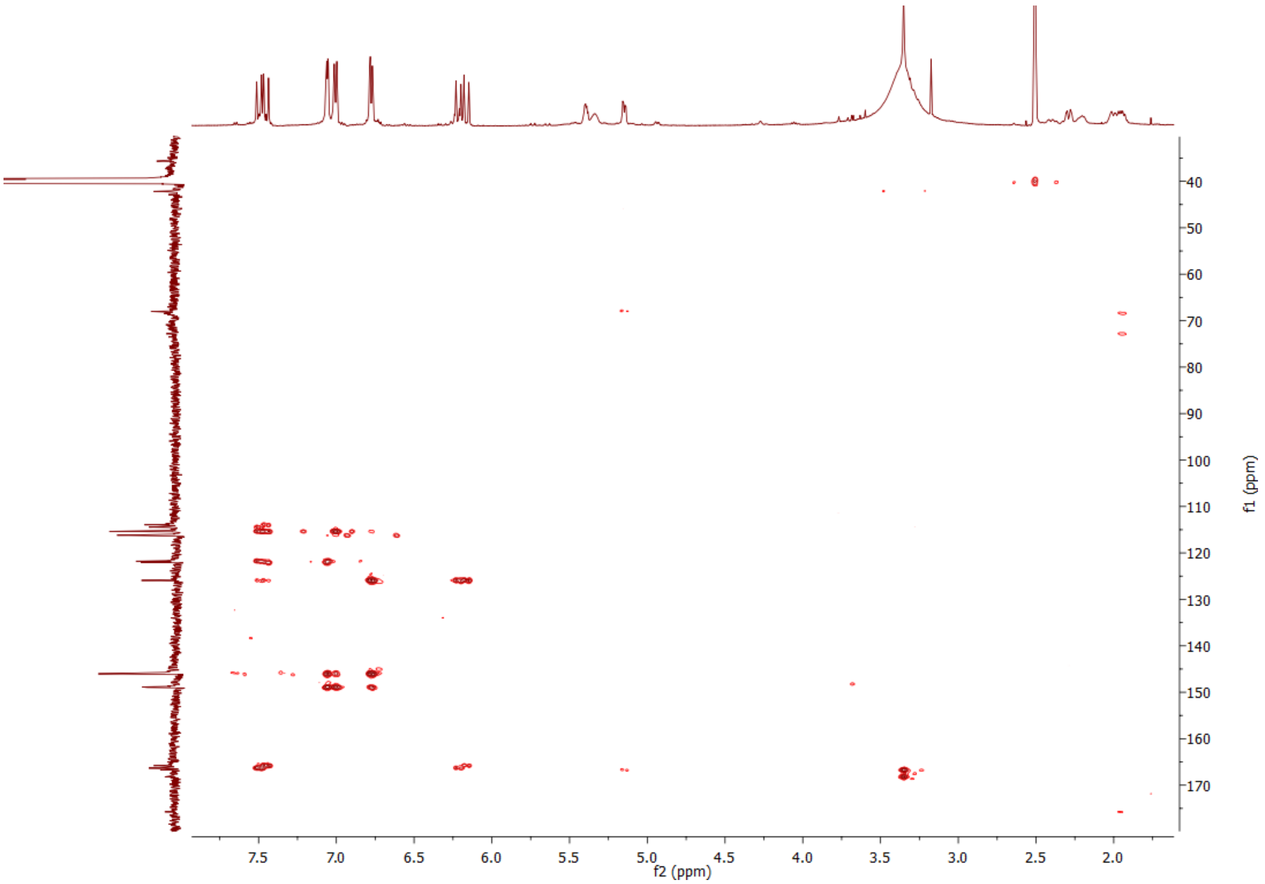

Figure S62. ^{1}H-^{13}C HMBC (DMSO-d_6, 500/125 MHz) spectrum of compound **6**.

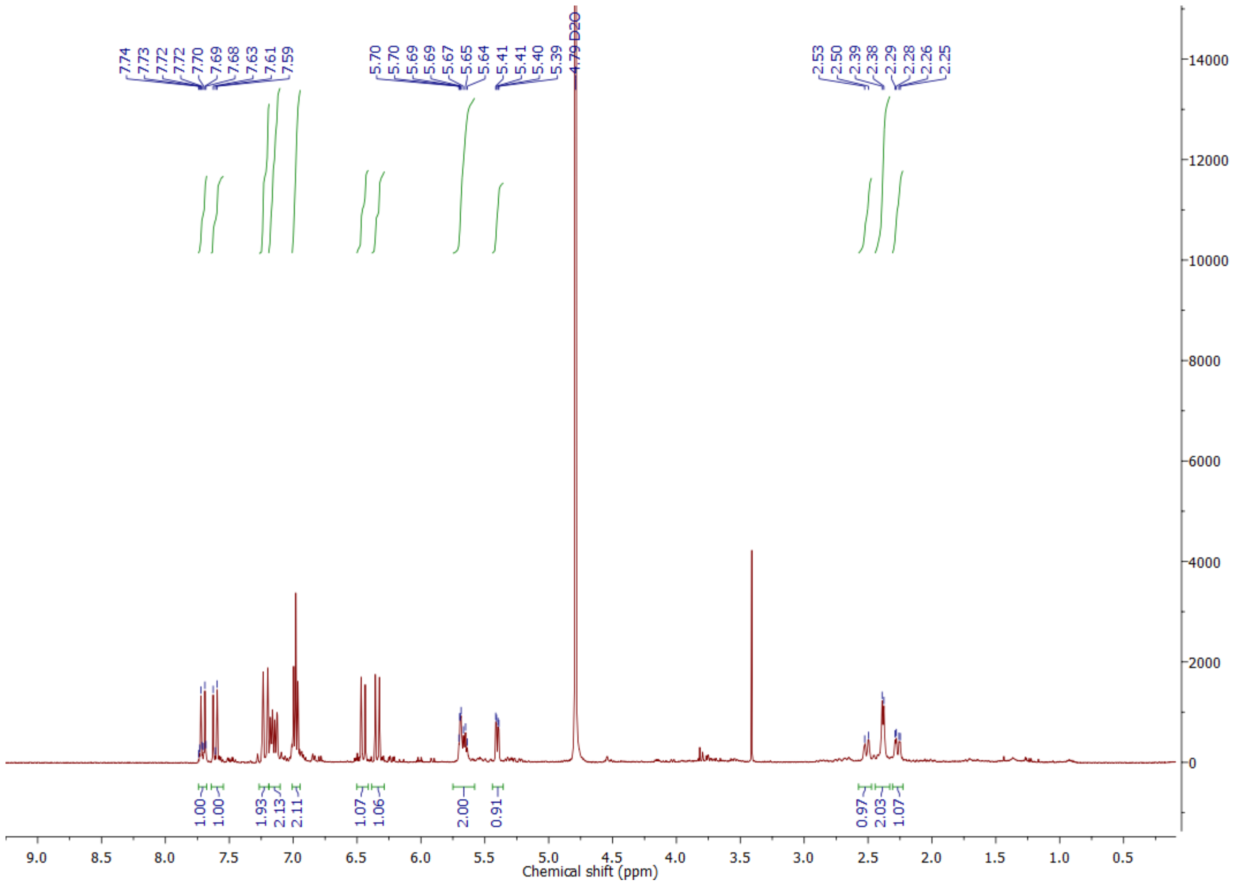

Figure S63. ^1H NMR (D_2O, 500 MHz) spectrum of compound **6**.

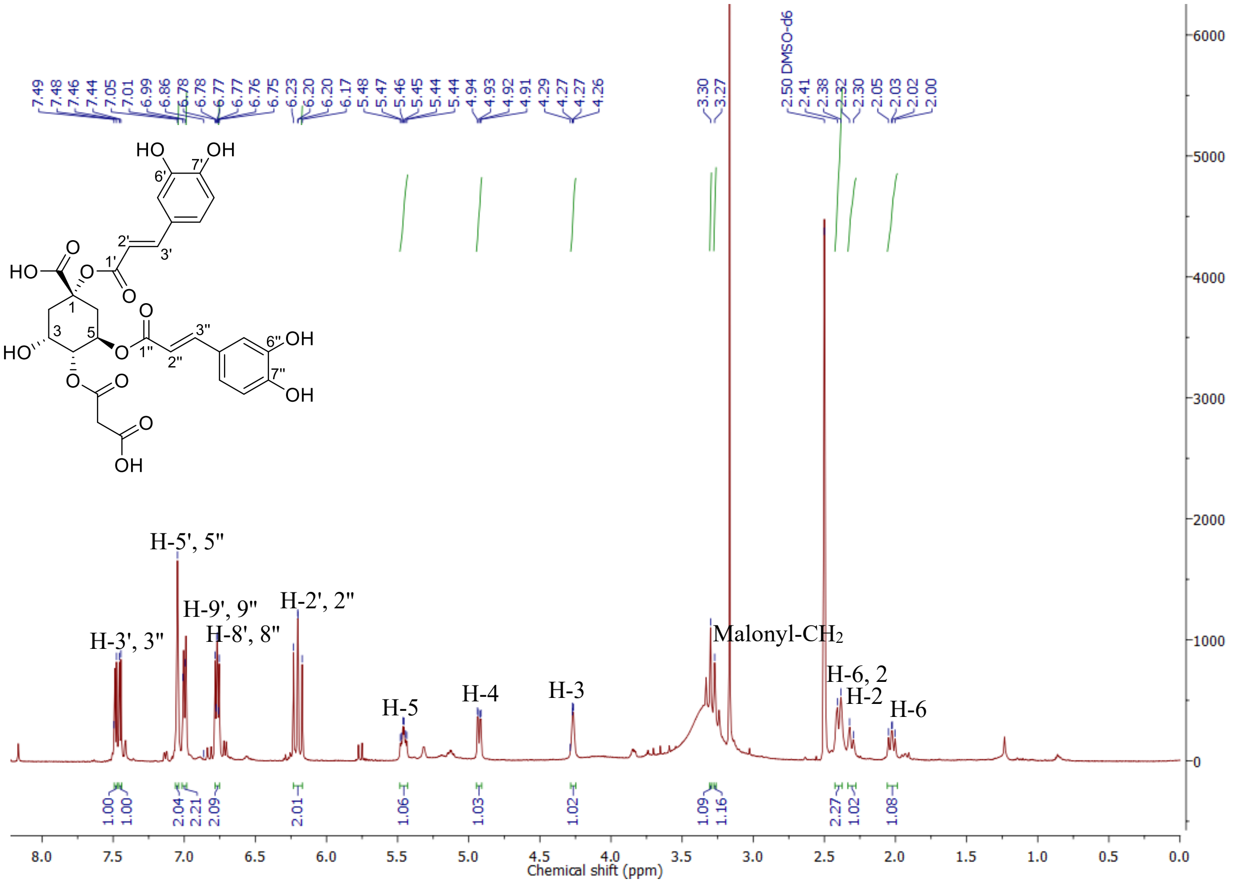

Figure S64. ^{1}H NMR (DMSO-d_6, 500 MHz) spectrum of compound **7**.

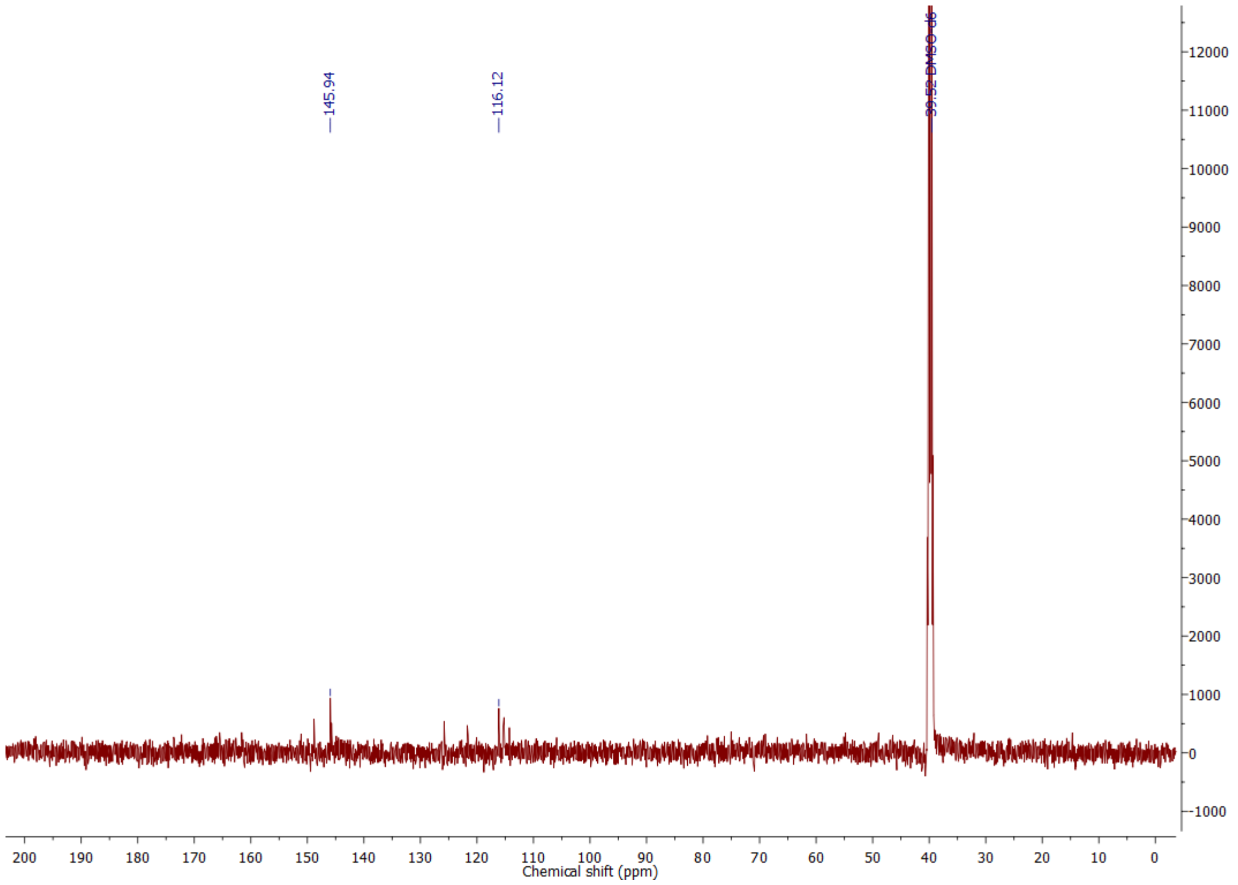

Figure S65. ^{13}C NMR (DMSO-d_6, 500 MHz) spectrum of compound **7**.

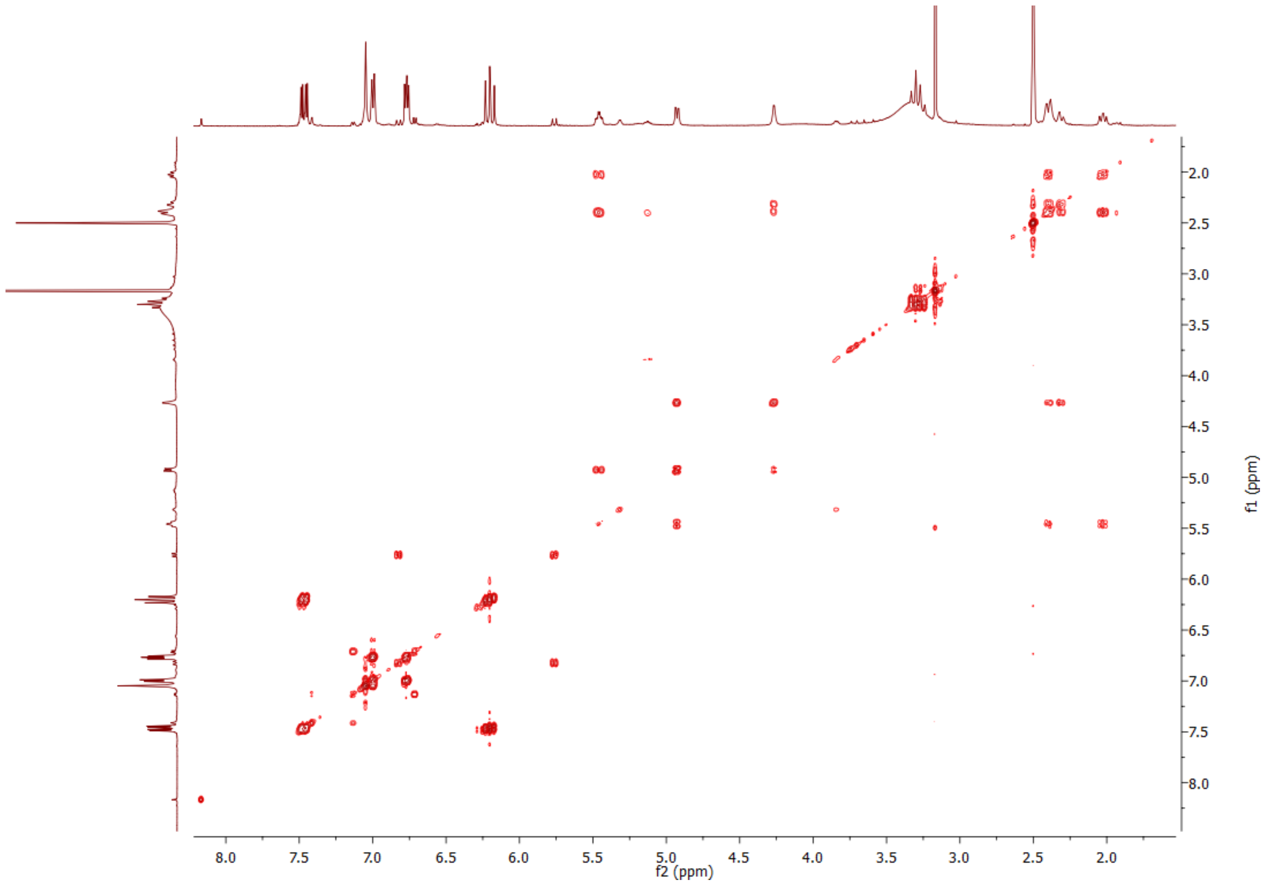

Figure S66. ^{1}H-^{1}H COSY (DMSO-d_6, 500 MHz) spectrum of compound **7**.

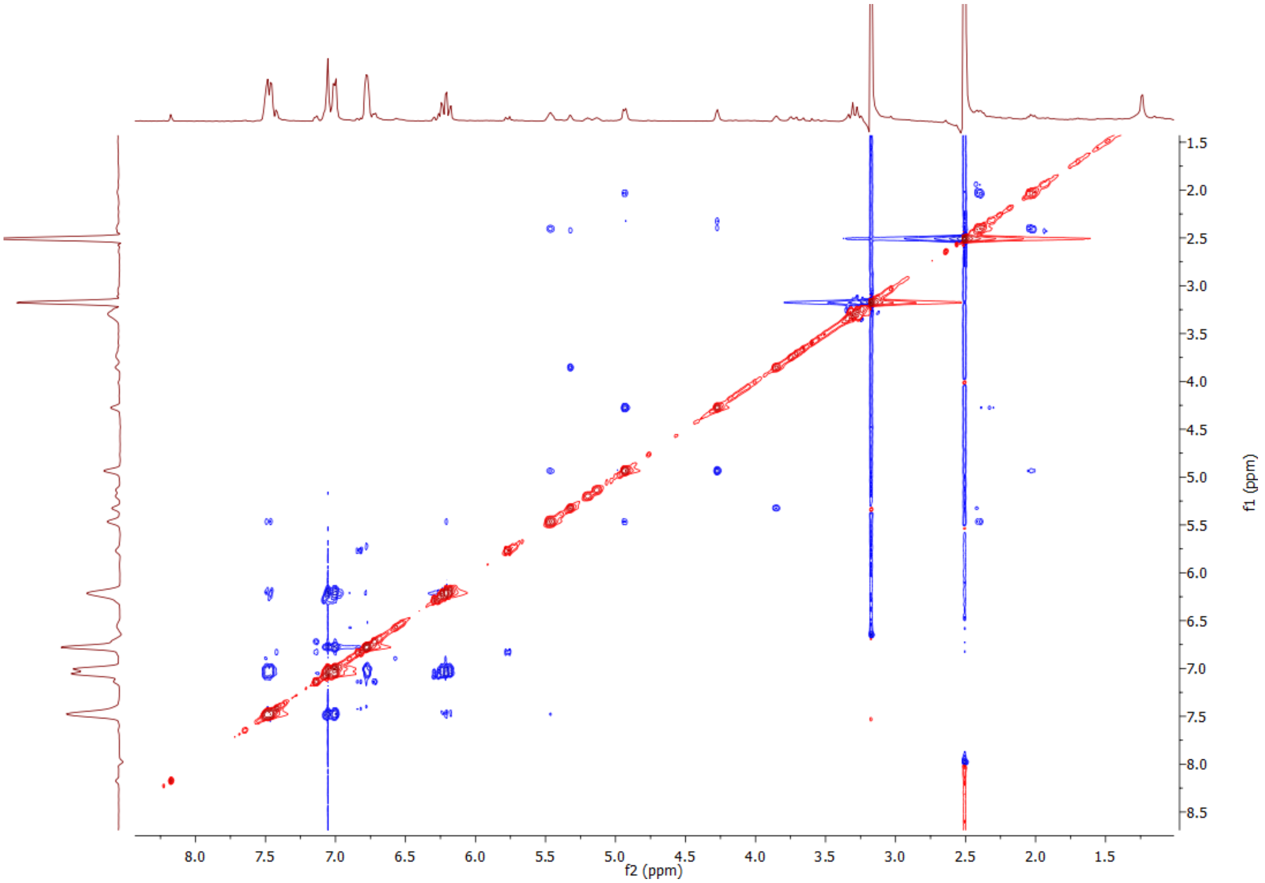

Figure S67. ^{1}H-^{1}H ROESY (DMSO-d_6, 500 MHz) spectrum of compound **7**.

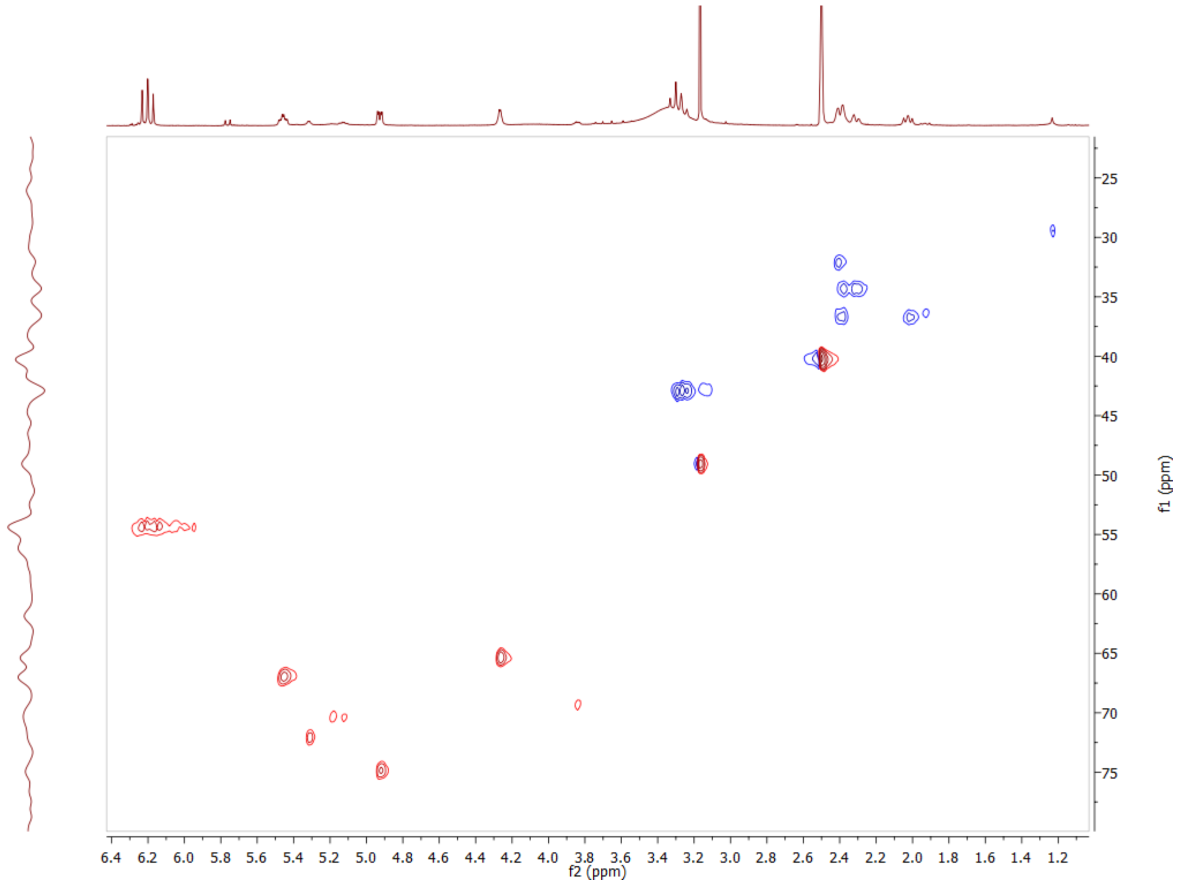

Figure S68. ^{1}H-^{13}C HSQC (DMSO-d_6, 500/125 MHz) spectrum of compound **7** for aliphatic region.

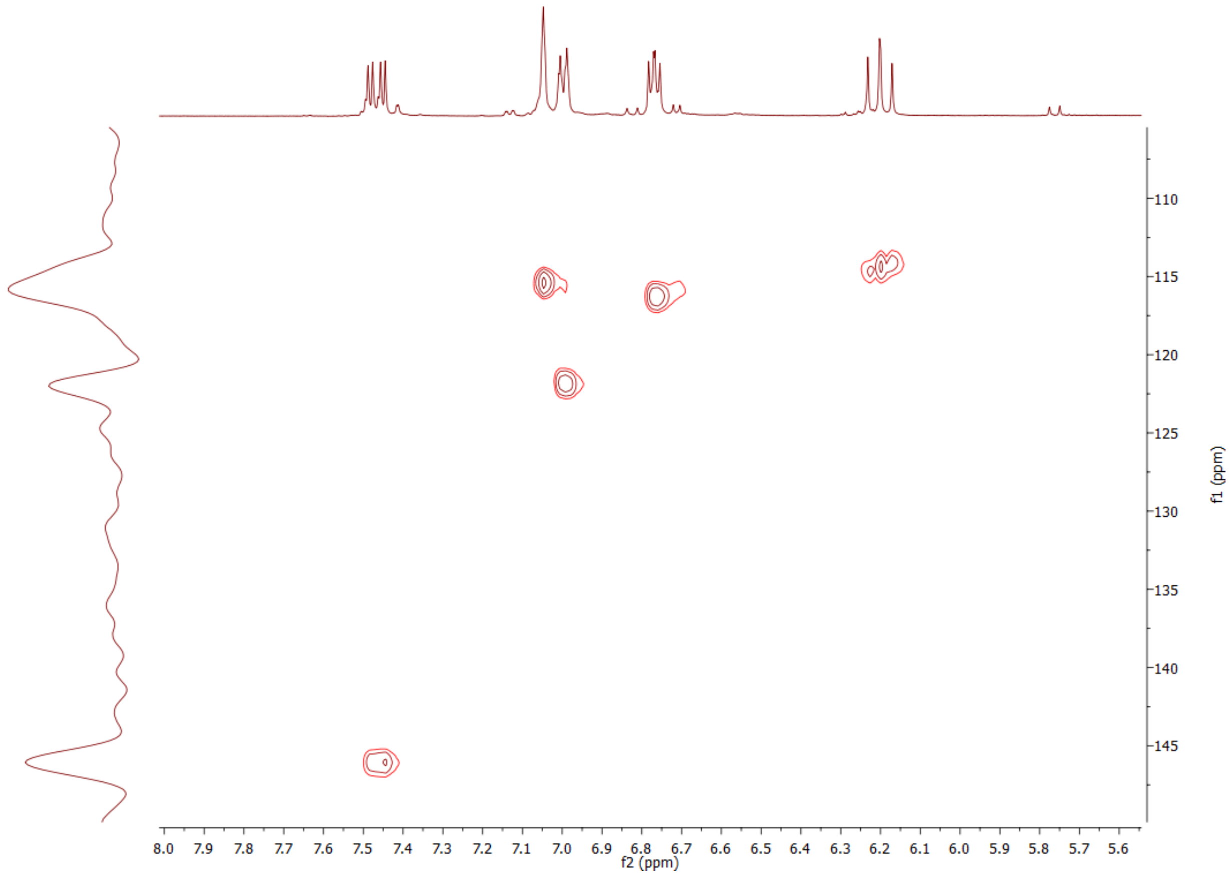

Figure S69. ^{1}H-^{13}C HSQC (DMSO-d_6, 500/125 MHz) spectrum of compound **7** for aromatic region.

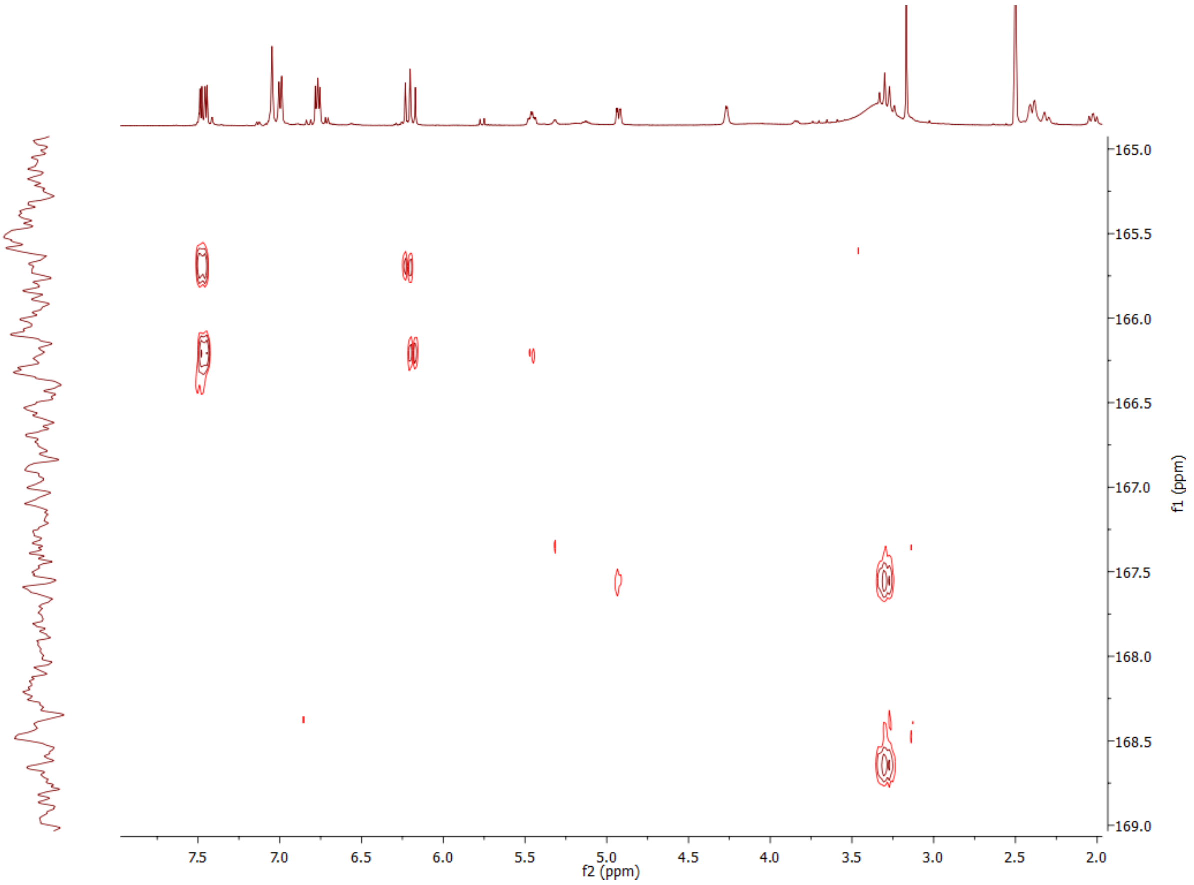

Figure S70. Band-selective ^{1}H-^{13}C HMBC (DMSO-d_6, 500/125 MHz) spectrum of compound **7**.

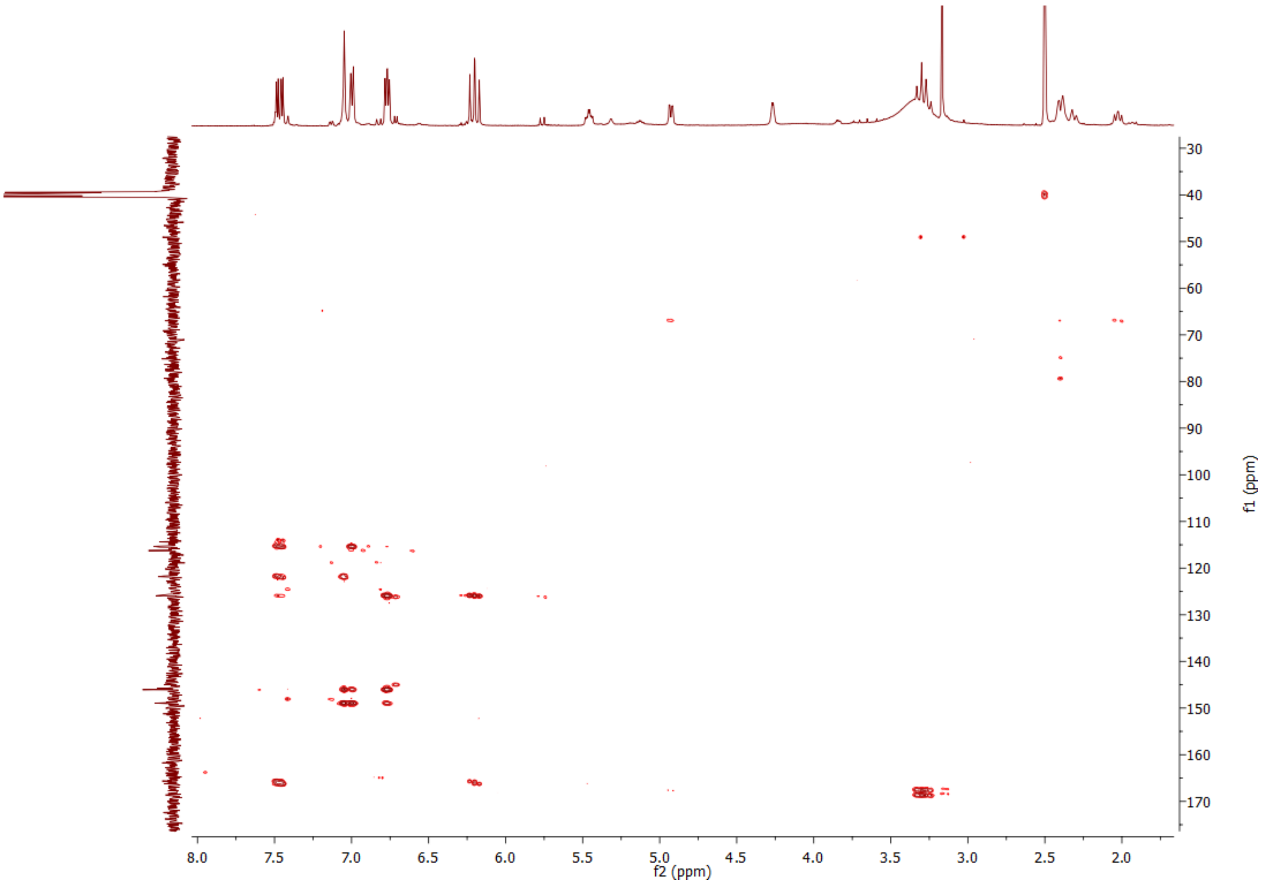

Figure S71. Full ^{1}H-^{13}C HMBC (DMSO-d_6, 500/125 MHz) spectrum of compound **7**.

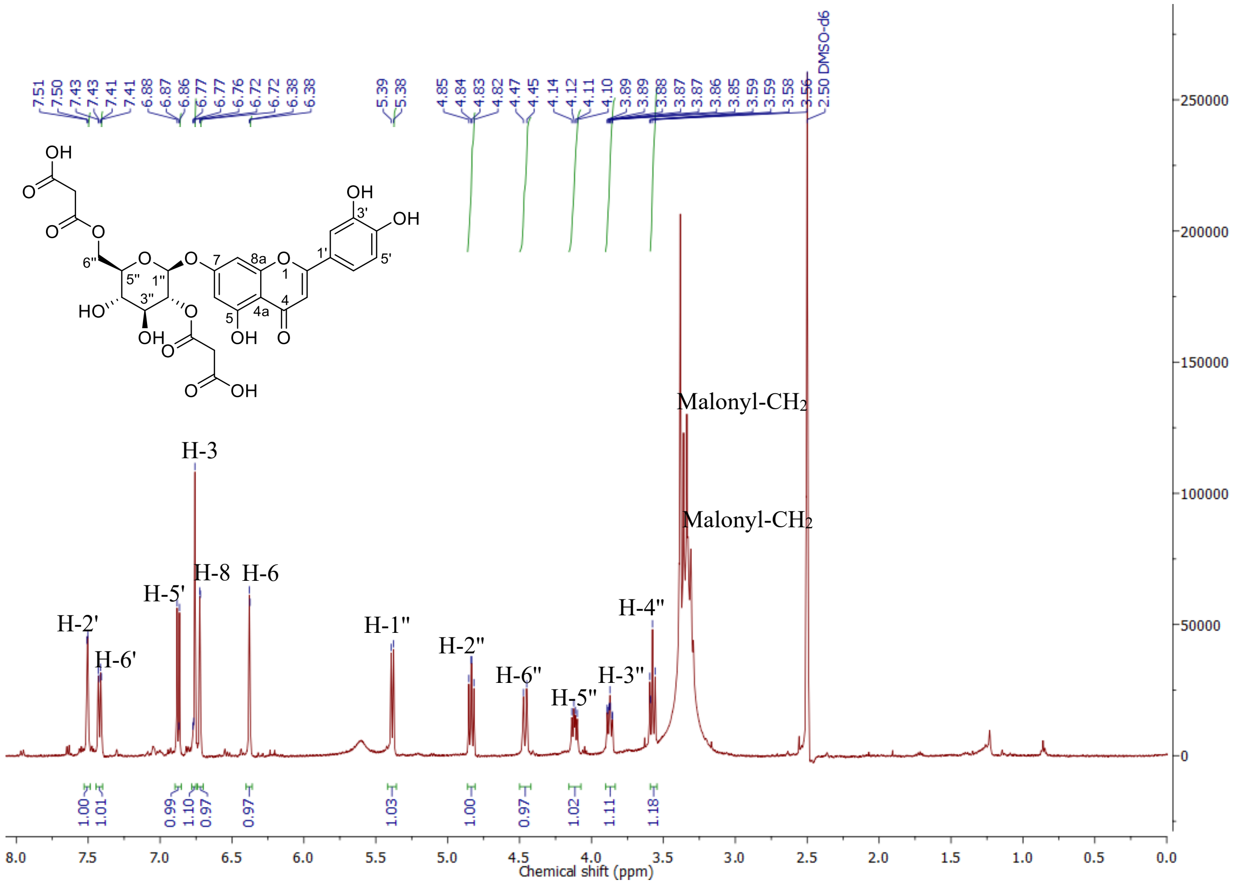

Figure S72. ^{1}H NMR (DMSO-d_6, 500 MHz) spectrum of compound **8**.

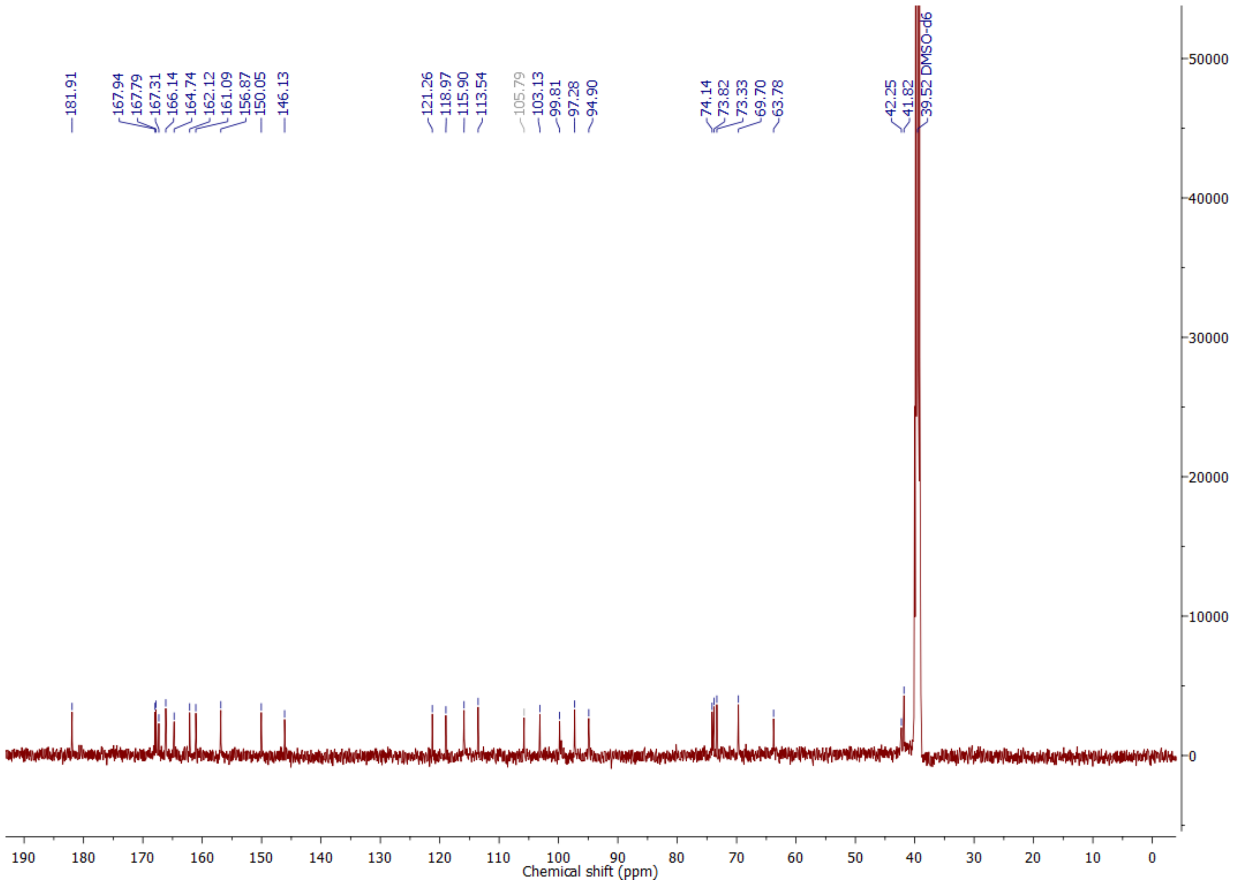

Figure S73. ^{13}C NMR (DMSO-d_6, 125 MHz) spectrum of compound **8**.

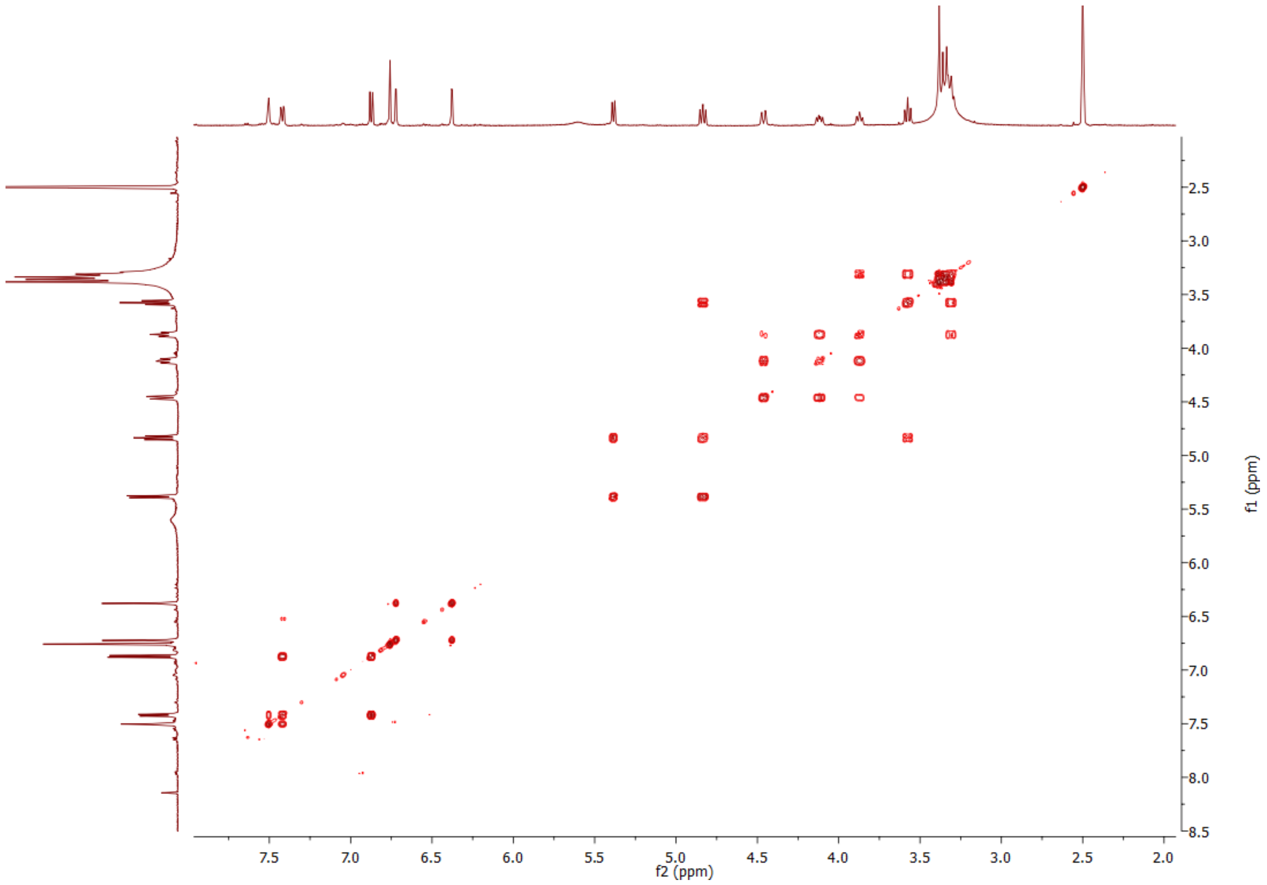

Figure S74. ^{1}H-^{1}H COSY (DMSO-d_6, 500 MHz) spectrum of compound **8**.

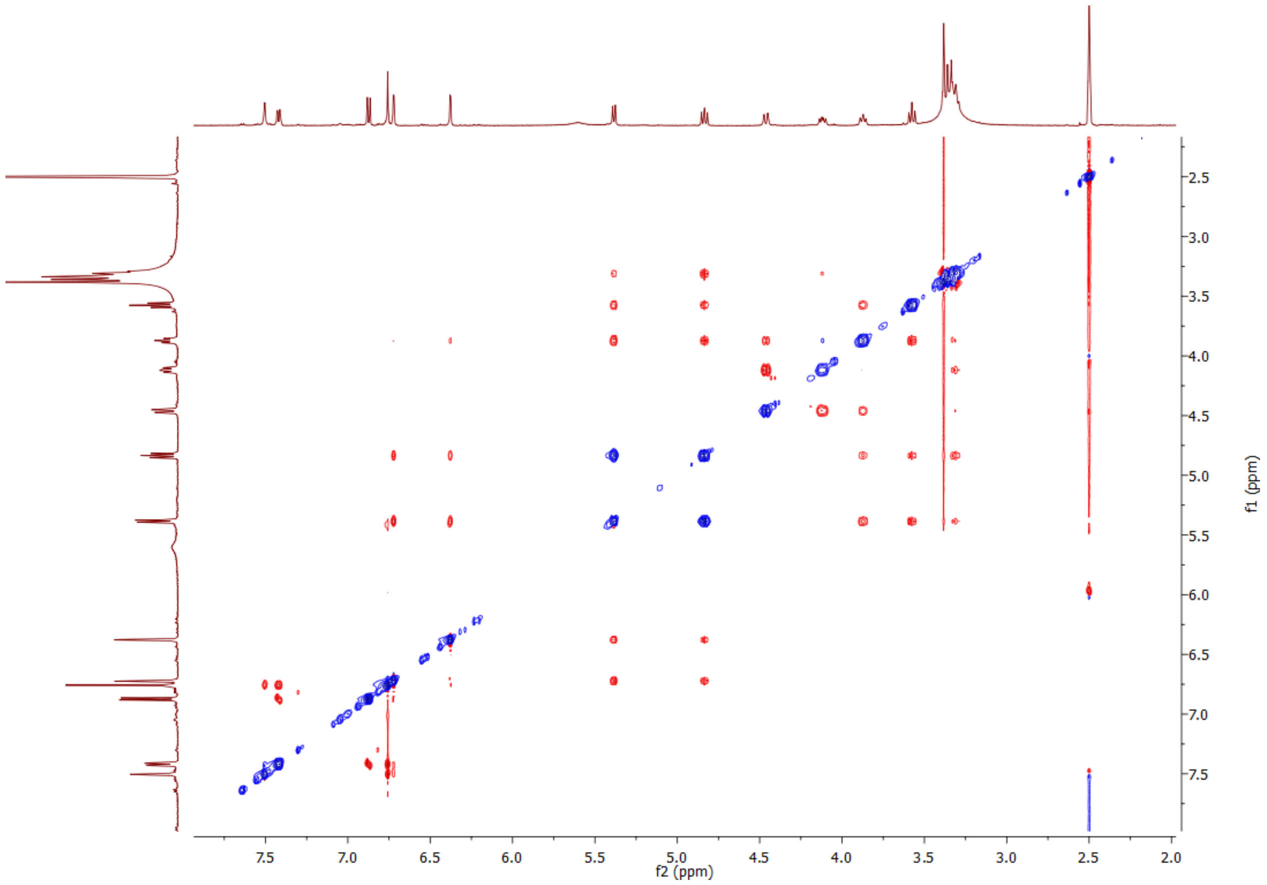

Figure S75. ^{1}H-^{1}H ROESY (DMSO-d_6, 500 MHz) spectrum of compound **8**.

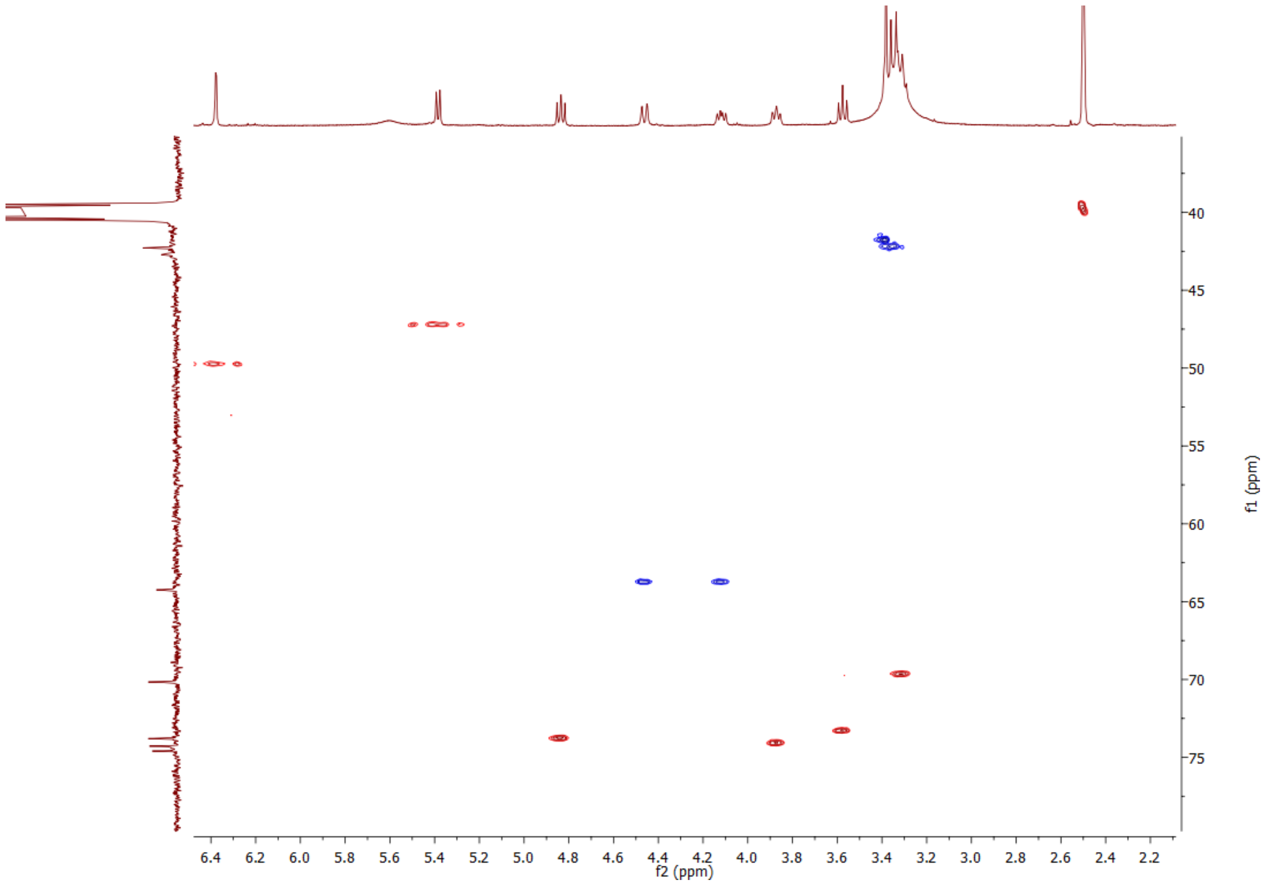

Figure S76. ^{1}H-^{13}C HSQC (DMSO-d_6, 500/125 MHz) spectrum of compound **8** for aliphatic region.

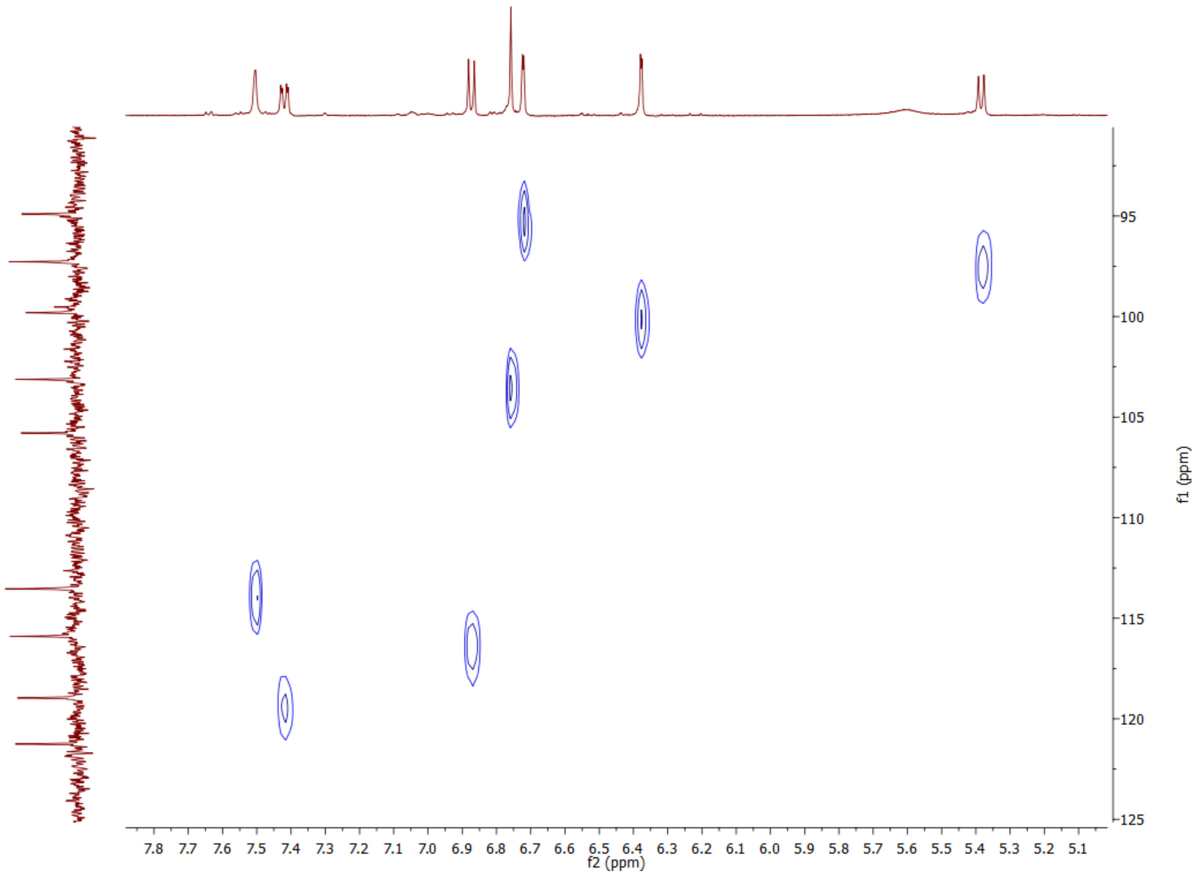

Figure S77. ^{1}H-^{13}C HSQC (DMSO-d_6, 500/125 MHz) spectrum of compound **8** for aromatic region.

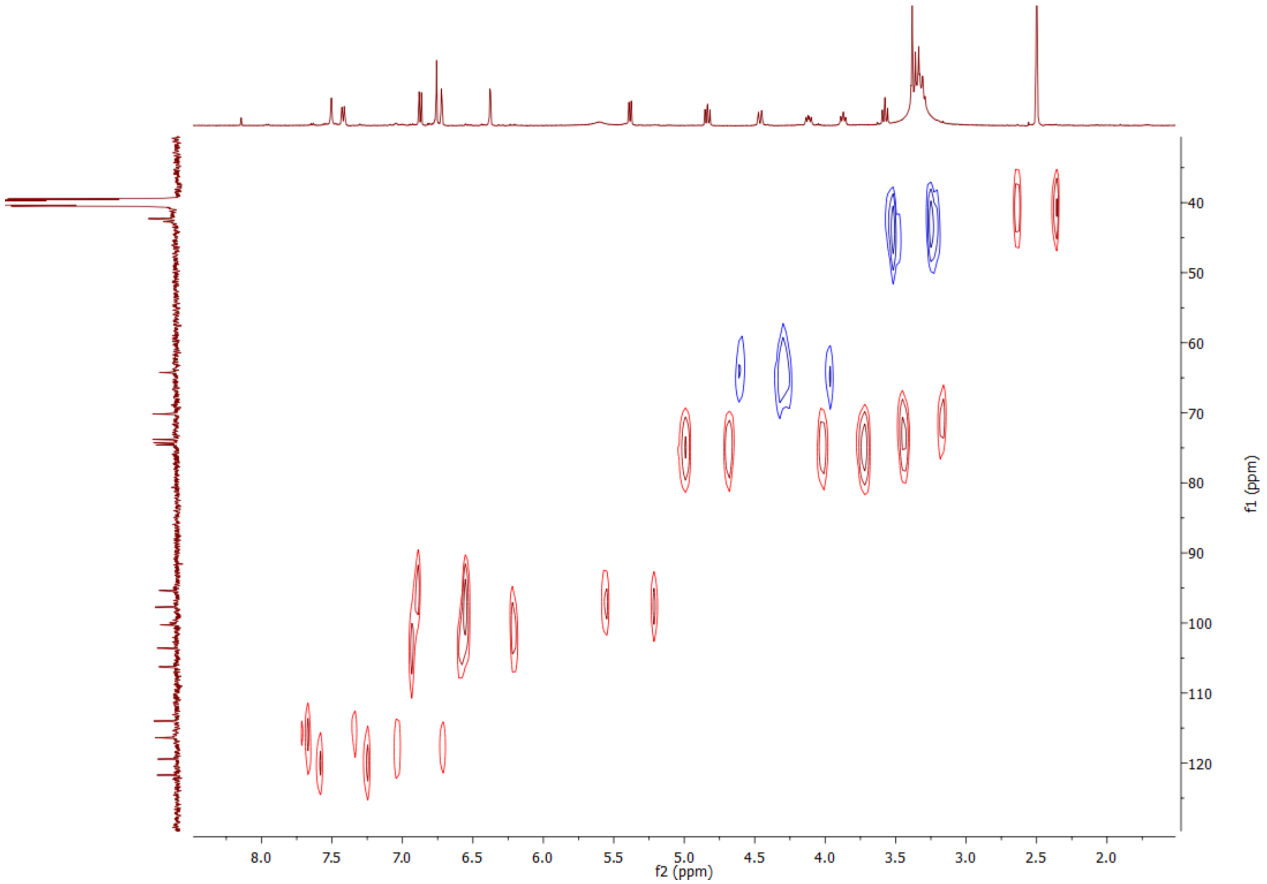

Figure S78. ^{1}H-^{13}C HSQC (DMSO-d_6, 500/125 MHz) spectrum (not decoupled) of compound **8**.

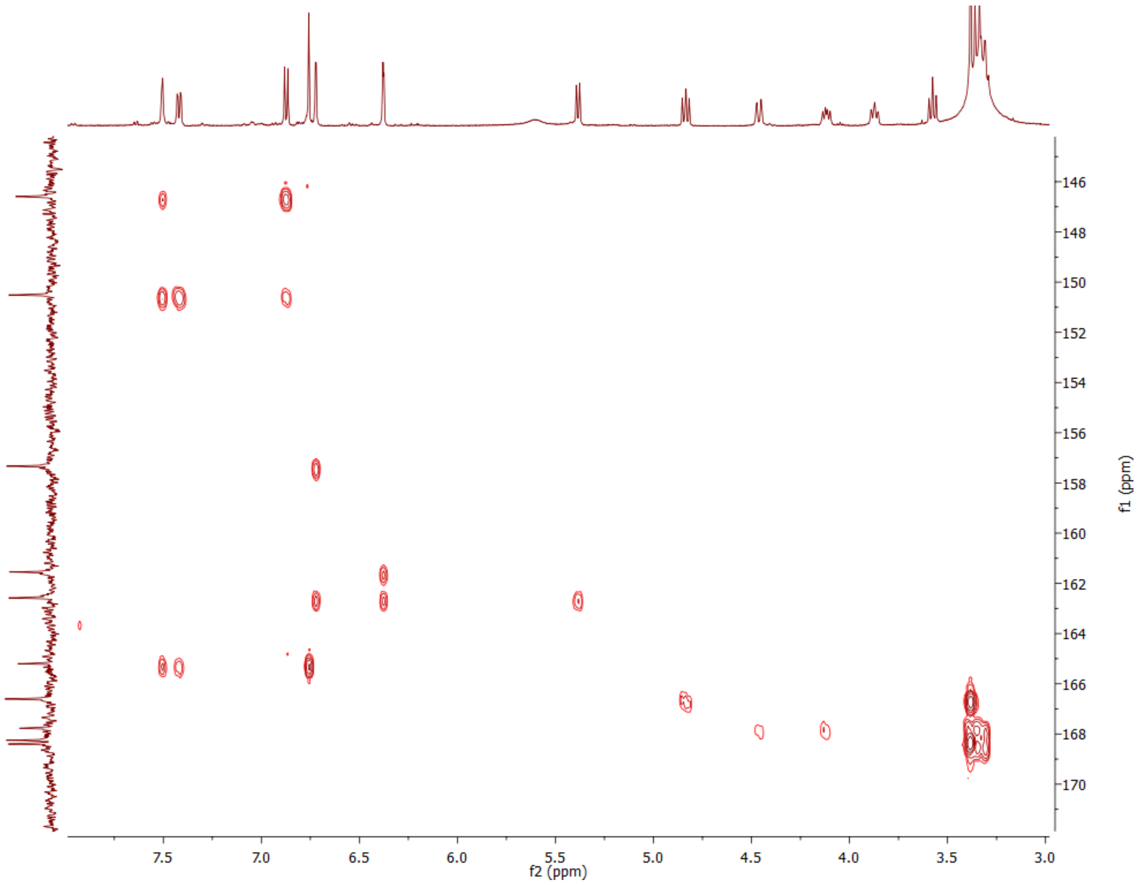

Figure S79. ^{1}H-^{13}C HMBC (DMSO-d_6, 500/125 MHz) spectrum of compound **8**.

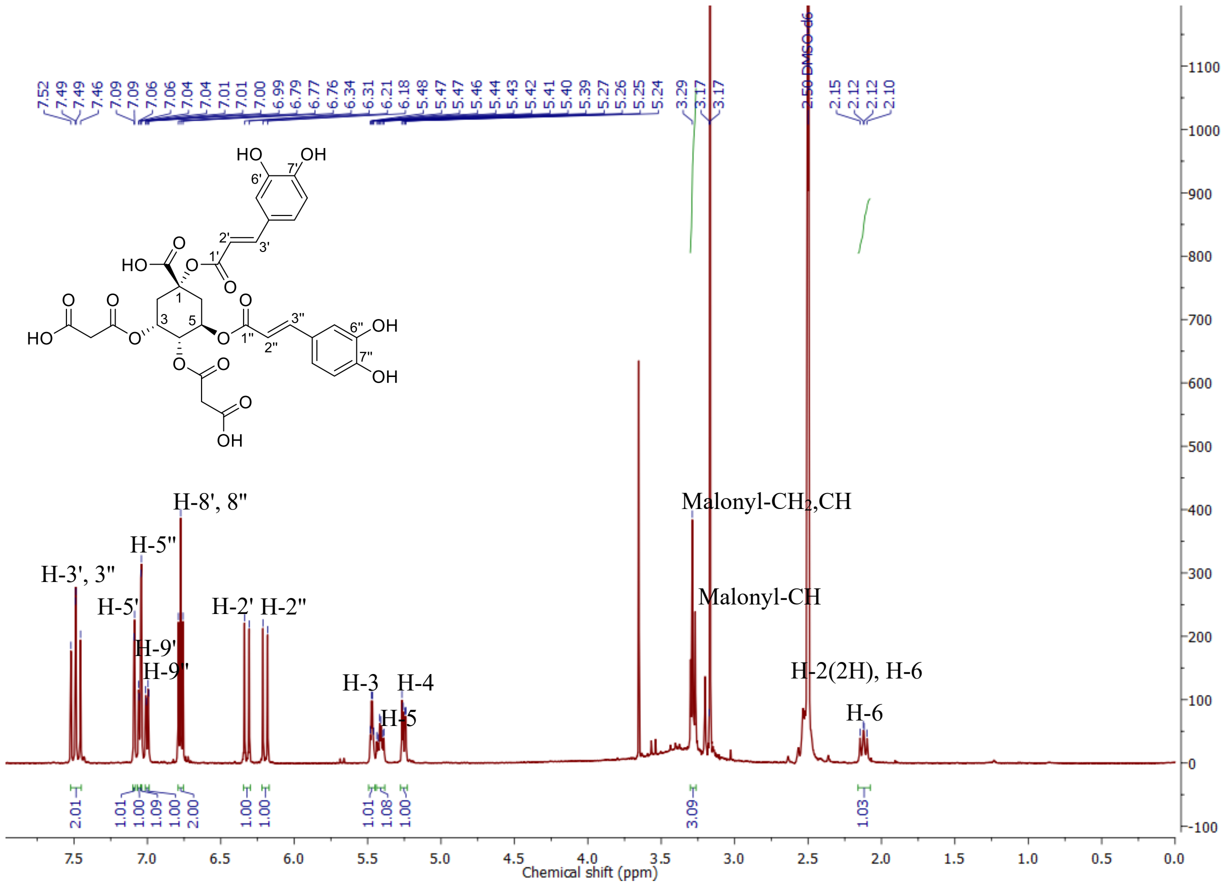

Figure S80. ^{1}H NMR (DMSO-d_6, 500 MHz) spectrum of compound **9**.

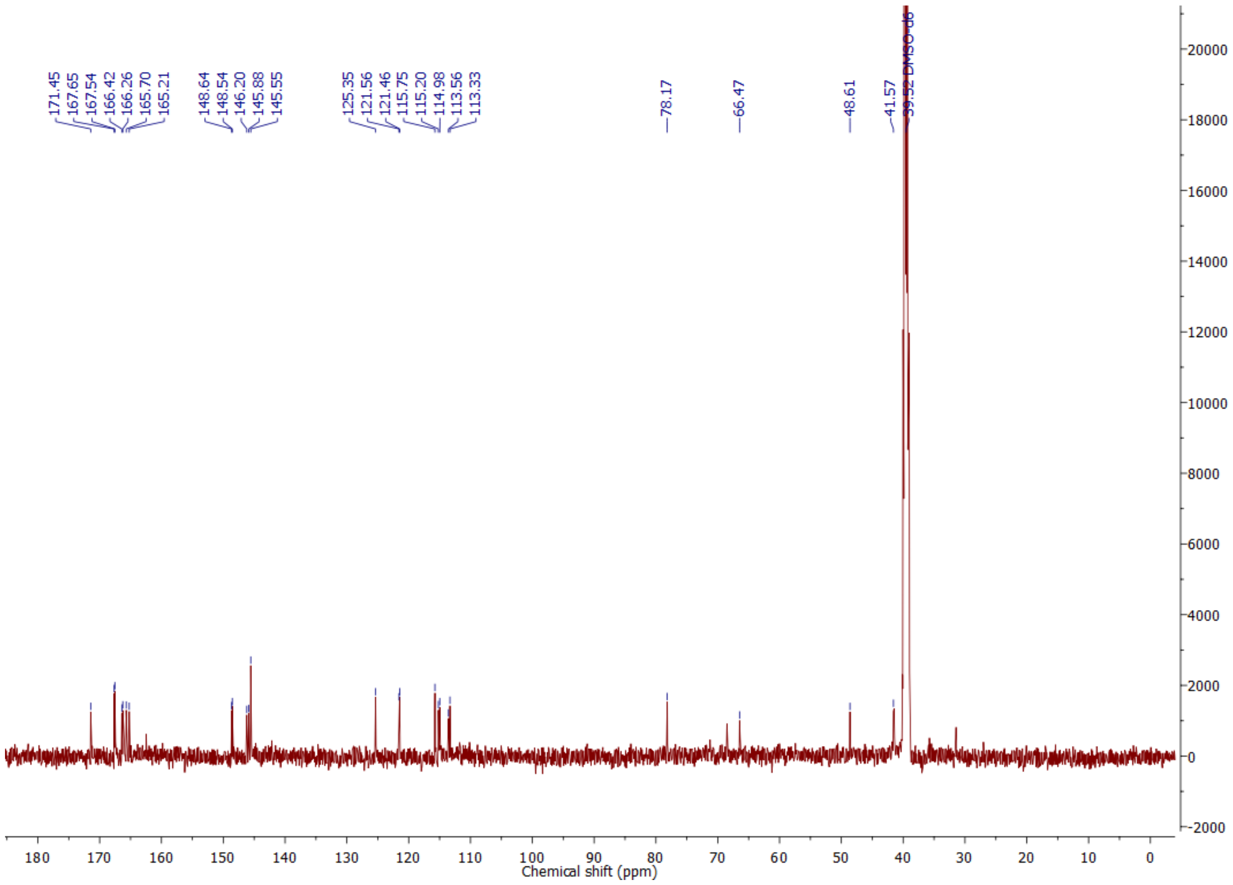

Figure S81. ^{13}C NMR (DMSO-d_6, 125 MHz) spectrum of compound **9**.

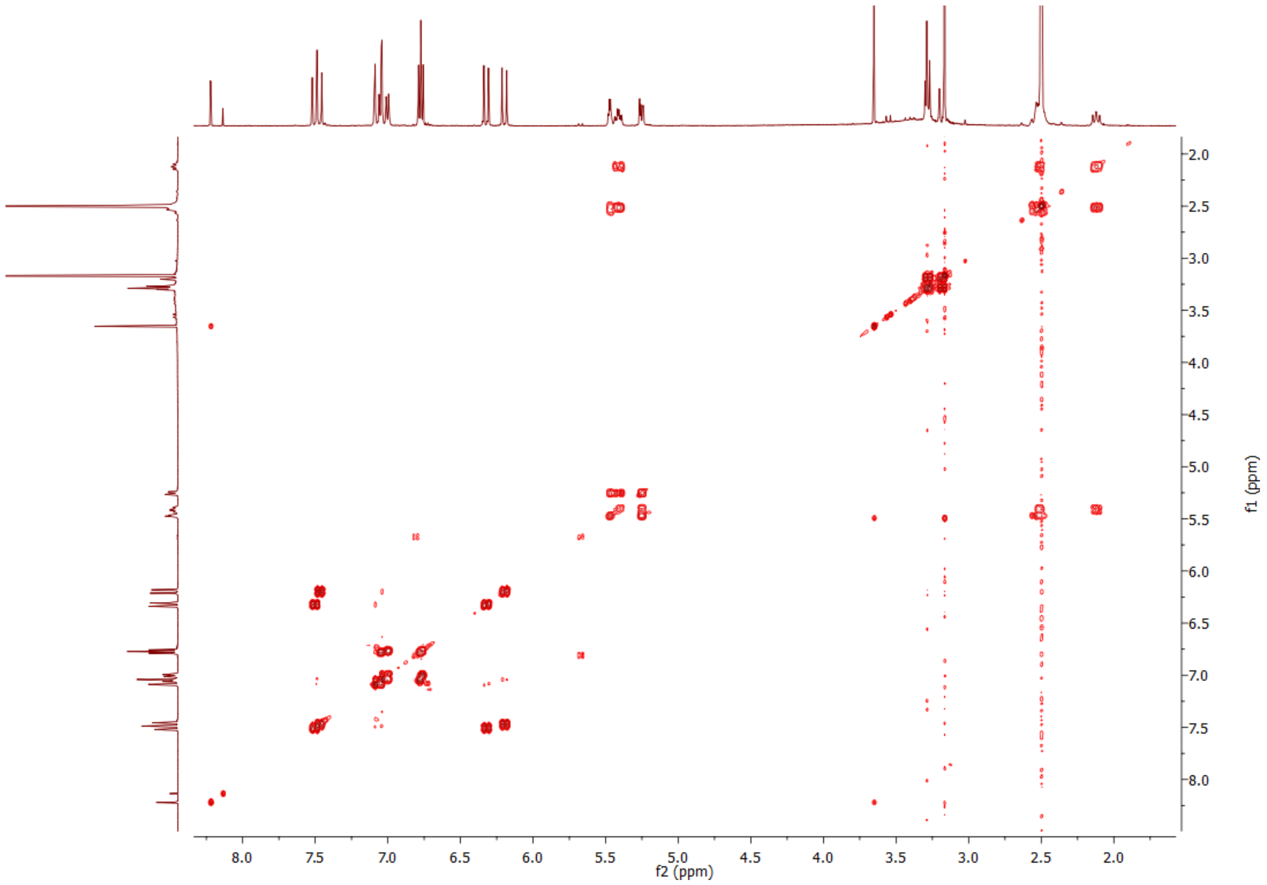

Figure S82. ^{1}H-^{1}H COSY (DMSO-d_6, 500 MHz) spectrum of compound **9**.

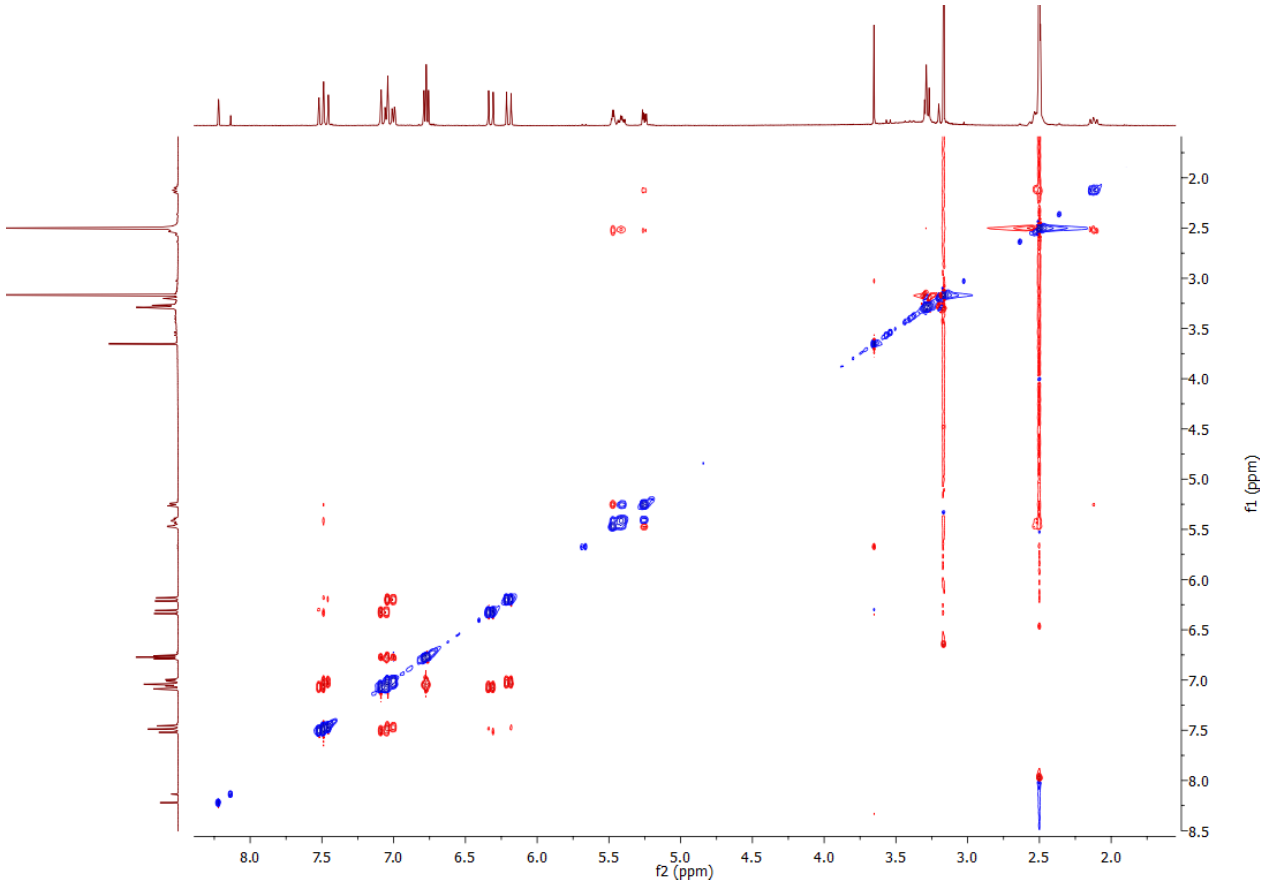

Figure S83. ^{1}H-^{1}H ROESY (DMSO-d_6, 500 MHz) spectrum of compound **9**.

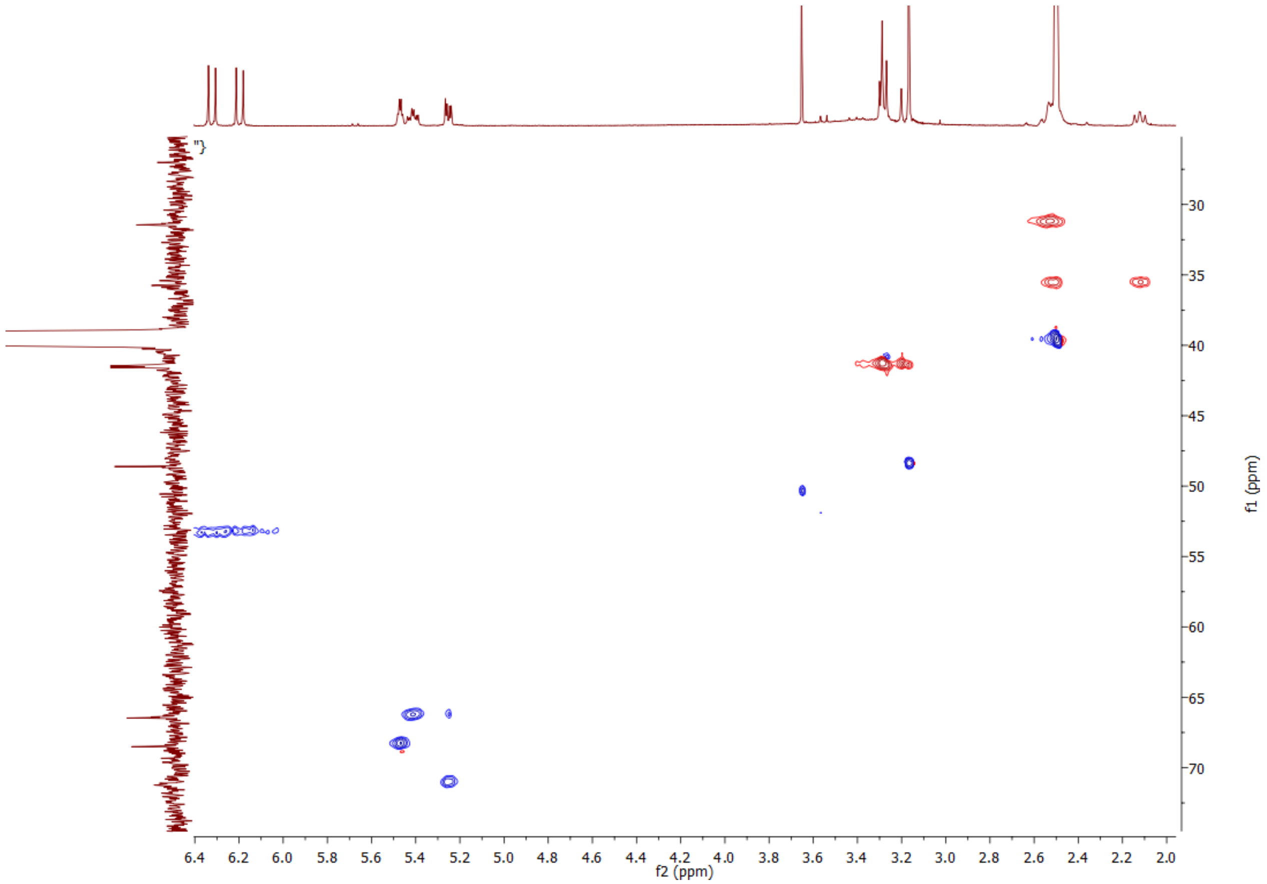

Figure S84. ^{1}H-^{13}C HSQC (DMSO-d_6, 500/125 MHz) spectrum of compound **9** for aliphatic region.

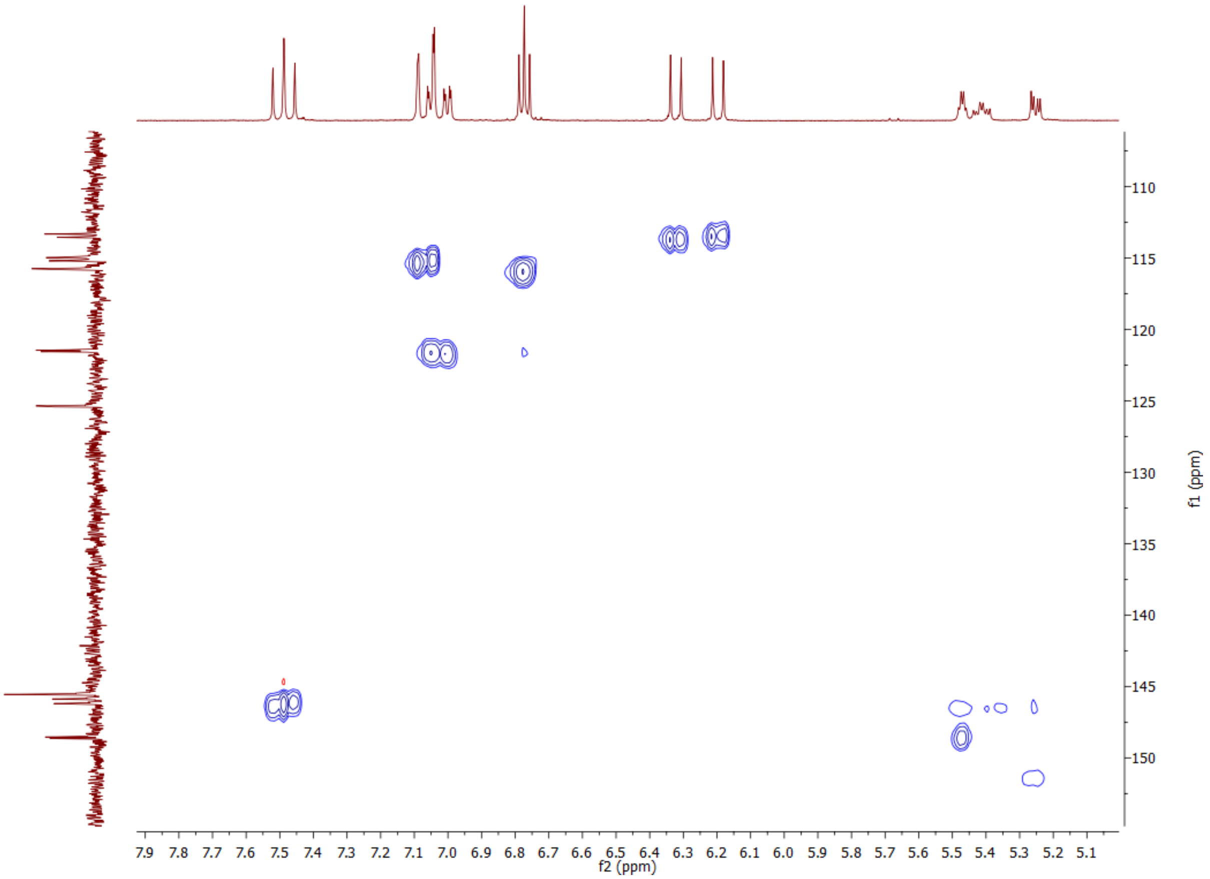

Figure S85. ^{1}H-^{13}C HSQC (DMSO-d_6, 500/125 MHz) spectrum of compound **9** for aromatic region.

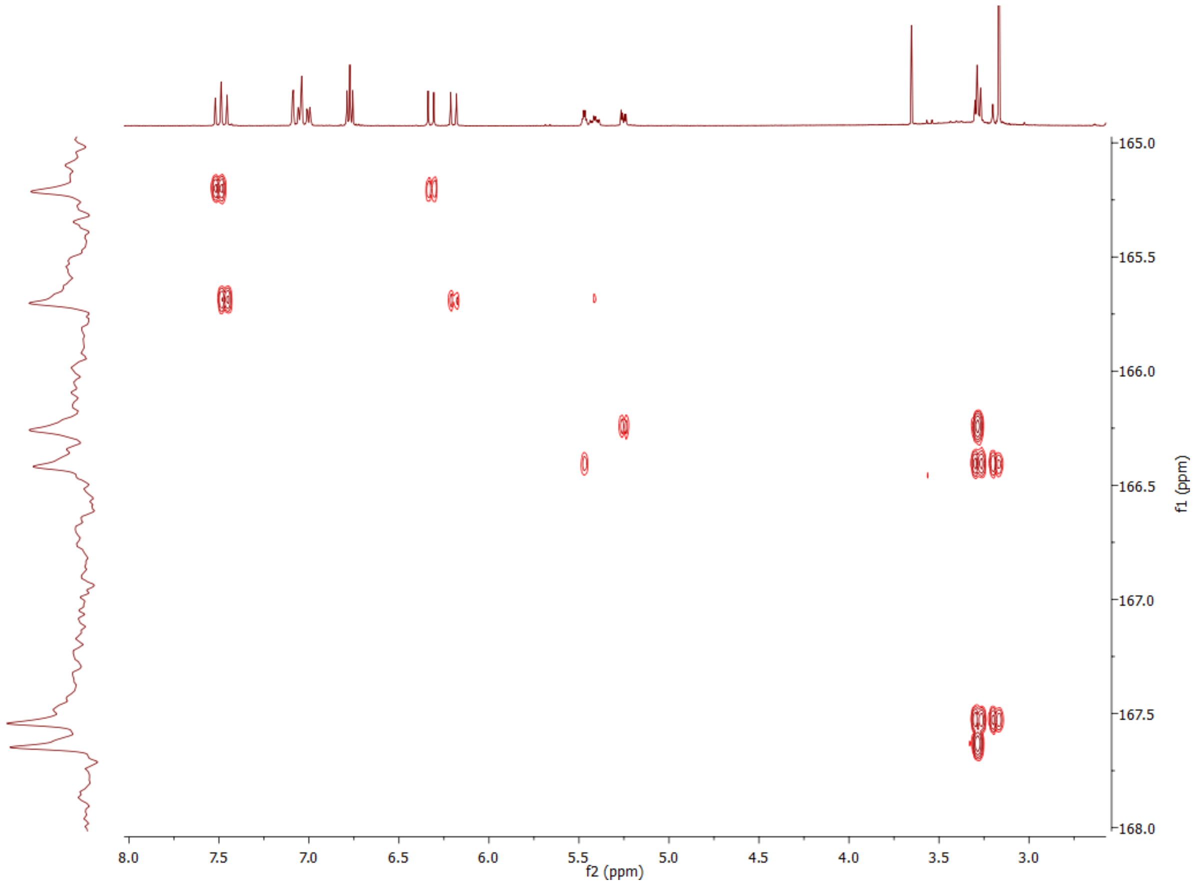

Figure S86. Band-selective ^{1}H-^{13}C HMBC (DMSO-d_6, 500/125 MHz) spectrum of compound **9**.

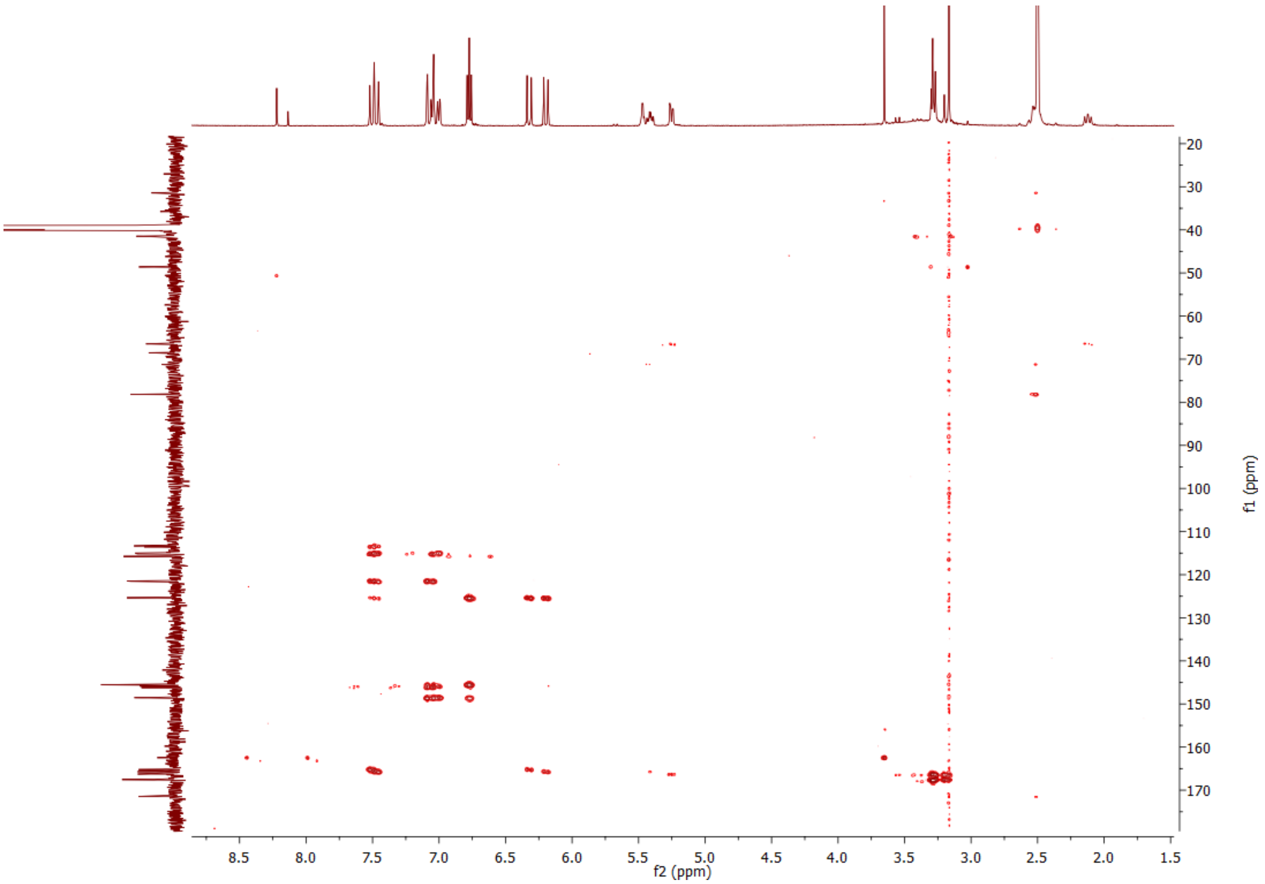

Figure S87. Full ^{1}H-^{13}C HMBC (DMSO-d_6, 500/125 MHz) spectrum of compound **9**.
